# Comparison of Different PCI Strategies for Coronary DES In-stent Restenosis: A Bayesian Network Meta-analysis

**DOI:** 10.1016/j.jscai.2024.102428

**Published:** 2025-01-31

**Authors:** Prakash Raj Oli, Dhan Bahadur Shrestha, Sagun Dawadi, Shraddha Poudel, Furkhan Ali, Jurgen Shtembari, Kailash Pant, Bishesh Shrestha, Rafay Khan, Jishanth Mattumpuram, Daniel H. Katz

**Affiliations:** a Department of Internal Medicine, Mount Sinai Hospital, Chicago, Illinois; b Division of Cardiology, Department of Internal Medicine, Bassett Medical Center, Cooperstown, New York; c Department of Internal Medicine, Nepalese Army Institute of Health Sciences, Kathmandu, Nepal; d Department of Internal Medicine, Medstar Union Memorial Hospital, Baltimore, Maryland; e Division of Cardiology, Department of Internal Medicine, Carle Foundation Hospital, Urbana, Illinois; f Division of Cardiology, Department of Internal Medicine, UMass Chan Medical School Baystate Campus, Springfield, Massachusetts; g Division of Cardiology, Department of Internal Medicine, University of Nebraska, Omaha, Nebraska; h Division of Cardiology, Department of Internal Medicine, University of Louisville School of Medicine, Louisville, Kentucky

**Keywords:** drug-coated balloon, drug-eluting stents, in-stent restenosis

## Abstract

**Background:**

Though superior to bare-metal stents (BMS), drug-eluting stents (DES) based PCI still have significant in-stent restenosis (ISR). Balloon angioplasty (BA), drug-coated balloons (DCBs), and DES are common modalities to treat ISR. The existing guidelines recommend treating ISR with either DCB or DES for BMS-ISR and DES-ISR, despite differences in the underlying mechanisms. Because DES are currently the most used stents worldwide, we performed a network meta-analysis (NMA) to compare DES-ISR treatment strategies.

**Methods:**

We searched Cochrane Central Register of Controlled Trials, PubMed, Embase, and Scopus for relevant studies published until March 30, 2024 and performed a Bayesian NMA to synthesize direct and indirect evidence. The primary outcome was a target lesion revascularization (TLR) at follow-up.

**Results:**

Of 1202 studies, 30 were deemed eligible, with 15 being randomized studies. This included 8016 patients with DES-ISR who were assigned to 12 different PCI strategies. In the NMA for DES-ISR, paclitaxel-eluting stent (76.42) was the most effective strategy for TLR; paclitaxel-coated balloon (PCB) and scoring balloon angioplasty (75.88) for major adverse cardiovascular events (MACE); sirolimus-coated balloon (SCB) for target lesion failure (64.16), myocardial infarction (93.57), and stent thrombosis (98.53); and PCB for all-cause death (76.39) and cardiac death (83.74) based on SUCRA value. BA-based strategies were less effective alternatives for DES-ISR treatment with DCB or DES.

**Conclusions:**

DES and DCB PCI such as PCB and SCB should be considered for treatment of coronary DES-ISR to achieve the most clinical efficacy and safety benefits for MACE. Further studies are required for more robust evidence on different treatment strategies.

In 2019, ischemic heart disease was the leading cause of death worldwide according to the World Health Organization, accounting for >8.9 million deaths per year.^1^ Although advancements in health care over the last 2 to 3 decades have decreased this trend in European and North American countries,^2^^,^^3^ myocardial infarction (MI) and ischemic heart disease-related mortality remain a significant public health burden, with an annual incidence of 805,000 first and recurrent MI events in the United States.^4^ Decreases in MI-related mortality have been found to correspond with the increasing number of percutaneous coronary interventions (PCIs) performed each year, with 637,650 PCIs performed in 2017 across the United States, which could be due to the significant evolution in the intervention techniques and devices.^5^^,^^6^ Bare-metal stents (BMS) overcame the limitations of PCI with plain balloon angioplasty (POBA), but they carry the risk of vascular injury and in-stent restenosis (ISR) by triggering the fibroblast proliferation and the development of neointimal hyperplasia (NIH). These limitations of BMS were overcome significantly with the development of drug-eluting stents (DES), stents with antiproliferative drug coatings that suppress the formation of NIH and thus the risk of ISR. Even with these significant developments in PCI strategies and devices, ISR and the need for target lesion revascularization (TLR) still occur at a rate of 1% to 2% per year with DES platforms. Given the millions of DES coronary PCIs performed worldwide, ISR still can be considered a major public health issue.^6^^,^^7^ Over the past decade, PCI for ISR constituted about 10% of all PCIs performed in the United States.^6^

Approximately one-quarter of ISR cases present with MI, which has a 30-day mortality rate of 10% to 25%.^7^ PCI for ISR carries a higher risk of major adverse cardiac events (MACE) than PCI for de novo lesions.^6^ Thus, management of ISR remains challenging due to its heterogenous mechanisms and the relatively high rate of recurrence.^6^ Different PCI strategies have been used to manage ISR over the past 2 decades.^8^ Uniform recommendations regarding the specific treatment strategy for ISR are lacking.^6^, ^7^, ^8^ In the 2018 European Society of Cardiology/European Association for Cardio-Thoracic Surgery guideline, DES or drug-coated balloons (DCB) are recommended for the treatment of BMS-ISR or DES-ISR.^9^ In the 2021 American College of Cardiology/American Heart Association coronary revascularization guideline, repeated DES have a class 1A indication for treating ISR with PCI.^10^ There is no remark regarding the use of DCBs for coronary ISR treatment in the 2021 American College of Cardiology/American Heart Association guideline.^10^ However, in March 2024, the AGENT DCB received US Food and Drug Administration approval for ISR treatment^11^ on the basis of the findings of AGENT IDE trial,^12^ thus opening a new area for coronary ISR treatment in the United States.^13^^,^^14^

Although there have been more recent randomized and nonrandomized studies of DES-ISR patients, there is a lack of systematic review, meta-analysis, or network meta-analysis (NMA) to fully appraise the current situation on the different treatment strategies focusing on DES-ISR. Thus, we conducted this Bayesian NMA.

## Methods

### Study design

This network NMA was conducted in accordance with the NMA extension of PRISMA-P (Preferred Reporting Items for Systematic Review and Meta-Analysis Protocols).^15^ The literature search was performed using the PICO framework: population (patients with coronary DES-ISR), interventions (DCB including paclitaxel-coated balloon [PCB], sirolimus-coated balloon [SCB]; DES including everolimus-eluting stent [EES], paclitaxel-eluting stent [PES], sirolimus-eluting stent [SES], zotarolimus-eluting stent [ZES]; balloon angioplasty [BA] including percutaneous transluminal coronary angioplasty, scoring balloon angioplasty [SBA], cutting balloon angioplasty [CBA]), comparison (POBA), outcomes (MACE, target lesion failure [TLF], TLR, target vessel revascularization [TVR], MI, all-cause death, cardiac death, stent thrombosis [ST]). This study was registered in PROSPERO with registration number CRD42024499661.^16^

### Literature search and study selection

We performed an initial search of the Cochrane Central Register of Controlled Trials (CENTRAL), PubMed/Medline, Embase, and Scopus for relevant studies from database inception until January 30, 2024 using a search strategy with relevant keywords. We also searched for citation within each study to obtain more studies. The search was later updated on March 30, 2024 to look for any additional publications, after which the AGENT IDE trial data was added to the analysis. The detailed search strategy is described in Supplemental File S1.

For the NMA, all randomized clinical trials (RCTs), quasi-RCTs, and nonrandomized studies comparing different PCI strategies for the treatment of the coronary DES-ISR were eligible. ISR was defined as recurrent diameter stenosis at the stent segment of >50% of the vessel diameter and/or the 5-mm segment adjacent to the stent as detected by angiography.^7^^,^^8^ We did not restrict the search by date, publication status, or year of publication. The included studies (1) compared different PCI interventions between DES-ISR patients; (2) fulfilled good study criteria according to the GRADE Working Group^17^; and (3) reported the clinical efficacy and safety outcomes of interest, disregard the angiographic outcomes. Excluded studies were: (1) those conducted in nonhuman subjects; (2) had no data reporting on the clinical outcomes of interest; (3) discussed patients with left main disease; (4) described BMS-ISR >5% of the total; or (5) had no specific intervention mentioned, such as homo-DES vs hetero-DES groups, or different variants of same intervention, such as Shenqi PCB vs SeQuent Please PCB.

### Primary and secondary outcomes

The primary outcome was a TLR as a marker for the clinical efficacy. The secondary outcomes of interests were MACE, TLF, all-cause death, cardiac death, MI, and stent thrombosis (ST) (definite or probable). TLR was defined as any repeat percutaneous intervention of the target lesion or bypass surgery of the target vessel performed for restenosis or other complication of the target lesion. MACE was defined as the composite of death, MI, TVR, or TLR, which were slightly adjusted according to the specific situation. TLF was defined as the device-oriented composite outcome of cardiac death, MI attributed to the target vessel, and TLR. TVR was defined as any repeat percutaneous intervention or surgical bypass of any segment of the target vessel. The definitions of TLR, MACE, TLF, TVR, MI, ST, and all-cause death were in accordance with the Academic Research Consortium criteria.^18^

### Data collection

Covidence was used for data screening with 2 reviewers (F.K. and S.P.) screening the titles and abstracts of imported studies independently, with conflicts resolved by a third reviewer (P.O.). The full-text review was then performed independently by 2 reviewers (S.P. and P.O.). Conflicts were resolved by a third reviewer (D.S.).

A standardized electronic data form prepared in Microsoft Excel was used to extract the data on the study and patient baseline characteristics (Table 1),^12^^,^^19^, ^20^, ^21^, ^22^, ^23^, ^24^, ^25^, ^26^, ^27^, ^28^, ^29^, ^30^, ^31^, ^32^, ^33^, ^34^, ^35^, ^36^, ^37^, ^38^, ^39^, ^40^, ^41^, ^42^, ^43^, ^44^, ^45^, ^46^, ^47^ quantitative coronary angiography characteristics (Supplemental Table S1), and clinical outcomes (Table 2).^12^^,^^19^, ^20^, ^21^, ^22^, ^23^, ^24^, ^25^, ^26^, ^27^, ^28^, ^29^, ^30^, ^31^, ^32^, ^33^, ^34^, ^35^, ^36^, ^37^, ^38^, ^39^, ^40^, ^41^, ^42^, ^43^, ^44^, ^45^, ^46^, ^47^ Data from the included studies were extracted by 2 independent reviewers to reduce bias, and a third reviewer verified the data to avoid repeated inclusions. Any discrepancies in study inclusion or data collection were carefully examined and resolved by discussion with other review team members (D.S. and S.D.).

**Table 1:** Study characteristics and baseline characteristics of patients in the included studies

Study, year	Country	Study design	No. of patients	Intervention	Age, y	Male sex	BMI, kg/m^2^	Comorbidities						Smoking	Clinical diagnosis		
								Diabetes mellitus	Hypertension	Dyslipidemia	Previous MI	Prior CABG	Impaired renal function		Stable angina/silent ischemia	ACS	None or routine FU
**Randomized controlled trials**																	
RIBS IV trial,^19^ 2015	Spain	Prospective multicenter, open-label, controlled, randomized clinical trial	N = 309	PCB, n = 154	66 ± 10	127 (82)	**–**	75 (49)	110 (71)	110 (71)	73 (47)	16 (10)	**–**	89 (58)	74 (48)	80 (52)	**–**
				EES, n= 155	66 ± 10	130 (84)	**–**	66 (43)	121 (78)	121 (78)	77 (50)	17 (11)	**–**	87 (56)	76 (49)	79 (51)	**–**
Song et al,^20^ 2012	South Korea	Prospective, randomized, open-label, multicenter study	N = 162	Focal lesion, SES, n = 48	61.18 ± 8.79	35 (72.9)	**–**	18 (37.5)	28 (58.3)	25 (52.1)	8 (16.7)	**–**	**–**	16 (33.3)	32 (66.7)	16 (31.3)	**–**
				Focal lesion, CBA, n = 48	64.04 ± 9.71	36 (75.0)	**–**	15 (31.2)	32 (66.7)	25 (52.1)	2 (4.2)	**–**	**–**	23 (47.9)	37 (77.1)	11 (22.9)	**–**
				Diffuse lesion, SES, n = 32	60.61 ± 9.86	15 (46.9)	**–**	8 (25.0)	18 (56.3)	22 (66.8)	4 (12.5)	**–**	**–**	2 (6.3)	15 (46.8)	17 (53.1)	**–**
				Diffuse lesion, EES, n = 34	62.76 ± 11.07	19 (55.9)	**–**	12 (35.3)	19 (55.8)	30 (75.0)	3 (8.8)	**–**	**–**	6 (17.6)	23 (67.6)	11 (32.4)	**–**
Montorsi et al,^21^ 2004	Italy	Prospective, randomized study	N = 50	PTCA, n = 25	61 ± 10	19 (76)	**–**	**–**	**–**	**–**	**–**	**–**	**–**	**–**	19 (76)		6 (24)
				CBA, n = 25	66 ± 10	17 (68)	**–**	**–**	68 (94.4)	59 (81.9)	**–**	**–**	**–**	**–**	18 (72)		7 (28)
PEPCAD-DES trial,^22^	Germany	Prospective, single-blind, multicenter, randomized trial	N = 110	PCB, n = 72	69.8 ± 10.8	52 (72.2)	28.1 ± 4.1	26 (36.1)	36 (94.7)	27 (71.1)	**–**	**–**	**–**	11 (15.3)	69 (95.8)	3 (4.2)	**–**
				POBA, n = 38	64.0 ± 11.3	26 (68.4)	27.2 ± 3.5	13 (34.2)	68 (94.4)	59 (81.9)	**–**	**–**	**–**	6 (15.8)	37 (97.4)	1 (2.6)	**–**
PEPCAD China ISR trial,^23^ 2014		Randomized (1:1),single-blind, prospective, multicenter trial	N = 215	PCB, n = 109	61.8 ± 9.3	80.7 (88)	**–**	40.4 (44)	78 (71.6)	34.9 (38)	48.6 (53)	2.8 (3)	**–**	21.1 (23)	37 (34)	64.2 (70)	**–**
				PES, n = 106	62.1 ± 9.3	81.1 (86)	**–**	33.0 (35)	65.1 (69)	33.0 (35)	34.9 (37)	0	**–**	25.5 (27)	44 (41.5)	57.5 (61)	**–**
RESTORE trial,^24^ 2017	South Korea	Prospective, multicenter, open-label, randomized comparison trial	N = 172	PCB, n = 86	67 ± 10	61 (70.9)	**–**	43 (50.0)	60 (69.8)	49 (57.0)	26 (30.2)	**–**	**–**	40 (46.5)	36 (41.8)	44 (51.1)	**–**
				EES, n = 86	66 ± 9	62 (72.1)	**–**	38 (44.2)	65 (75.6)	53 (61.6)	22 (25.6)	**–**	**–**	37 (43.0)	39 (45.4)	40 (46.5)	**–**
ISAR-DESIRE 4 trial,^25^ 2017	Germany	Randomized, open-label, active controlled clinical trial	N = 252	DCB + SBA, n = 125	69.4 ± 9.5	100 (80)	**–**	51 (40.8)	71 (56.8)	110 (88.0)	56 (44.8)	18 (14.4)	**–**	19 (15.2)	**–**	31 (24.8)	**–**
				DCB, n = 127	69.4 ± 10.4	111 (87.4)	**–**	55 (43.3)	81 (63.8)	105 (82.7)	68 (53.5)	17 (13.4)	**–**	23 (18.1)	**–**	29 (22.8)	**–**
ISAR-DESIRE 3 trial,^26^ 2012	Germany	Randomized, open-label trial	N = 402	PCB, n = 137	67.7 ± 10.4	105 (89.6)	**–**	56 (41)	105 (77)	108 (79)	53 (39)	15 (11)	**–**	19 (14)	**–**	26 (19)	**–**
				PES, n = 131	68.8 ± 10.0	88 (67)	**–**	61 (41)	101 (77)	103 (79)	50 (38)	32 (24)	**–**	15 (11)	**–**	22 (17)	**–**
				POBA, n = 134	67.1 ± 9.3	95 (71)	**–**	50 (37)	90 (67)	102 (76)	57 (43)	24 (18)	**–**	22 (16)	**–**	31 (23)	**–**
ISAR-DESIRE 2 trial,^27^ 2010	Germany	Randomized, open-label, active controlled trial	N = 450	SES, n = 225	66.4 ± 10.9	178 (79.2)	**–**	86 (38.2)	163 (72.4)	150 (66.)	102 (45.3)	38 (16.9)	**–**	26 (11.6)	180 (80.0)	45 (20.0)	**–**
				PES, n = 225	67.1 ± 10.4	167 (74.3)	**–**	76 (33.8)	163 (72.4)	146 (64.9)	100 (44.4)	43 (19.1)	**–**	28 (12.4)	187 (83.1)	38 (16.9)	**–**
RESTENT-ISR trial,^28^ 2016	South Korea	Prospective, randomized, single blinded, investigator-initiated trial	N= 304	EES, n = 158	64.1 ± 8.9	106 (67.1)	25.1 ± 3.4	58 (36.7)	92 (58.2)	106 (67.1)	39 (24.7)	**–**	**–**	24 (15.2)	77 (49.4)	80 (50.6)	**–**
				ZES, n = 146	62.2 ± 10.2	52 (32.9)	24.7 ± 2.9	53 (36.3)	77 (52.7)	103 (70.5)	46 (31.5)	**–**	**–**	34 (23.3)	69 (47.3)	77 (52.7)	**–**
Habara et al,^29^ 2011	Japan	Prospective single-blind randomized trial	N = 50	PCB, n = 25	69.9 ± 11.0	19 (76)	**–**	14 (56)	15 (60)	15 (60)	8 (32)	**–**	**–**	1 (4)	**–**	**–**	**–**
				POBA, n = 25	68.9 ± 9.9	24 (96)	**–**	17 (68)	17 (68)	16 (64)	14 (56)	**–**	**–**	0	**–**	**–**	**–**
CRISTAL trial,^30^ 2012	France	Prospective randomized trial	N = 197	SES, n = 136	68 ± 10	98 (72)	**–**	53 (39)	99 (3)	105 (77)	34 (25)	14 (10)	**–**	86 (63)	**–**	**–**	**–**
				POBA, n= 61	67 ± 11	42 (69)	**–**	23 (38)	46 (75)	48 (79)	14 (23)	5 (8)	**–**	31 (51)	**–**	**–**	**–**
Ali et al,^31^ 2019	Malaysia	Randomized, prospective, controlled multicenter trial	N = 50	DCB, n = 25	58.6 ± 12.5	**–**	**–**	19 (76)	23 (92)	21 (84)	9 (36)	4 (16)	**–**	2 (8)	**–**	15 (60)	**–**
				SCB, n= 25	61.6 ± 11.7	**–**	**–**	18 (72)	24 (96)	23 (92)	8 (32)	1 (4)	**–**	4 (16)	**–**	13 (52)	**–**
AGENT IDE trial,^12^ 2024	USA	Multicenter, single-blind, randomized controlled, superiority trial	N = 600	PCB, n = 406	68.4 ± 9.8	302 (74.4)	30.0 ± 5.5	206 (51.0)	383 (94.6)	382 (94.6)	198 (49.7)	124 (30.8)	74 (18.4)	42 (10.3)	232 (57.1)	149 (36.7)	**–**
				POBA, n = 194	67.9 ± 9.7	141 (72.7)	30.1 ± 5.9	97 (50.0)	186 (95.9)	184 (94.8)	95 (50.0)	55 (28.6)	32 (16.6)	19 (9.8)	105 (54.1)	73 (37.6)	**–**
Scheller B et al,^32^ 2022	Malayasia, Europe	Parallel conducted randomized, prospective, controlled multicenter trial	N = 101	SCB, n = 50	67 ± 12	43 (86)	**–**	27 (54)	49 (98)	44 (88)	28 (56)	2 (4)	**–**	11 (22)	26 (52)	19 (38)	5 (20)
				PCB, n = 51	63 ± 12	39 (76)	**–**	30 (59)	48 (94)	43 (84)	28 (55)	5 (10)	**–**	6 (12)	30 (59)	17 (33)	4 (8)
**Nonrandomized studies**																	
Wańha et al,^33^ 2021	Poland	Large multicenter observational study	N = 1117	DCB, n = 557	66.8 ± 9.8	404 (72.5)	28.7 ± 4.4	265 (47.6)	507 (91.0)	472 (84.7)	347 (62.3)	131 (23.5)	151 (27.1)	113 (20.3)	**–**	**–**	**–**
				DES, n = 560	66.8 ± 10.6	381 (68.0)	28.6 ± 4.0	208 (37.1)	487 (87.0)	485 (86.6)	352 (62.9)	79 (14.1)	104 (18.6)	110 (19.6)	**–**	**–**	**–**
Nojima et al,^34^ 2014	Japan	Retrospective observational study	N = 191	POBA, n = 38	72.0 ± 6.9	26 (68)	**–**	23 (61)	31 (82)	30 (79)	16 (42)	3 (8)	**–**	**–**	**–**	**–**	**–**
				Homo-DES, n = 38	71.0 ± 7.6	26 (68)	**–**	24 (63)	27 (71)	27 (72)	16 (42)	2 (1)	**–**	**–**	**–**	**–**	**–**
				Hetero-DES, n = 115	69.7 ± 7.5	80 (70)	**–**	71 (62)	84 (73)	81 (70)	53 (46)	1 (1)	**–**	**–**	**–**	**–**	**–**
Zhang et al,^35^ 2022	China	Retrospective observational study	N = 214	DCB, n = 78	57.8 ± 9.0	62 (79.5)	26.5 ± 3.2	33 (42.3)	50 (64.1)	60 (76.9)	35 (44.9)	3 (3.8)	10 (12.8)	29 (37.2)	**–**	**–**	**–**
				DES, n = 136	60.0 ± 8.8	117 (86.0)	26.3 ± 2.8	61 (44.9)	84 (61.8)	103 (75.7)	78 (57.4)	7 (5.1)	9 (6.6)	39 (28.7)	**–**	**–**	**–**
Kang et al,^36^ 2015	South Korea	Retrospective observational study	N = 238	DCB, n = 182	63.1 ± 9.8	125 (68.7)	**–**	80 (44.0)	132 (72.5)	165 (90.7)	**–**	**–**	**–**	85 (46.7)	**–**	60 (33.0)	**–**
				DES, n = 56	59.5 ± 11.0	36 (64.3)	**–**	16 (28.6)	39 (69.6)	46 (82.1)	**–**	**–**	**–**	26 (46.4)	**–**	24 (42.9)	**–**
Habara et al,^37^ 2016	Japan	Retrospective observational study	N = 685	PCB, n = 260	69.2 ± 9.9	195 (75.0)	**–**	134 (51.5)	203 (78.1)	159 (61.2)	133 (51.2)	23 (8.9)	**–**	10 (3.9)	238 (91.5)	22 (8.5)	**–**
				DES, n = 425	68.6 ± 10.9	331 (77.9)	**–**	231 (54.4)	318 (74.8)	258 (60.7)	220 (51.8)	27 (6.4)	**–**	20 (4.7)	363 (85.4)	62 (14.6)	**–**
Wang et al,^38^ 2019	China	Retrospective observational study	N = 172	DES, n = 79	66.5 ± 11.2	51 ((64.6)	**–**	26 (32.9)	49 (62.0)	50 (63.3)	9 (11.4)	3 (3.8)	10 (12.7)	25 (31.6)	**–**	26 (33.1)	**–**
				DCB, n = 93	67.3 ± 13.4	61 (65.6)	**–**	31 (33.3)	56 (60.2)	59 (63.4)	12 (12.9)	4 (4.3)	12 (12.9)	32 (34.4)	**–**	28 (30.1)	**–**
Basavarajaiah et al,^39^ 2015	Italy	Retrospective observational study	N = 247	DCB, n = 81	66.8 ± 9.0	73 (90.1)	**–**	38 (46.9)	57 (70.4)	59 (72.8)	30 (37.0)	25 (30.9)	**–**	7 (8.6)	**–**	**–**	**–**
				DES, n = 166	65.7 ± 9.6	143 (86.1)	**–**	55 (33.1)	118 (71.1)	127 (76.5)	85 (51.2)	56 (33.7)	**–**	12 (7.2)	**–**	**–**	**–**
Almalla et al,^40^ 2014	Germany	Single-center, retrospective observational study	N = 86	EES, n = 40	67.7 ± 10.8	28 (70)	**–**	14 (35)	34 (85)	**–**	21 (52.5)	4 (10)	5 (12.5)	21 (52.5)	**–**	**–**	**–**
				DCB, n = 46	69.6 ± 9.6	38 (82)	**–**	18 (39.1)	37 (80.4)	**–**	17 (36.9)	10 (21.7)	12 (26)	14 (30.4)	**–**	**–**	**–**
Wolny et al,^41^ 2022	Poland	Multicenter observational registry study	N = 311	DCB, n = 225	67.7 ± 9.9	151 (67.1)	28.5 ± 4.6	110 (48.9)	201 (89.3)	184 (81.8)	169 (75.1)	67 (29.8)	70 (31.1)	43 (19.1)	74 (32.9)	151 (67.1)	**–**
				DES, n = 86	65.3 ± 10.0	57 (66.3)	27.6 ± 3.8	34 (39.5)	83 (96.5)	71 (82.6)	64 (74.4)	21 (24.4)	15 (17.1)	26 (30.2)	20 (23.3)	66 (76.6)	**–**
Kim et al,^42^ 2009	South Korea	Multicenter observational study	N = 48	POBA, n = 19	**–**	**–**	**–**	**–**	**–**	**–**	**–**	**–**	**–**	**–**	**–**	**–**	**–**
				CBA, n = 15	**–**	**–**	**–**	**–**	**–**	**–**	**–**	**–**	**–**	**–**	**–**	**–**	**–**
				DES, n = 14	**–**	**–**	**–**	**–**	**–**	**–**	**–**	**–**	**–**	**–**	**–**	**–**	**–**
Marquis-Gravel et al,^43^ 2017	Canada	Multicenter observational study	N = 180	DCB, n = 91	65.30 ± 9.04	70 (77)	29.41 ± 6.03	43 (47)	80 (89)	86 (97)	**–**	26 (29)	22 (28)	**–**	26 (29)	65 (71)	**–**
				DES, n = 89	65.30 ± 13.56	65 (73)	27 ± 4.52	33 (39)	72 (84)	81 (93)	**–**	17 (20)	26 (33)	**–**	20 (23)	69 (77)	**–**
Kawamoto et al,^44^ 2015	Italy	Retrospective observational study	N = 133	DES, n = 68	64.9 ± 9.1	63 (92.6)	**–**	28 (41.2)	54 (79.4)	54 (79.4)	42 (61.8)	27 (39.7)	39 (58.2)	9 (13.2)	**–**	10 (14.7)	**–**
				DCB, n = 65	67.2 ± 8.9	57 (87.7)	**–**	28 (43.1)	51 (78.5)	51 (78.5)	36 (55.4)	17 (26.2)	40 (61.5)	6 (9.2)	**–**	12 (18.5)	**–**
Kook et al,^45^ 2020	South Korea	Single-center retrospective study	N= 75	DES, n = 51	64.3 ± 10.9	41 (80.4)	**–**	23 (45.1)	34 (66.7)	**–**	16 (31.4)	**–**	**–**	16 (31.4)	38 (74.5)	13 (25.5)	**–**
				DCB, n = 24	64.8 ± 11.3	17 (70.8)	**–**	9 (37.5)	14 (58.3)	**–**	6 (25)	**–**	**–**	6 (25)	22 (91.7)	2 (8.3)	**–**
Abdelmegid et al,^46^ 2017	Japan	Single-center retrospective observational study	N = 90	POBA, n = 66	70.9 ± 9.8	48 (77.4)	**–**	40 (64.5)	57 (91.9)	42 (67.7)	29 (46.8)	4 (6.5)	22 (35.5)	13 (21)	**–**	**–**	**–**
				DES, n = 24	68.1 ± 5.8	18 (81.8)	**–**	13 (59.1)	20 (90.9)	16 (72.7)	10 (45.5)	1 (4.5)	8 (36.4)	2 (9.1)	**–**	**–**	**–**
Ko et al,^47^ 2012	South Korea	Retrospective, multicenter registry study	N = 805	DES, n = 422	62.0 ± 10.8	282 (66.8)	**–**	168 (39.8)	259 (61.4)	168 (39.8)	**–**	3 (0.7)	30 (7.1)	100 (23.7)	**–**	125 (29.6)	**–**
				POBA + CBA, n = 383	62.8 ± 9.6	269 (70.2)	**–**	151 (39.4)	215 (56.1)	134 (35)	**–**	7 (1.8)	28 (7.3)	85 (22.2)	**–**	83 (21.7)	**–**

Values are mean ± SD or n (%).

ACS, acute coronary syndrome; BMI, body mass index; CABG, coronary artery bypass graft; CBA, cutting balloon angioplasty; DCB, drug-coated balloon; DES, drug-eluting stent; EES, everolimus-eluting stent; FU, follow-up; MI, myocardial infarction; PCB, paclitaxel-coated balloon; PES, paclitaxel-eluting stent; POBA, plain balloon angioplasty; PTCA, percutaneous transluminal coronary angioplasty; SBA, scoring balloon angioplasty; SCB, sirolimus-coated balloon; SES, sirolimus-eluting stent; ZES, zotarolimus-eluting stent.

**Table 2:** Primary and secondary clinical outcomes among included patients.

Study, year	Intervention	Primary outcome	Secondary outcomes, annualized event rate							Follow-up duration, y
		TLR	MACE	TLF	TVR	MI	All-cause death	Cardiac death	Stent thrombosis	
**Randomized controlled trials**										
RIBS IV trial,^19^ 2015	DCB, n = 154	0.17	0.24	**–**	0.21	0.04	0.03	0.02	**–**	0.76
	EES, n = 155	0.06	0.14	**–**	0.11	0.02	0.03	0.02	**–**	0.76
Song et al,^20^ 2012	SES, n = 80	1	6	**–**	1	0.050	**–**	**–**	0.013	1
	EES, n = 34	0.059	0.088	**–**	0.059	0.029	**–**	**–**	0.000	1
	CBA, n = 48	0.063	0.063	**–**	0.063	0.000	**–**	**–**	0.000	1
Montorsi et al,^21^ 2004	PTCA, n = 25	0.80	0.56	**–**	**–**	**–**	**–**	**–**	**–**	0.5
	CBA, n = 25	0.25	0.33	**–**	**–**	**–**	**–**	**–**	**–**	0.5
PEPCAD-DES trial,^22^ 2012	PCB, n = 72	0.31	0.33	**–**	**–**	0.000	**–**	0.028	0.028	0.5
	POBA, n = 38	0.74	1.00	**–**	**–**	0.053	**–**	0.21	0.210	0.5
PEPCAD China ISR trial,^23^ 2014	PCB, n= 109	0.16	0.24	0.13	0.17	0.038	0.000	**–**	0.009	1
	PES, n = 106	0.12	0.24	0.19	0.16	0.066	0.019	**–**	0.009	1
RESTORE trial,^24^ 2017	PCB, n = 86	0.058	0.070	**–**	0.058	0.012	**–**	**–**	**–**	1
	EES, n = 86	0.012	0.047	**–**	0.012	0.035	**–**	**–**	**–**	1
ISAR-DESIRE 4 trial,^25^ 2017	DCB + SBA, n = 125	0.16	0.18	**–**	**–**	**–**	0.016	**–**	**–**	1
	DCB, n = 127	0.21	0.23	**–**	**–**	**–**	0.016	**–**	**–**	1
ISAR-DESIRE 3 trial,^26^ 2012	PCB, n = 137	0.33	0.35	**–**	**–**	**–**	**–**	**–**	**–**	0.67
	PES, n = 131	0.19	0.28	**–**	**–**	**–**	**–**	**–**	**–**	0.67
	POBA, n = 134	0.62	0.68	**–**	**–**	**–**	**–**	**–**	**–**	0.67
ISAR-DESIRE 2 trial,^27^ 2010	SES, n = 225	0.16	0.20	**–**	0.22	0.004	0.031	**–**	**–**	1
	PES, n =225	0.13	0.18	**–**	0.21	0.004	0.040	**–**	**–**	1
RESTENT-ISR trial,^28^ 2016	EES, n = 158	0.049	0.053	**–**	**–**	0.006	0.002	**–**	0.008	3
	ZES, n = 146	0.071	0.075	**–**	**–**	0.009	0.007	**–**	0.005	3
Habara et al,^29^ 2011	PCB, n = 25	0.08	0.08	**–**	**–**	**–**	**–**	**–**	**–**	0.5
	POBA, n = 25	0.80	0.80	**–**	**–**	**–**	**–**	**–**	**–**	0.5
CRISTAL trial,^30^ 2012	SES, n = 136	0.059	**–**	**–**	0.022	0.007	0.029	0.007	**–**	1
	POBA, n = 61	0.13	**–**	**–**	0.00	0.000	0.033	0.06	**–**	1
Ali et al,^31^ 2019	DCB, n = 25	0.16	0.16	0.078	**–**	**–**	**–**	**–**	0.004	1
	SCB, n = 25	0.12	0.12	0.073	**–**	**–**	**–**	**–**	0.000	1
AGENT IDE trial,^12^ 2024	PCB, n = 406	**–**	**–**	0.096	**–**	**–**	**–**	**–**	**–**	**1**
	POBA, n = 194	**–**	**–**	0.17	**–**	**–**	**–**	**–**	**–**	**1**
Scheller et al,^32^ 2022	SCB, n = 50	0.15	0.17	**–**	**–**	0.000	0.019	**–**	0.000	1.08
	PCB, n = 51	0.091	0.13	**–**	**–**	0.018	0.018	**–**	0.018	1.08
**Nonrandomized studies**										
Wańha et al,^33^ 2021	DCB, n =557	0.046	**–**	0.056	0.056	0.038	**–**	0.007	**–**	3
	DES, n = 560	0.030	**–**	0.073	0.030	0.035	**–**	0.012	**–**	3
Nojima et al,^34^ 2014	POBA, n = 38	0.089	0.10	**–**	**–**	**–**	**–**	**–**	0.006	5
	DES, n = 147	0.034	0.072	**–**	**–**	**–**	**–**	**–**	0.004	5
Zhang et al,^35^ 2022	DCB, n = 78	0.071	0.092	**–**	**–**	0.046	**–**	0.008	**–**	3.18
	DES, n = 136	0.067	0.089	**–**	**–**	0.040	**–**	0.015	**–**	3.18
Kang et al,^36^ 2015	DCB, n = 182	0.049	0.055	**–**	**–**	0.003	**–**	0.016	**–**	2
	DES, n = 56	0.045	0.045	**–**	**–**	0.000	**–**	0.000	**–**	2
Habara et al,^37^ 2016	PCB, n =260	0.16	0.15	**–**	0.15	0.000	**–**	0.000	0.000	1
	DES, n = 425	0.20	0.20	**–**	0.20	0.004	**–**	0.013	0.004	1
Wang et al,^38^ 2019	DES, n = 79	0.12	0.14	**–**	**–**	0.051	0.009	**–**	**–**	1.26
	DCB, n = 93	0.23	0.26	**–**	**–**	0.090	0.020	**–**	**–**	1.26
Basavarajaiah et al,^39^ 2015	DCB, n = 81	0.11	0.067	**–**	0.13	0.000	**–**	0.013	**–**	1.83
	DES, n = 166	0.086	0.046	**–**	0.099	0.003	**–**	0.000	**–**	1.83
Almalla et al,^40^ 2014	EES, n = 40	0.026	0.051	**–**	0.038	0.013	0.026	**–**	0.000	1.96
	DCB, n = 46	1.0	0.12		1.00	0.022	0.011	**–**	0.011	1.96
Wolny et al,^41^ 2022	DCB, n = 225	0.10	**–**	70	0.12	0.080	0.019	0.014	**–**	2.6
	DES, n = 86	0.081	**–**	24	0.11	0.081	0.045	0.031	**–**	2.6
Kim et al,^42^ 2009	POBA, n = 19	**–**	0.57	**–**	**–**	**–**	**–**	**–**	**–**	0.83
	CBA, n = 15	**–**	0.56	**–**	**–**	**–**	**–**	**–**	**–**	0.83
	DES, n = 14	**–**	0.26	**–**	**–**	**–**	**–**	**–**	**–**	0.83
Marquis-Gravel et al,^43^ 2017	DCB, n = 91	0.049	0.18	**–**	**–**	0.044	0.12	**–**	0.005	2
	DES, n = 89	0.039	0.095	**–**	**–**	0.028	0.034	**–**	0.000	2
Kawamoto et al,^44^ 2015	DES, n = 68	0.044	0.052	**–**	**–**	0.000	0.007	**–**	**–**	2.08
	DCB, n = 65	0.057	0.064	**–**	**–**	0.007	0.007	**–**	**–**	2.08
Kook et al,^45^ 2020	DES, n = 51	0.070	0.11	**–**	**–**	0.000	0.000	0.000	0.000	2.38
	DCB, n = 24	0.091	0.11	**–**	**–**	0.008	0.016	0.008	0.016	2.38
Abdelmegid et al,^46^ 2017	POBA, n = 66	0.19	**–**	**–**	**–**	**–**	**–**	**–**	**–**	2
	DES, n = 24	0.083	**–**	**–**	**–**	**–**	**–**	**–**	**–**	2
Ko et al,^47^ 2012	DES, n = 422	0.076	0.11	**–**	0.076	0.045	0.052	**–**	0.021	1
	POBA + CBA, n = 383	0.18	0.21	**–**	0.19	0.044	0.065	**–**	0.026	1

CBA, cutting balloon angioplasty; DCB, drug-coated balloon; DES, drug-eluting stent; EES, everolimus-eluting stent; MACE, major adverse cardiovascular event; MI, myocardial infarction; PCB, paclitaxel-coated balloon; PES, paclitaxel-eluting stent; POBA, plain balloon angioplasty; PTCA, percutaneous transluminal coronary angioplasty; SBA, scoring balloon angioplasty; SES, sirolimus-eluting stent; TLF, target lesion failure; TLR, target lesion revascularization; ZES, zotarolimus-eluting stent.

### Quality assessment: risk of bias and grading the certainty of evidence

Randomized studies were assessed for bias using the Cochrane risk of bias tool^48^ whereas the quality of nonrandomized studies was assessed using the ROBINS-I tools for nonrandomized studies.^49^ RevMan 5.4^50^ was used to create the graph and summary of the included studies (Supplemental Figure S1).

We used the CINeMA (Confidence in Network Meta-Analysis) web application^51^^,^^52^ to examine the confidence in the findings from the NMA for the primary outcome, TLR, as shown in Supplemental Figure S2. CINeMA considers 6 domains: (1) within-study bias, (2) reporting bias (referring to publication and other reporting bias), (3) indirectness, (4) imprecision, (5) heterogeneity, and (6) incoherence.

### Network meta-analysis

For each outcome, we conducted a Bayesian NMA by fitting a generalized linear model with a complementary logit link function and binomial likelihood function using the “BUGSnet” (Bayesian inference Using Gibbs Sampling to conduct a Network meta-analysis) package in R^53^ and RStudio (version 2023.12.1, build 402).^54^ We performed Bayesian analysis with Markov Chain Monte Carlo (MCMC) simulation by conducting 4 MCMC chains simultaneously. The random-effect model was chosen for the outcomes of interest while running the MCMC model to account the variances within each study and between the studies. We specified a burn-in of 50,000 iterations followed by 100,000 iterations with 10,000 adaptations, consistent with the National Institute for Health and Clinical Excellence Decision Support Unit technical support document.^55^ Network diagrams were generated for each outcome wherein the node size and line width represent the number of patients and the number of trials for different comparisons, respectively.

Posterior medians of odds ratios (ORs) and 95% credible intervals (CrIs) were used to express the effect size as shown in the Central Illustration. NMA forest and league plots were used to depict the network estimates of different comparisons. We estimated the probabilities of each treatment being at each rank for each intervention and outcome. To rank the treatments for each outcome, we used the surface under the cumulative ranking curve (SUCRA) and the mean ranks; a larger SUCRA value implied a higher hierarchy.^56^ League table heatmaps were created to determine the relative odds ratios among comparisons.

**Central Illustration:**
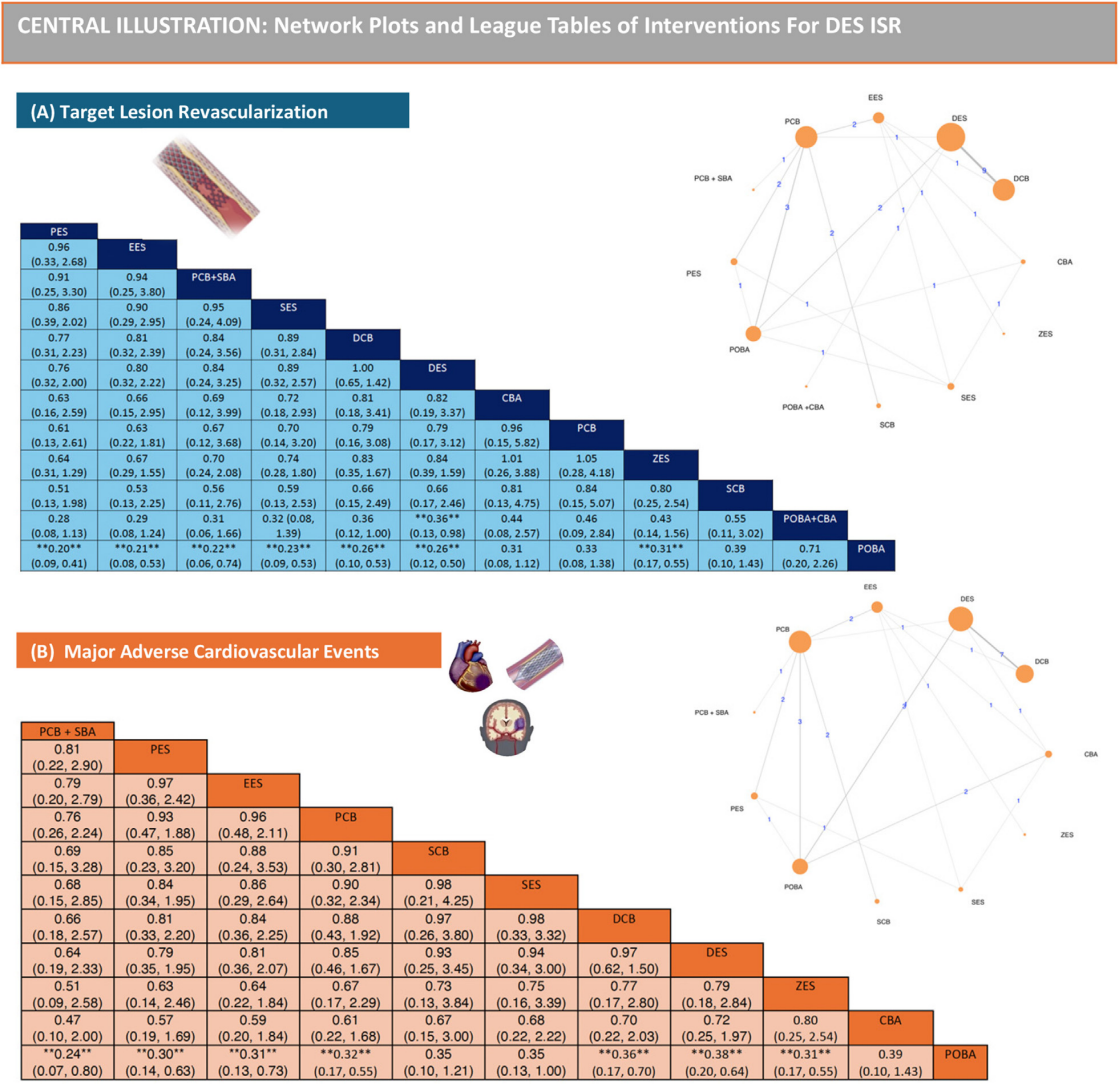
Comparative clinical efficacy and safety of different percutaneous coronary intervention strategies for coronary DES-ISR: a systematic review and Bayesian network meta-analysis of randomized and nonrandomized studies. Network plots and league tables of interventions for DES-ISR: (**A**) target lesion revascularization; (**B**) major adverse cardiac events. CBA, cutting balloon angioplasty; DCB, drug-coated balloon; DES, drug-eluting stent; EES, everolimus-eluting stent; ISR, in-stent restenosis; PCB, paclitaxel-coated balloon; PES, paclitaxel-eluting stent; POBA, plain balloon angioplasty; SBA, scoring balloon angioplasty; SCB, sirolimus-coated balloon; SES, sirolimus-eluting stent; ZES, zotarolimus-eluting stent.

Inconsistency refers to the extent of disagreement between direct and indirect evidence. To assess the presence of inconsistency, we fitted an NMA model like the one previously described for inconsistency model. We obtained leverage plots for the consistency and inconsistency models. To evaluate any inconsistency between the direct and indirect evidence, we compared the deviance information criterion (DIC) value and visual examinations of the degree of heterogeneity in the leverage plots for consistency and inconsistency models. The same or similar DIC values (<5 point difference) and <3 point difference in the visual inspection were used to indicate the consistency between the direct and indirect evidence. We also plotted the posterior mean deviation contributions for each data point to assess the inconsistency.

Two different models were run for each outcome: a random-effect consistency model and a random-effect inconsistency model. The DIC value provided a measure of model fit that reduced the complexity of the model; a lower DIC value indicated a better model fit. The potential scale reduced factor (PSRF) was used to judge the convergence of the models. When the PSRF value was between 1.00 and 1.05, the convergence of the iteration effect was good; otherwise, iterative calculations with larger parameters were used until the PSRF value was between 1.00 and 1.05.

To examine the homogeneity assumption for the direct evidence, we obtained Higgins & Thompson’s *I*^*2*^ statistic through pairwise comparison of intervention pairs that were reported in ≥2 included studies. *I*^*2*^ values of ≤25%, ≤50%, and ≥75% were defined as low, medium, and high heteroge respectively.

We utilized combined data from the randomized and nonrandomized studies reporting the outcome of interest for the primary analysis. However, there is a potential risk of bias from combining high-quality data from RCTs with the low to moderate quality data from nonrandomized studies.^57^ We utilized a risk bias tool for the RCTs and non-RCTs to ensure similar quality levels among them. In addition, we performed subanalysis of data from RCTs only to check whether there was any significant difference in findings between the primary combined data analysis and subanalysis for TLR and MACE (primary outcome and most often reported secondary outcome).

## Results

From the initial database search, 1202 studies were identified, among which 871 studies underwent title and abstract review after 331 duplicates were removed. After the title and abstract screening, only 112 studies were selected for full-text review, which led to 30 studies being chosen for data extraction. Among these 30 studies, 15 were RCTs and the remaining 15 studies were nonrandomized studies. The PRISMA flow diagram for the review is shown in Supplemental Figure S3.

### Qualitative summary

**Figure 1:**
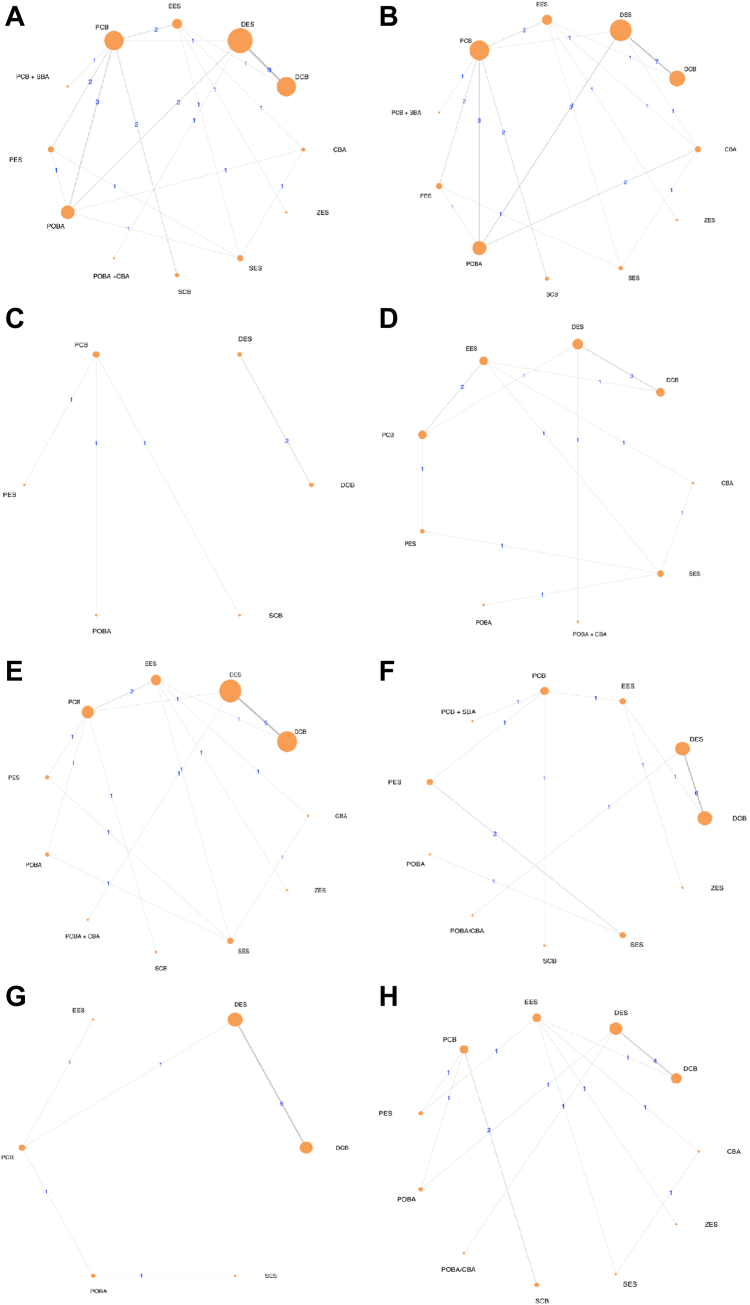
**Network plot depicting thedifferent PCI interventionsincluded for different outcomes.** (A) TLR; (B) MACE; (C) TLF; (D) TVR; (E) MI; (F) all-cause death; (G) cardiac death; (H) stent thrombosis. CBA, cutting balloon angioplasty; DCB, drug-coated balloon; DES, drug-eluting stent; EES, everolimus-eluting stent; MACE, major adverse cardiovascular event; MI, myocardial infarction; PCB, paclitaxel-coated balloon; PES, paclitaxel-eluting stent; POBA, plain balloon angioplasty; SBA, scoring balloon angioplasty; SCB, sirolimus-coated balloon; SES, sirolimus-eluting stent; TLF, target lesion failure; TLR, target lesion revascularization; TVR, target vessel revascularization; ZES, zotarolimus-eluting stent.

Thirty studies with 8016 patients with DES-ISR were included for the analysis in this Bayesian NMA. Among 30 included studies, 15 studies were randomized controlled trials^12^^,^^19^, ^20^, ^21^, ^22^, ^23^, ^24^, ^25^, ^26^, ^27^, ^28^, ^29^, ^30^, ^31^, ^32^ involving 3424 (42.71%) DES-ISR patients and 15 studies were nonrandomized observational studies^33^, ^34^, ^35^, ^36^, ^37^, ^38^, ^39^, ^40^, ^41^, ^42^, ^43^, ^44^, ^45^, ^46^, ^47^ involving 4592 (57.29%) DES-ISR patients. Among 7918 patients, 5648 (71.33%) patients were male, and 2270 (28.67%) patients were female. The mean age of the included patients was 66.06 ± 10.44 years. The included patients were assigned to 12 different PCI strategies for DES-ISR: DCB (38.6% of total) including PCB (18.11% of total), SCB (0.94% of total), PCB + SBA (1.56% of total); DES (48.04% of total) including SES (5.50% of total), EES (5.90% of total), ZES (1.82% of total), PES (5.76% of total); BA (13.36% of total) including POBA (7.49% of total), CBA (1.10% of total), and POBA + CBA (4.78% of total). A comparison of these treatment modalities is shown in a network map (Figure 1).

### Quantitative summary

#### Convergence of models

TLR (Supplemental Figure S4A), MACE (Supplemental Figure S4C), TLF (Supplemental Figure S4E), TVR (Supplemental Figure S4F), MI (Supplemental Figure S4G), all-cause death (Supplemental Figure S4H), cardiac death (Supplemental Figure S4I), and ST (Supplemental Figure S4J) showed good convergence.

In subanalysis involving the RCTs only, TLR (Supplemental Figure S4B) and MACE (Supplemental Figure S4D) also showed good convergence of the selected models.

#### TLR

Twenty-eight studies reported the TLR events and assigned 7140 patients with DES-ISR to 12 interventions. Of the 66 possible pairwise comparisons, only 18 had direct comparison data. Of the 28 studies, 26 were 2-arm and 2 were multiarm. There were 1086 TLR events, with none of the studies having zero events in any arm. The network plot was connected as shown in Figure 1A.

**Figure 2:**
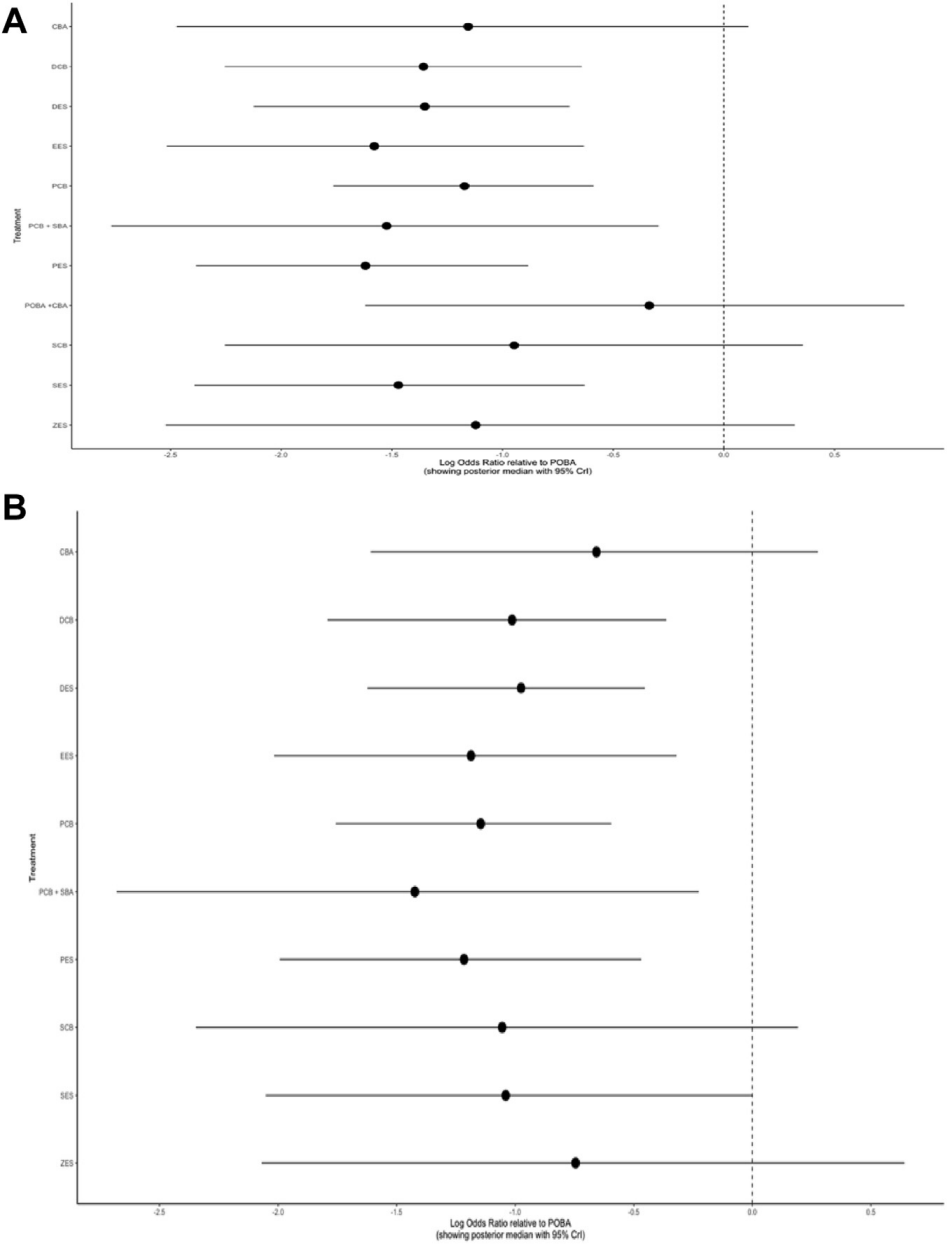
**NMA forest plot displaying effect estimates of different interventions compared with POBA.** (A) TLR; (B) MACE. CBA, cutting balloon angioplasty; CrI, credible interval; DCB, drug-coated balloon; DES, drug-eluting stent; EES, everolimus-eluting stent; MACE, major adverse cardiovascular event; NMA, network meta-analysis; PCB, paclitaxel-coated balloon; PES, paclitaxel-eluting stent; POBA, plain balloon angioplasty; SBA, scoring balloon angioplasty; SCB, sirolimus-coated balloon; SES, sirolimus-eluting stent; TLR, target lesion revascularization; ZES, zotarolimus-eluting stent.

In the NMA forest plot, compared with POBA, DCB (odds ratio [OR], −1.36; 95% CrI, −2.25 to −0.64), DES (OR, −1.35; 95% CrI, −2.12 to −0.70), EES (OR, −1.58; 95% CrI, −2.52 to −0.63), PCB (OR, −1.17; 95% CrI, −1.76 to −0.59), PCB + SBA (OR, −1.52; 95% CrI, −2.77 to −0.30), PES (OR: −1.62; 95% CrI, −2.38 to −0.88), and SES (OR: −1.47; 95% CrI, −2.39 to −0.63) might significantly lower occurrence of the TLR events but not with the other interventions using the random-effect model, as shown in Figure 2A.

**Figure 3:**
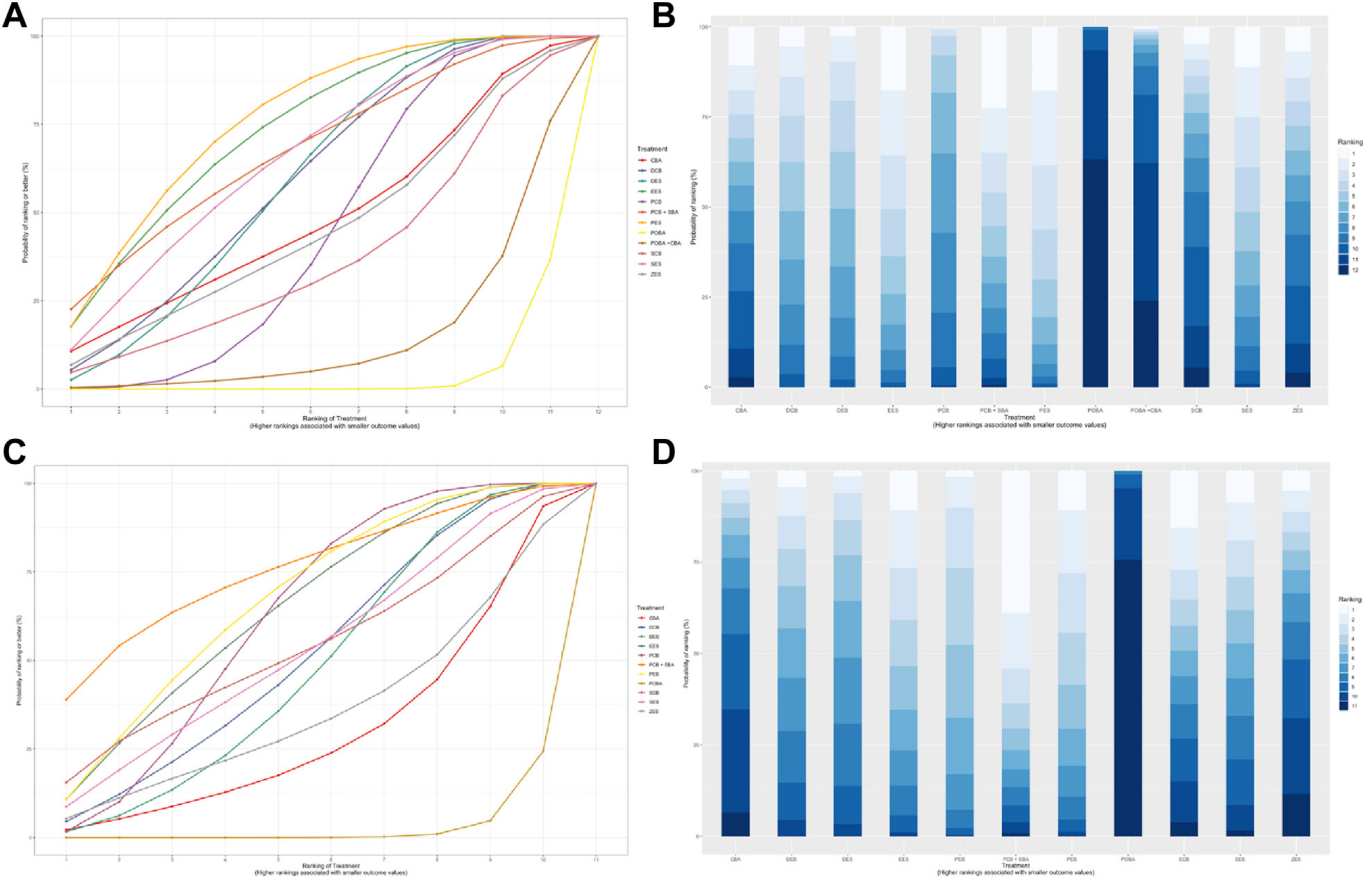
**Cumulative probability ranking chart and ranking probability histogram for MACE.** The SUCRA and rankogram charts intuitively display the sorting probability of each intervention group in the form of curves and histogram. For TLR, (A) SUCRA chart and (B) Rankogram chart. For MACE, (C) SUCRA chart and (D) rankogram chart. CBA, cutting balloon angioplasty; DCB, drug-coated balloon; DES, drug-eluting stent; EES, everolimus-eluting stent; MACE, major adverse cardiovascular event; PCB, paclitaxel-coated balloon; PES, paclitaxel-eluting stent; POBA, plain balloon angioplasty; SBA, scoring balloon angioplasty; SCB, sirolimus-coated balloon; SES, sirolimus-eluting stent; SUCRA, surface under the cumulative ranking curve; TLR, target lesion revascularization; ZES, zotarolimus-eluting stent.

SUCRA values ranked PES (76.42) highest and POBA lowest (4.03). Following the PES, EES (73.44), PCB + SBA (67.8), SES (65.83), DCB (59.91), DES (59.5), CBA (48.79), ZES (46.07), PCB (45.01), SCB (38.23), and POBA + CBA (14.94) ranked successively in decreasing order while the remaining interventions had SUCRA values <40, as shown in Figure 3A and B (Supplemental Table S2).

**Figure 4:**
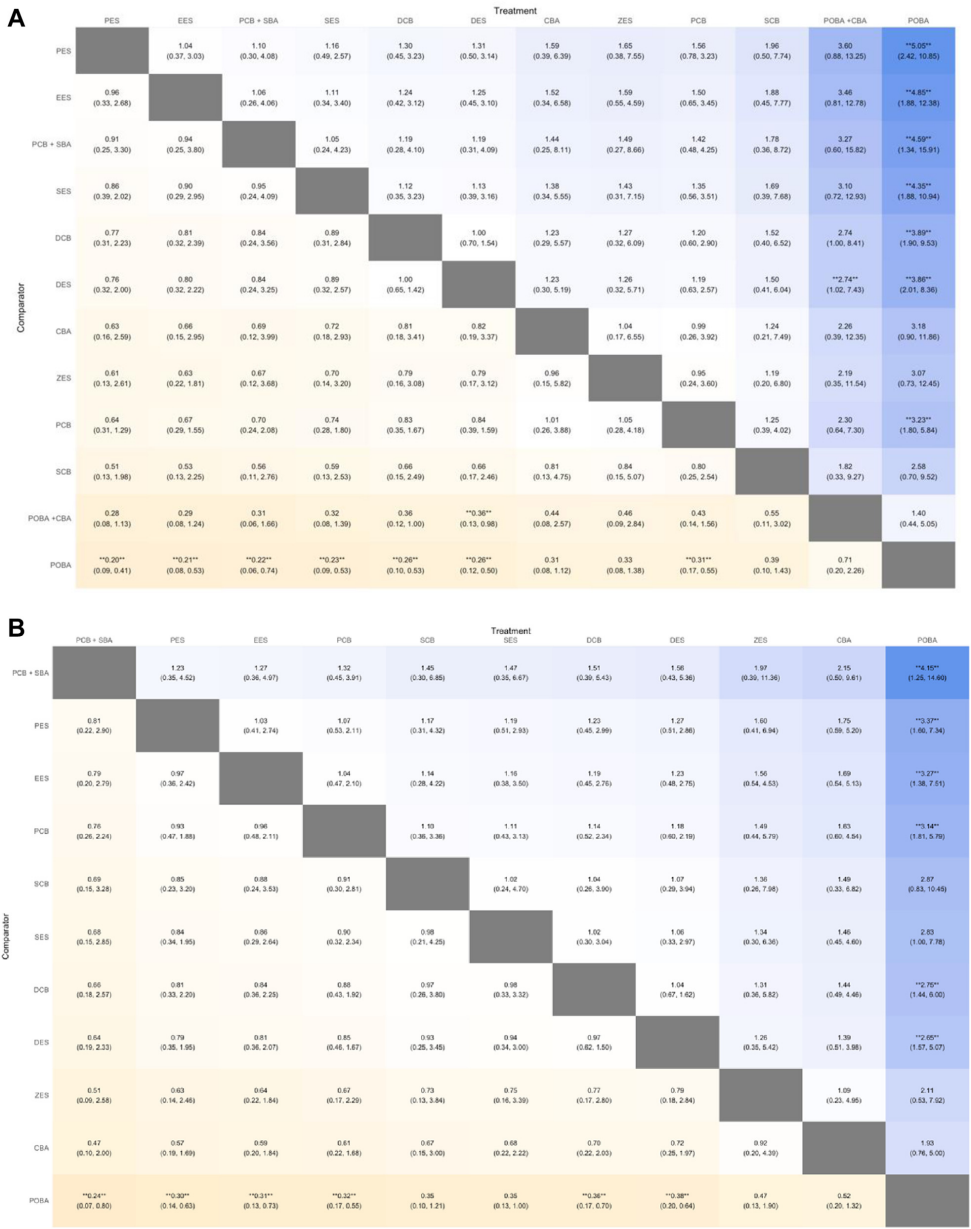
**Ranking chart heat maps.** (A) For TLR and (B) for MACE. Data are ORs (95% CrI) of the treatment on the top, compared with the comparator on the left. OR > 1.0 shows an advantage for the treatment, whereas OR < 1.0 shows an advantage for the comparator. Statistically significant results (*P* < .05) are marked by double asterisks (∗∗). CBA, cutting balloon angioplasty; CrI, credible interval; DCB, drug-coated balloon; DES, drug-eluting stent; EES, everolimus-eluting stent; MACE, major adverse cardiovascular event; OR, odds ratio; PCB, paclitaxel-coated balloon; PES, paclitaxel-eluting stent; POBA, plain balloon angioplasty; SBA, scoring balloon angioplasty; SCB, sirolimus-coated balloon; SES, sirolimus-eluting stent; TLR, target lesion revascularization; ZES, zotarolimus-eluting stent.

The heatmap of league plots of the network estimates confirmed the findings of the NMA forest plot and SUCRA plot. Compared with POBA, treatment of DES-ISR with PES, EES, PCB + SBA, SES, DCB, DES, and PCB might lower the occurrence of TLR events significantly. Compared with POBA + CBA, DES might lower the occurrence of TLR events significantly, as shown in Figure 4A.

In pairwise meta-analysis of pairs of interventions, compared with POBA, there was a significantly lower occurrence of TLR only in the PCB group (Supplemental Figure S9C) but not in the DES group (Supplemental Figure S9B). When compared with the PCB group, EES had a significantly lower occurrence of TLR (Supplemental Figure S9D). There were no differences between DES vs DCB (Supplemental Figure S9A), PES vs DCB (Supplemental Figure S9E), and SCB vs PCB (Supplemental Figure S9F). There was no heterogeneity for EES vs PCB, PES vs PCB, and SCB vs PCB; low to medium level for DES vs DCB and DES vs POBA; and high level for PCB vs POBA (Supplemental Figure S9).

#### MACE

Twenty-five studies reported MACE and assigned 5473 patients with DES-ISR to 11 interventions. Of the 55 possible pairwise comparisons, only 17 had direct comparison data. Of 25 studies, 22 were 2-arm whereas 3 were multiarm reporting MACE in >2 intervention arms. There were 1056 MACE, with no studies having zero events in any arm. The network plot was connected as shown in Figure 1B.

In the NMA forest plot, compared with POBA, DCB (OR, −1.01; 95% CrI, −1.89 to −0.36), DES (OR, −0.98; 95% CrI, −1.62 to −0.45), EES (OR, −1.19; 95% CrI, −2.02 to −0.32), PCB (OR, −1.15; 95% CrI, −1.76 to −0.60), PCB + SBA (OR, −1.42; 95% CrI, −2.68 to −0.23), and PES (OR, −1.22; 95% CrI, −1.99 to −0.47) might significantly lower occurrence of MACE, as shown in Figure 2B.

SUCRA values ranked PCB + SBA (75.88) highest and POBA (3.04) lowest. Following the PCB + SBA, PES (67.66), EES (65.28), PCB (62.68), SCB (54.42), SES (53.48), and DCB (52.13) ranked in decreasing order while the remaining interventions had SUCRA values <50, as shown in Figure 3C and D (Supplemental Table S2).

The heatmap of league plot of the network estimates confirmed the findings of the NMA forest plot and SUCRA plot. Compared with POBA, treatment of DES-ISR with PCB+SBA, PES, EES, PCB, DCB, and DES might lower the occurrence of MACE significantly, as shown in Figure 4B.

In pairwise meta-analysis, compared with POBA, there was significantly lower occurrence of MACE in the PCB group in both the common and random-effect models whereas this occurred in the DES group only in the common effect model (Supplemental Figure S10B and C). Compared with PCB, the EES group had a significantly lower occurrence of MACE in both effect models (Supplemental Figure S10D). There were no differences between DES and DCB, PES and PCB, and SCB and PCB (Supplemental Figure S10A, E, and F, respectively). There was no heterogeneity for EES vs PCB, PES vs PCB, and SCB vs PCB; low-medium level for PCB vs POBA, medium level for DES vs DCB, and high level for DES vs POBA only.

#### MI

Twenty-one studies reported MI events and assigned 6064 patients with DES-ISR to 11 interventions. Of the 55 possible pairwise comparisons, only 14 had direct comparison data. Of 21 studies, 20 were 2-arm whereas 1 was multiarm. There were 325 MI events, with 9 studies with at least one zero event. The network plot was connected as shown in Figure 1E.

In the NMA forest plot, compared with POBA, CBA (OR, −18.56; 95% CrI, −46.17 to −1.66) and SCB (OR, −20.70; 95% CrI, −69.22 to −0.58) might significantly lower the occurrence of MI events (Supplemental Figure S5E).

SUCRA values ranked SCB (93.57) highest, and SES (22.15) ranked lowest. Following SCB, CBA (93.19), PCB (54.96), EES (50.15), POBA + CBA (46.45), DCB (45.79), and DES (45.62) ranked in decreasing order. The remaining interventions had SUCRA values <40 (Supplemental Figure S6E and Supplemental Table S2).

The heatmap of league plots of the network estimates confirmed the findings of the NMA forest plot and SUCRA plot. Treatment of DES-ISR with SCB and CBA might significantly lower the occurrence of MI events compared with PCB, ZES, PES, POBA, and SES. Additionally, treatment of DES-ISR with CBA also might lower the occurrence of MI events significantly compared with EES, POBA + CBA, DCB, and DES (Supplemental Figure S7E).

In pairwise meta-analysis of pairs of interventions, there were no differences between DES vs DCB and EES vs DCB comparisons (Supplemental Figure S13A and B). There was no heterogeneity for DES vs DCB, and medium level for EES vs DCB.

Detailed results on TLF, TVR, all-cause death, cardiac death, and ST are presented and explained in the supplementary files.

### Consistency of direct and indirect evidence

The possibility of global inconsistency was none for TLF (Supplemental Figure S8E) and all-cause death (Supplemental Figure S8H); very low for cardiac death (Supplemental Figure S8I) and ST death (Supplemental Figure S8J); and low for TLR (Supplemental Figure S8A), MACE (Supplemental Figure S8C), TVR (Supplemental Figure S8F), and MI (Supplemental Figure S8G).

### Subanalysis involving the RCTs only

The network plot among the RCTs is shown in Supplemental Figure S17. In the subanalysis of data from 14 RCTs involving 2823 patients with 423 TLR events, compared with POBA, EES (OR, −2.17; 95% CrI, −3.40 to −0.97), PCB (OR, −1.16; 95% CrI, −1.97 to −0.49), PCB + SBA (OR, −1.51; 95% CrI, −3.04 to −0.13), PES (OR, −1.62; 95% CrI, −2.60 to −0.84), and SES (OR, −1.50; 95% CrI, −2.67 to −0.67) might significantly lower the occurrence of TLR events (Supplemental Figure S5A). EES was ranked highest (88.75) and POBA lowest (2.1) in SUCRA values (Supplemental Figure S6A and Supplemental Table S3), and compared with POBA, treatment of DES-ISR with EES, PES, PCB + SBA, SES, and PCB might lower the occurrence of TLR events significantly in the league plot (Supplemental Figure S7A).

In the subanalysis of data from 13 RCTs involving 2626 patients with 506 MACE, compared with POBA, CBA (OR, −1.28; 95% CrI, −2.55 to −0.10), EES (OR, −1.76; 95% CrI, −2.76 to −0.78), PCB (OR, −1.30; 95% CrI, −2.06 to −0.73), PCB + SBA (OR, −1.69; 95% CrI, −2.92 to −0.44), PES (OR, −1.38; 95% CrI, −2.25 to −0.67), and SES (OR, −1.31; 95% CrI, −2.44 to −0.33) might significantly lower the occurrence of the MACE (Supplemental Figure S5B). EES was ranked highest (81.51) and POBA lowest (1.23) (Supplemental Figure S6B and Supplemental Table S3).

### Consistency of direct and indirect evidence

The possibility of global inconsistency was none for MACE (Supplemental Figure S8D) and very low for TLR (Supplemental Figure S8B).

## Discussion

This NMA represents a comprehensive synthesis of data for the different PCI strategies for coronary DES-ISR from the currently available studies. On Bayesian network analysis, our findings can be summarized as follows: PES (76.42%) is the most effective strategy for TLR; PCB and SBA (75.88%) for MACE; SCB for TLF (64.16%), MI (93.57%), and ST (98.53%); PCB for all-cause death (76.39%) and cardiac death (83.74%); and POBA (96.32%) ranked the most effective strategy for TVR. Conversely, for BA-based strategies: POBA, SBA, and CBA were deemed to be the least or less effective alternatives to the treatment of DES-ISR with DCB or DES in terms of clinical safety and efficacy outcomes. In subanalysis involving the randomized studies only, EES is the best strategy for TLR (88.75%) and MACE (77.53). Based on pairwise analysis of direct evidence, PCB is better than POBA for TLR and MACE; DES is better compared with DCB only for cardiac death, while they are not different from each other for TLR, MACE, TLF, TVR, all-cause death, and ST.

In the AGENT IDE trial,^12^ among coronary BMS- or DES-ISR patients undergoing coronary angioplasty, a PCB was superior to POBA for TLF, TLR, TVR, and ST. In a review, Dangas et al^58^ recommended different types of DES and BA for DES-ISR according to the potential underlying mechanisms because of the dilemma regarding DCB. Zhu et al^59^ found that DES was superior to DCB for TLR and TVR in coronary DES-ISR patients. In an NMA by Lee et al^14^ comparing drug-eluting balloon (DEB), DES, and POBA for BMS- or DES-ISR, DEB and DES were similar to each other, but both were superior to POBA in preventing MACE and TLR. Siontis et al^13^ recommended PCI with EES for better angiographic and clinical results and DCB for their ability to provide favorable results without adding a new stent layer for BMS- or DES-ISR. In the DAEDALUS study, Lansky et al^60^ found that, among patients with BMS- or DES-ISR, DES had superiority over PCB for TLR. However, in a systematic review and meta-analysis by Xi et al,^61^ there was no difference in the safety and efficacy of DCB and DES in the treatment of BMS or DES coronary ISR. In a recent NMA by Guo et al,^8^ DES, especially EES (in terms of TLR, all-cause death, MACE, percentage diameter stenosis, and binary restenosis) and DCB, especially SCB (in terms of late luminal loss) were preferred strategies over BA, vascular brachytherapy, rotational atherectomy, and BMS for BMS or DES coronary ISR. Despite a growing number of randomized, nonrandomized, and review studies reporting variable findings, the majority had a mixture of BMS- and DES-ISR patients despite significant differences in their underlying mechanism, which can affect the outcomes.^8^^,^^12^, ^13^, ^14^^,^^60^^,^^61^

ISR is still a significant complication following PCI for MI despite significant advancements in coronary angiography techniques as well as intervention devices. The optimal treatment strategies for the treatment of coronary ISR are still debated. The existing recommendations for coronary ISR are extended to both as the same entity.^9^^,^^10^^,^^58^^,^^59^ In this NMA, we found that DCB and DES are still the best choice for the treatment of coronary DES-ISR, consistent with previous study findings.^8^^,^^62^^,^^63^ DCB and its different variants such as SCB and PCB are the best strategies for treatment of DES-ISR with respect to clinical safety and efficacy because they had >70% probability of being the best treatment among the different PCI strategies for coronary DES-ISR in the overall analysis as well as in the subanalysis. Similar to our findings, Siontis et al^13^ also reported EES as the most effective strategy for coronary DES-ISR patients for TLR (SUCRA value, 93.1). Similarly, Giacoppo et al^63^ also had a contrasting finding of DES as the best treatment strategy for coronary BMS- or DES-ISR for TLR (SUCRA value, 61.4%). In the DAEDALUS study, DES was superior to DCB in reducing TLR and ischemia-driven TLR among coronary ISR patients.^60^

In contrast to our finding, Piccolo et al^62^ reported that DCB (paclitaxel-eluting balloon) was the most effective strategy for the treatment of DES-ISR for TLR (SUCRA value, 55%), along with angiographic outcomes in their systematic review and meta-analysis of 7 RCTs. Almalla et al^40^ also found the superiority of DCB to EES for TLR, contrasting to our finding, and for MACE, a similar finding as ours, in an observational study involving 86 DES-ISR patients. Wang et al^38^ also found similar results with superiority of DCB over repeat DES for DES-ISR for TLR and MACE. An NMA of 11 RCTs by Lee et al^14^ of patients having BMS/DES-ISR found that DCB had the highest probability of being ranked the first treatment option for TLR, MI, all-cause mortality, and MACE. In a network analysis of 5923 patients with BMS- or DES-ISR, Siontis et al^13^ found DCB as the most effective strategy in terms of all-cause mortality for coronary DES-ISR (SUCRA value, 84.9). Similarly, Condello et al^64^ found superiority of DCB over DES with regard to MACE, MI, and ST with no differences between them for TLR and cardiac death. In contrast to our finding, Siontis et al^13^ also reported contrasting findings in terms of MI with EES as the most effective strategy for coronary DES-ISR patients (SUCRA value, 80.7). In an NMA of 10 different PCIs among 8479 BMS/DES-ISR patients, Guo et al^8^ found EES as the most effective intervention device to reduce TLR, all-cause death, and MACE. These contrasting findings could be differences in the cohort of patients included in the previous studies, which involved both BMS-ISR and DES-ISR, whereas we involved only DES-ISR patients. The involvement of BMS-ISR patients along with DES-ISR patients might affect the outcomes for different interventions for ISR due to differences in the underlying path-mechanisms of BMS-ISR and DES-ISR.

Although the mechanisms underlying ISR affecting BMS and DES overlap, they have substantial differences in time course, phenotypic appearance, and underlying mechanism. DES-ISR is more frequently associated with focal or edge-related NIH, with late neoatherosclerosis being the more profound pathomechanism^6^^,^^65^ along with elastic recoil and relocation/subluxation of axially transmitted plaque (tissue intrusion) (especially early) and reorganization of thrombus, neointima formation, and remodeling.^7^^,^^66^ In addition to this, hypersensitivity to the polymer and the drug, local inflammation, and delayed healing are also the other main contributors to neointima formation with DES-ISR.^11^^,^^66^ Neoatherosclerosis affecting ISR is a chronic inflammatory response to the stent itself.^67^ The sustained drug release in DES is responsible for delayed healing, thus increasing the risk of late thrombosis.^68^ DES generally elutes the drug primarily to the stented-dilated area, whereas DCBs have the potential to deliver an identical amount of paclitaxel proximally and distally to the stented segments.^69^ Theoretically, DCB may have some advantages over stent-based local drug delivery: broader area of surface contact and more homogenous drug–tissue transfer, potential amelioration of delayed arterial healing owing to the absence of long-term inflammatory nidus, preservation or early restoration of normal vessel anatomy, and function at the cost of the lower acute gain and potentially more unstable acute results.^70^ Giustino et al^6^ highlighted the ISR with a less aggressive pattern (eg, focal), which reaches good lumen expansion after balloon dilatation, as a favorable factor to prefer DCB, which is often seen with DES-ISR. Additionally, DCB offers the advantage of avoiding the implantation of an additional metallic layer and thus further enhances exuberant NIH and provision of the substratum for developing neoatherosclerosis.^66^

The management of DES-ISR can be guided by imaging modalities such as intravascular ultrasound (IVUS) or optical coherence tomography (OCT), and they offer superiority over invasive angiography for better characterization of ISR and morphology, and thus the selection of appropriate treatment strategy.^6^^,^^71^ IVUS provides a better delineation of the stent-related vascular remodeling, stent underexpansion, NIH distribution, and accurate vessel sizing and thus guides the optimal stent expansion.^6^ OCT offers better characterization of the neoatherosclerosis, delineation of the lumen neointimal interface, and stent struts distribution. OCT classification of neoatherosclerosis based on OCT by Ali et al^72^ could have a pivotal role in directing the selection of the appropriate treatment modality. OCT has some limitations such as the need to use contrast media to ensure a blood-free field for image acquisition, which can be difficult for some lesions such as ostial or tight types. Despite these promising roles of OCT and IVUS in DES-ISR management, there is a lack of sufficient evidence to make recommendations.^6^^,^^71^

### Strengths and limitations of study

Our NMA had several strengths. First, we incorporated randomized and nonrandomized studies as well as performed subanalysis involving randomized studies. Second, we included different modalities of DCB, DES, and BA that are commonly used in practice such as SCB, PCB in DCB; SES, EES, ZES, PES in DES; POBA, SBA, and CBA. Third, we conducted a detailed search of the databases, the references of included studies for data extraction, clinical registry trials, followed national conferences, and critically appraised the eligibility of study inclusion, data extraction, and quality assessment of included studies. Finally, we ranked the different PCI strategies based on SUCRA curves as well as estimated summary ORs using NMA forest plots and league plots with 95% CrIs.

We acknowledge that there are several limitations despite the strengths of our study. First, we did not incorporate studies comparing different commercial variants of the same device as Shenqi PCB vs SeQuent Please PCB or homo-DES vs hetero-DES, which can be a common scenario in clinical practice. Second, we did not incorporate the angiographic outcomes as they were not reported in most of the nonrandomized studies. Third, we only utilized the published and available data to analyze the average treatment clinical safety and efficacy because relevant data at the individual patient level were unavailable. Fourth, there was heterogeneity across the included studies (both within and among randomized as well as nonrandomized studies) regarding the number of participants; underlying comorbidities; the period of investigation; coronary angiography procedures such as IVUS, OCT, and invasive angiography; duration of follow-up; and duration of dual antiplatelet therapy. Fifth, the different types of DES and DCB were grouped together in the nonrandomized studies as well as in DES arms of many RCTs. Finally, we did not analyze the cost-effectiveness of different strategies and PCI procedure-associated side effects/complications.

## Conclusion

Based on the findings of this NMA, either DES or DCB PCIs such as PCB and SCB should be considered for treatment of coronary DES-ISR to achieve most of the clinical efficacy and safety benefits in terms of MACE and its components. However, DCB and DES remain the preferred treatment strategies for coronary DES-ISR, compared with BA-based PCI strategies. More robust evidence is lacking in terms of different approaches for DES-ISR treatment. With US Food and Drug Administration approval of DCB therapy for coronary ISR in the United States, the findings of this NMA along with the breakthroughs in this field will encourage more studies focused on different DCB and DES strategies and their comparison to other approaches for DES-ISR in the near future to provide more definite answers to this debated topic.
