# Supplementary material for: Comparison of Different PCI Strategies for Coronary DES In-stent Restenosis: A Bayesian Network Meta-analysis
Source: J Soc Cardiovasc Angiogr Interv. 2025 Jan 31;4(3Part A):102428. doi: 10.1016/j.jscai.2024.102428 (PMC11993875; doi:10.1016/j.jscai.2024.102428)
Supplement: Supplementary File 1 [file mmc1.docx]

**Supplementary file**

**Table 1.** Quantitative coronary angiography characteristics among included patients.

| **Study** | **Intervention** | **Quantitative coronary angiography** | | | | | | | |
| --- | --- | --- | --- | --- | --- | --- | --- | --- | --- |
|  |  | **Pre-procedure** | | | | **Post-procedure** | | | |
|  |  | **RFD, mm** | **MLD, mm** | **Stenosis, % of lumen diameter** | **Lesion length, mm** | **RFD, mm** | **MLD, mm** | **Stenosis, % of lumen diameter** | **Acute gain, mm** |
| **Randomized controlled trials** | | | | | | | | | |
| RIBS IV trial ^30^ | DCB, n= 154 | 2.59 (0.5) | 0.79 (0.4) | 69 (17) | 10.4 (5.6) | 2.64 (0.5) | 2.20 (0.4) | 16 (10) | 1.40 (0.5) |
|  | EES, n= 155 | 2.67 (0.5) | 0.75 (0.4) | 72 (15) | 10.7 (5.4) | 2.70 (0.5) | 2.49 (0.5) | 8 (11) | 1.75 (0.6) |
| Song et al. ^31^ | Focal lesion, SES, n= 48 | 3.13 (4.4) | 0.68 (0.28) | 77.04 (9.71) | 9.14 (4.43) | 2.56 (0.46) | 8.05 (5.96) |  | 1.89 (0.50) |
|  | Focal lesion,  CBA, n= 48 | 3.03 (0.24) | 0.78 (0.36) | 72 (13.76) | 6.92 (3.07) | 2.45 (0.57) | 7.35 (2.29) |  | 1.70 (0.52) |
|  | Diffuse lesion,  SES, n= 32 | 3.17 (0.50) | 0.92 (.056) | 69.88 (21.73) | 22.18 (9.02) | 2.38 (0.35) | 17.93 (9.16) |  | 1.45 (0.78) |
|  | Diffuse lesion,  EES, n= 34 | 3.41 (0.49) | 0.80 (0.70) | 74.88 (20.51) | 24.75 (15.90) | 2.46 (0.70) | 17.82 (11.22) |  | 1.60 (1.03) |
| Montorsi et al. ^29^ | PTCA, n= 25 | 3.19 (0.33) | 1.07 (0.35) | 66 (10) |  | 3.22 (0.29) | 2.01 (0.42) | 37 (13) |  |
|  | CBA, n= 25 | 3.28 (0.53) | 0.93 (0.38) | 68 (11) |  | 3.21 (0.47) | 2.25 (0.30) | 26 (11) |  |
| PEPCAD-DES trial ^32^ | PCB, n= 72 | 2.29 (0.51) | 0.66 (0.40) | 72.1 (14.5) | 11.2 (6.5) | 2.47 (0.45) | 2.15 (0.42) | 12.6 (6.2) |  |
|  | POBA, n= 38 | 2.30 (0.52) | 0.62 (0.44) | 74.0 (16.2) | 12.2 (8.2) | 2.47 (0.58) | 2.14 (0.53) | 13.7 (6.0) |  |
| PEPCAD China ISR trial ^33^ | PCB, n= 109 | 2.66 (0.38) | 0.85 (0.38) | 68.26 (12.47) | 12.52 (6.55) | 2.59 (0.40) | 2.25 (0.38) | 12.86 (8.34) | 1.40 (0.44) |
|  | PES, n= 106 | 2.72 (0.44) | 0.86 (0.41) | 68.43 (13.25) | 13.08 (7.13) | 2.67 (0.44) | 2.32 (0.47) | 13.00 (8.75) | 1.47 (0.50) |
| RESTORE trial ^39^ | PCB, n= 86 | 2.85 (0.50) | 0.63 (0.40) | 77 (17) | 18.1 (9.7) | 2.68 (0.50) | 1.97 (0.43) | 26 (10) | 1.34 (0.52) |
|  | EES, n= 86 | 3.06 (0.45) | 0.63 (0.42) | 79 (13) | 17.4 (11.4) | 2.82 (0.49) | 2.24 (0.48) | 20 (11) | 1.61 (0.54) |
| ISAR-DESIRE 4 trial ^34^ | DCB + SBA, n= 125 | 2.96 (0.50) | 1.01 (0.46) | 65.7 (14.1) |  |  | 2.37 (0.47) | 21.6 (9.5) |  |
|  | DCB, n= 127 | 2.89 (0.48) | 0.94 (0.36) | 67.2 (12.2) |  |  | 2.28 (0.40) | 22.3 (9.9) |  |
| ISAR-DESIRE 3 trial ^42^ | PCB, n= 137 | 2·75 (0·50) | 0·97 (0·48) | 64·4 (16·8) |  |  | 2·29 (0·44) | 18·5 (8·3) |  |
|  | PES, n= 131 | 2·80 (0·49) | 0·93 (0·50) | 66·7 (16·5) |  |  | 2·53 (0·48) | 12·8 (7·8) |  |
|  | POBA, n= 134 | 2·72 (0·45) | 0·88 (0·49) | 67·7 (15·7) |  |  | 2·10 (0·49) | 23·3 (12·6) |  |
| ISAR-DESIRE 2 trial ^35^ | SES, n= 225 | 2.78 (0.47) | 1.02 (0.55) | 63.4 (18.3) | 12.7 (8.3) |  | 2.20 (0.54) | 24.0 (11.4) |  |
|  | PES, n=225 | 2.75 (0.48) | 0.95 (0.48) | 65.6 (16.0) | 12.5 (7.7) |  | 2.18 (0.53) | 24.1 (11.8) |  |
| RESTENT-ISR trial ^41^ | EES, n= 158 | 3.07 (0.50) | 0.78 (0.45) | 74.1 (14.7) | 17.2 (8.4) | 3.20 (0.50) | 2.82 (0.50) | 11.9 (11.4) | 2.05 (0.62) |
|  | ZES, n= 146 | 3.15 (0.53) | 0.89 (0.62) | 72.2 (17.1) | 17.8 (9.4) | 3.25 (0.53) | 2.86 (0.47) | 11.8 (11.2) | 2.01 (0.67) |
| Habara et al. ^36^ | PCB, n= 25 | 2.69 (0.36) | 0.99 (0.32) | 64.1 (9.9) | 12.7 (5.3) | 2.74 (0.34) | 1.99 (0.26) | 25.7 (7.2) | 1.03 (0.31) |
|  | POBA, n= 25 | 2.90 (0.47) | 0.92 (0.51) | 68.4 (16.9) | 13.2 (5.5) | 2.90 (0.46) | 2.00 (0.51) | 31.0 (8.9) | 1.10 (0.54) |
| CRISTAL trial ^38^ | SES, n= 136 | 2.6 (0.40) | 1.09 (0.50) | 58.75 (16.30) | 14.6 (9.0) |  | 2.51 (0.46) | 9.47 (7.72) | 1.39 (0.52) |
|  | POBA, n= 61 | 2.5 (0.41) | 1.18 (0.58) | 53.70 (20.3) | 13.4 (8.5) |  | 2.12 (0.39) | 18 (9.23) | 0.90 (0.57) |
| Ali et al. ^40^ | DCB, n= 25 | 2.42 (0.54) | 0.80 (0.52) | 69.3 (19.6) | 13.29 (7.18) | 2.40 (0.52) | 2.30 (0.42) | 5.4 (4.3) | 1.63 (0.67) |
|  | SCB, n= 25 | 2.53 (0.53) | 0.81 (0.35) | 67.4 (13.5) | 14.24 (7.83) | 2.46 (0.49) | 2.08 (0.54) | 8.3 (4.2) | 1.44 (0.44) |
| AGENT IDE trial ^12^ | PCB, n= 406 | 2.7 (0.5) | 1.0 (0.4) | 65.0 (12.1) | 12.8 (6.3) |  |  |  |  |
|  | POBA, n= 194 | 2.7 (0.5) | 0.9 (0.4) | 66.4 (12.8) | 11.8 (6.6) |  |  |  |  |
| Scheller B et al. ^37^ | SCB, n= 50 | 2.56 (0.51) | 0.81 (0.44) | 62.8 (24.4) | 13.72 (7.16) | 2.58 (0.53) | 2.39 (0.42) | 5.4 (4.5) | 1.63 (0.63) |
|  | PCB, n= 51 | 2.65 (0.52) | 0.82 (0.39) | 70.4 (16.3) | 13.81 (8.20) | 2.64 (0.50) | 2.31 (0.40) | 7.0 (4.9) | 1.60 (0.49) |
| **Non-randomized studies** | | | | | | | | | |
| Kang et al. ^47^ | DCB, n= 182 | 2.7 (0.5) | 0.8 ( 0.4) | 71.7 ( 15.2) | 19.5 ( 8.9) | 2.8 ( 0.5) | 2.2 ( 0.4) | 20.6 ( 11.9) | 1.5 ( 0.6) |
|  | DES, n= 56 | 3.0 ( 0.4) | 0.8 ( 0.6) | 74.6 ( 19.2) | 21.3 ( 11.8) | 3.1 ( 0.4) | 2.7 ( 0.4) | 13.6 ( 10.5) | 1.9 ( 0.7) |
| Habara et al. 2016 ^49^ | PCB, n=260 | 2.89 (0.43) | 0.81 (0.52) | 71.8 (17.3) | 17.1 (8.6) |  | 2.08 (0.45) | 28.8 (9.6) | 1.27 (0.62) |
|  | DES, n= 425 | 2.99 (0.48) | 0.86 (0.54) | 70.8 (17.3) | 15.7 (11.1) |  | 2.64 (0.49) | 15.1 (8.8) | 1.78 (0.66) |
| Wang et al. ^57^ | DES, n= 79 | 2.79 ( 0.53) | 0.57 ( 0.08) | 69.7 ( 7.9) | 19.3 ( 8.5) |  | 2.67 ( 0.23) | 11.3 ( 3.2) |  |
|  | DCB, n= 93 | 2.65 ( 0.67) | 0.55 ( 0.07) | 71.3 ( 8.3) | 18.7 ( 7.9) |  | 2.51 ( 0.34) | 22.4 ( 4.3) |  |
| Basavarajaiah et al. ^50^ | DCB, n= 81 | 2.68. (0.60) | 0.51 (0.41) |  | 12.3 (11.0) | 2.83 (0.46) | 2.50 (0.50) |  |  |
|  | DES, n= 166 | 2.58 (0.27) | 0.57 (0.30) |  | 9.0 (5.2) | 2.71 (0.32) | 2.42 (0.36) |  |  |
| Kawamoto et al. ^53^ | DES, n= 68 | 2.92 (0.54) | 0.66 (0.43) | 81.2 (14.4) | 16.1 (9.6) |  | 2.65 (0.48) | 13.8 (7.6) | 2.09 (0.53) |
|  | DCB, n= 65 | 2.84 (0.46) | 0.74 (0.49) | 74.8 (15.8) | 18.7 (14.6) |  | 2.34 (0.54) | 18.2 (8.6) | 1.60 (0.62) |
| Kook et al. ^44^ | DES, n= 51 | 2.94 (0.36) | 0.74 (0.61) | 74.98 (20.09) | 19.12 (8.14) | 3.12 (0.39) | 2.85 (0.55) | 8.68 (12.70) | 2.12 (0.80) |
|  | DCB, n= 24 | 3.03 (0.77) | 0.62 (0.47) | 79.75 (13.39) | 18.46 (4.56) | 3.08 (0.74) | 2.71 (0.29) | 10.00 (9.91) | 2.09 (0.50) |

**Table 2.** Sucratable for different outcomes for different interventions in RE model (All studies)

| Interventions | Outcomes | | | | | | | |
| --- | --- | --- | --- | --- | --- | --- | --- | --- |
|  | TLR | MACE | TLF | TVR | MI | All-cause death | Cardiac death | Stent thrombosis |
| DCB | 59.51 | 52.13 | 41.16 | 46.67 | 45.79 | 53.62 | 16.64 | 56.53 |
| PCB | 45.01 | 62.68 | 55.98 | 37.36 | 56.96 | 76.39 | 83.74 | 77.04 |
| PCB+SBA | 67.8 | 75.88 |  |  |  | 73.13 |  |  |
| SCB | 38.23 | 54.42 | 64.16 |  | 93.57 | 71.8 |  | 98.53 |
| DES | 59.5 | 48.34 | 56 | 56.53 | 45.62 | 49.73 | 7.82 | 49.37 |
| SES | 65.83 | 53.48 |  | 61.37 | 22.15 | 21.29 | 61.73 | 1.92 |
| EES | 73.44 | 65.28 |  | 50.34 | 50.15 | 71.17 | 67.69 | 21.57 |
| PES | 76.42 | 67.66 | 56.44 | 53.92 | 29.99 | 15.89 |  | 73.78 |
| ZES | 46.07 | 36.5 |  |  | 37.81 | 47.5 |  | 28.85 |
| POBA | 4.03 | 3.04 | 26.25 | 96.32 | 29.11 | 23.26 | 48.16 | 49.85 |
| CBA | 48.79 | 30.59 |  | 27.79 | 93.19 |  |  | 46.01 |
| POBA+CBA | 14.94 |  |  | 19.7 | 46.45 | 46.2 |  | 46.55 |

NB: *

| **Color** | **Inference** |
| --- | --- |
|  | Highest SUCRA value |
|  | Lowest SUCRA value |

**Table 3.** Sucratable for different outcomes for different interventions in RE model and FE model (Sub-analysis involving RCTs only)

| Interventions | Outcomes | |
| --- | --- | --- |
|  | TLR | MACE |
| DCB |  |  |
| PCB | 35.92 | 44.34 |
| PCB+SBA | 52.23 | 62.81 |
| SCB | 31.1 | 42.18 |
| DES |  |  |
| SES | 57.71 | 53.32 |
| EES | 88.75 | 77.53 |
| PES | 66.17 | 52.52 |
| ZES | 64.59 | 49.28 |
| POBA | 2.1 | 2.73 |
| CBA | 45.43 | 72.04 |
| POBA+CBA |  |  |

NB: *

| **Color** | **Inference** |
| --- | --- |
|  | Highest SUCRA value |
|  | Lowest SUCRA value |


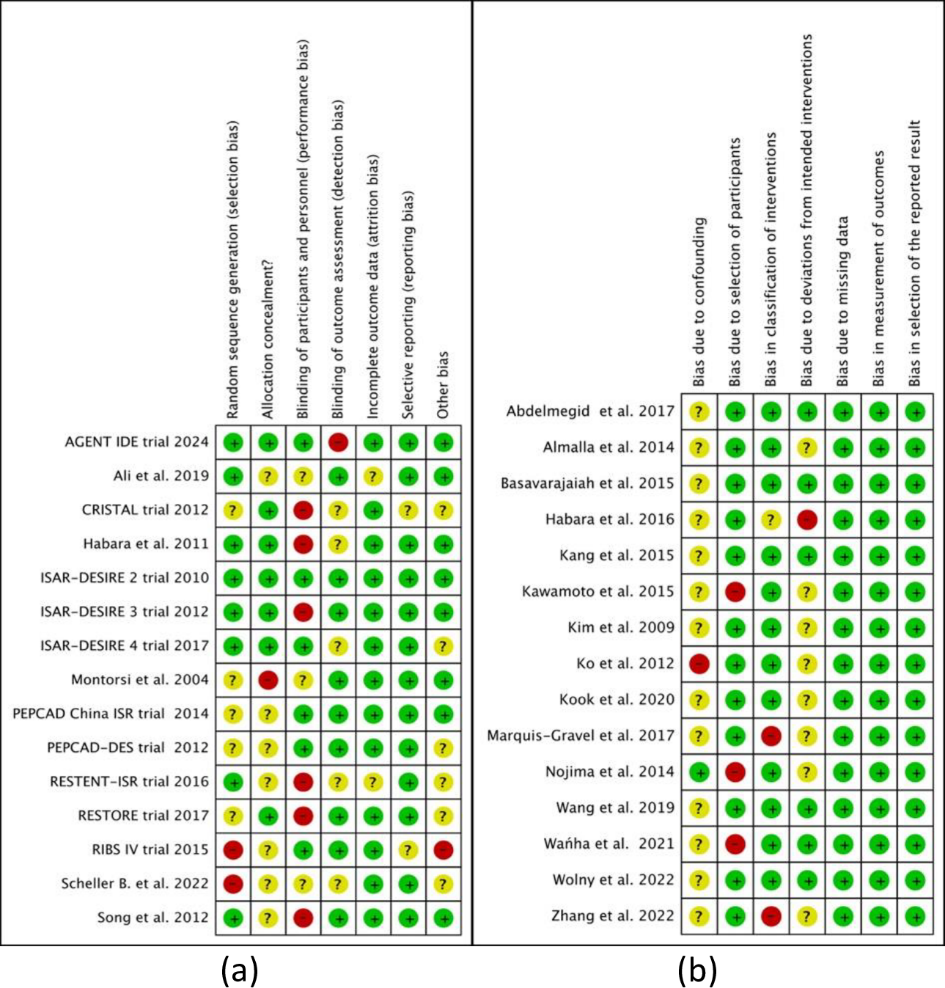


**Figure 1.** Risk of bias: (a) for RCTs: and (b) for non-randomized studies


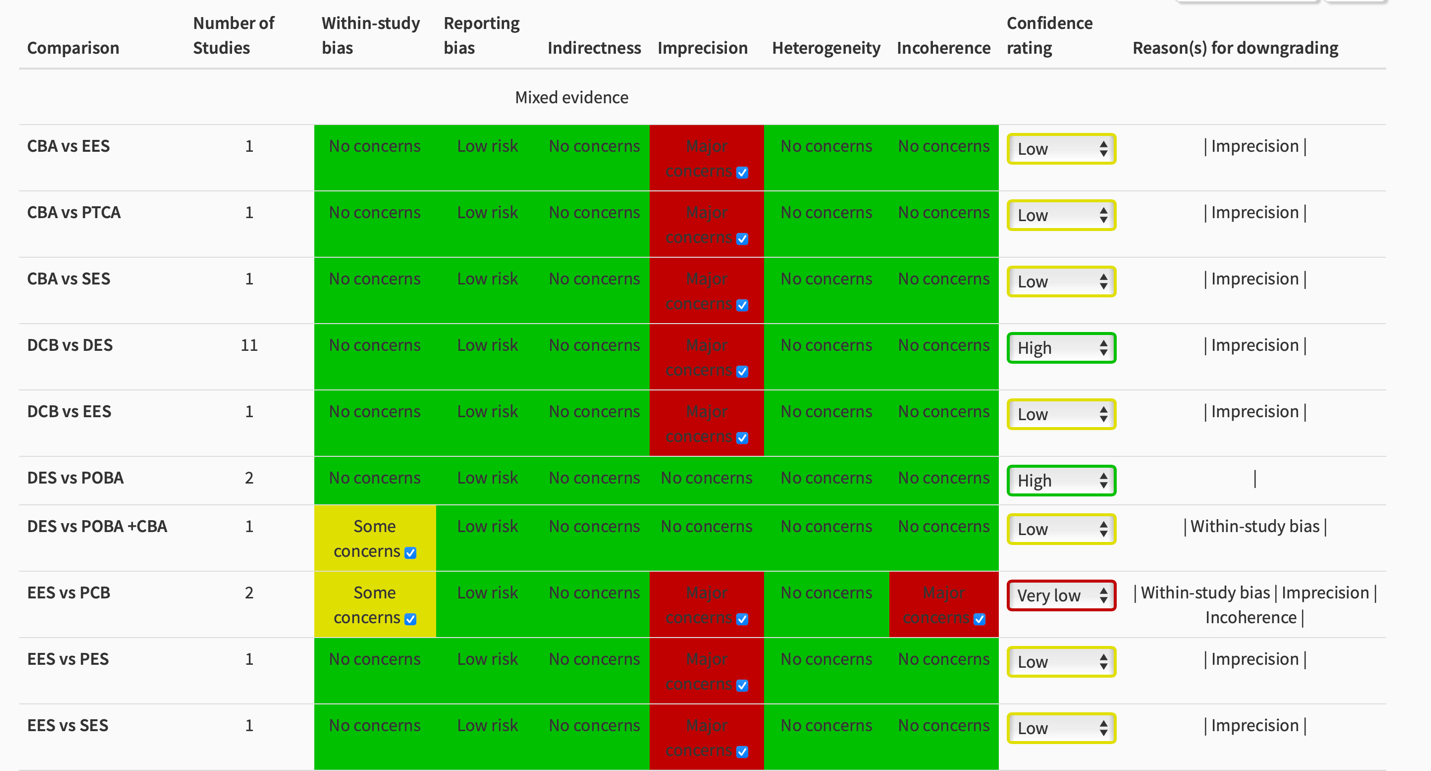

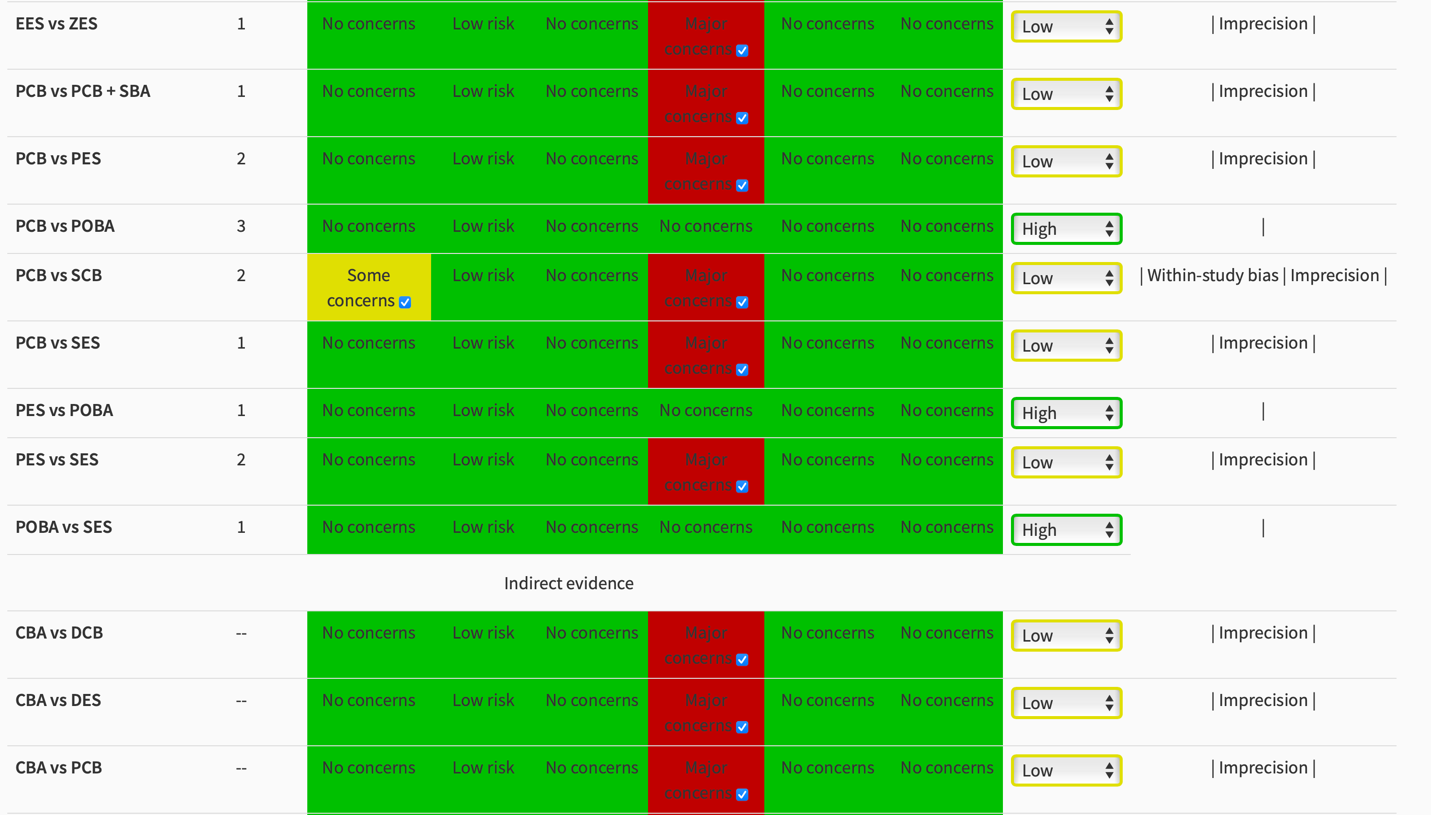

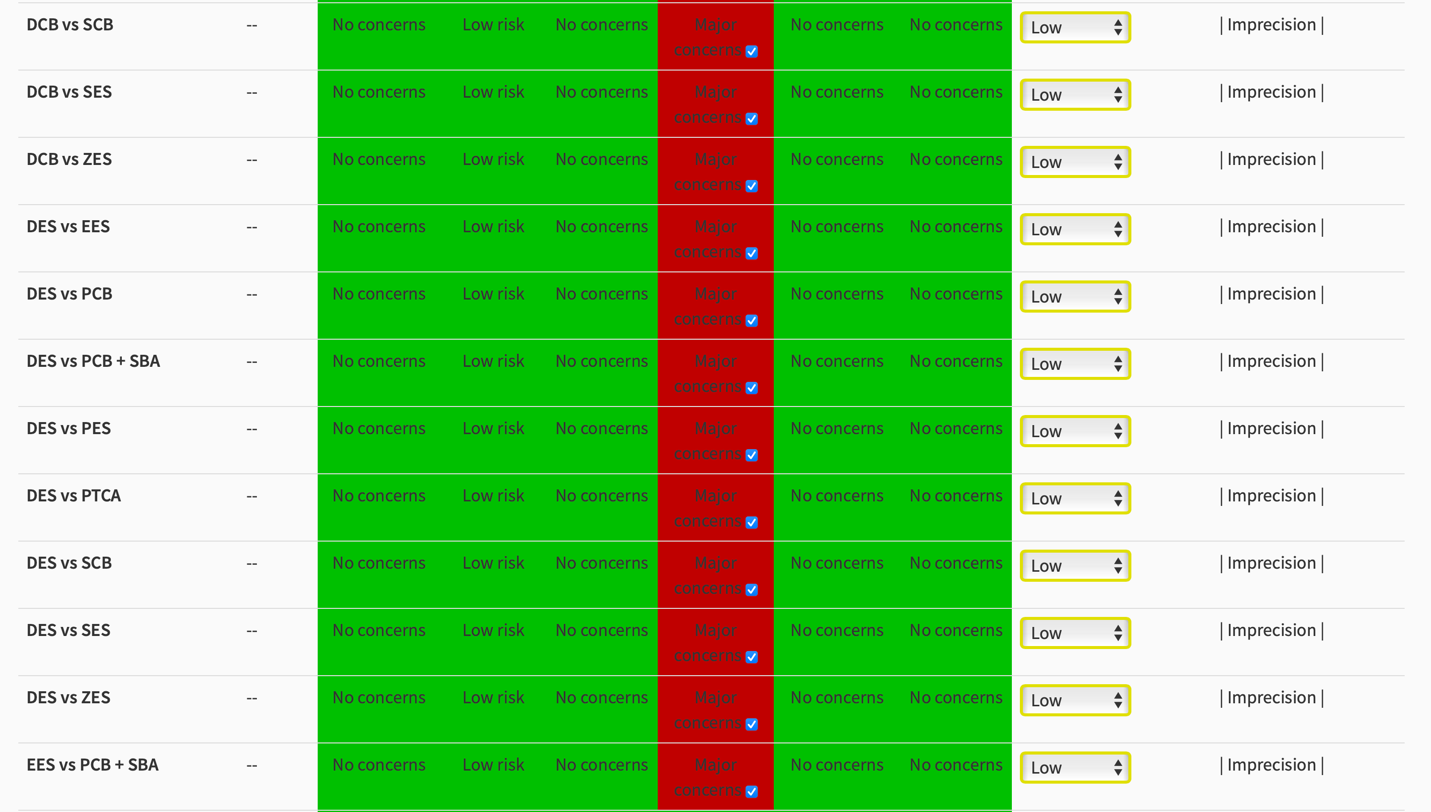

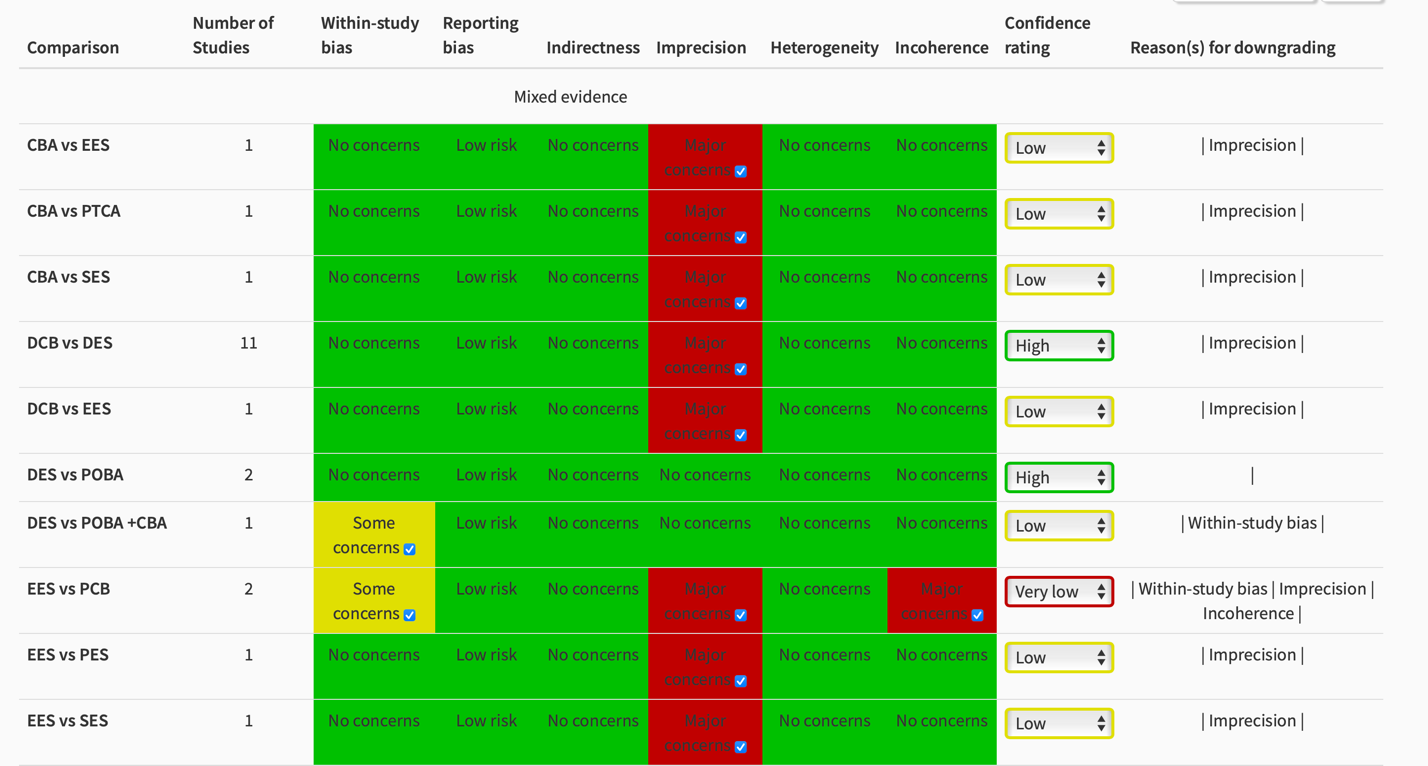

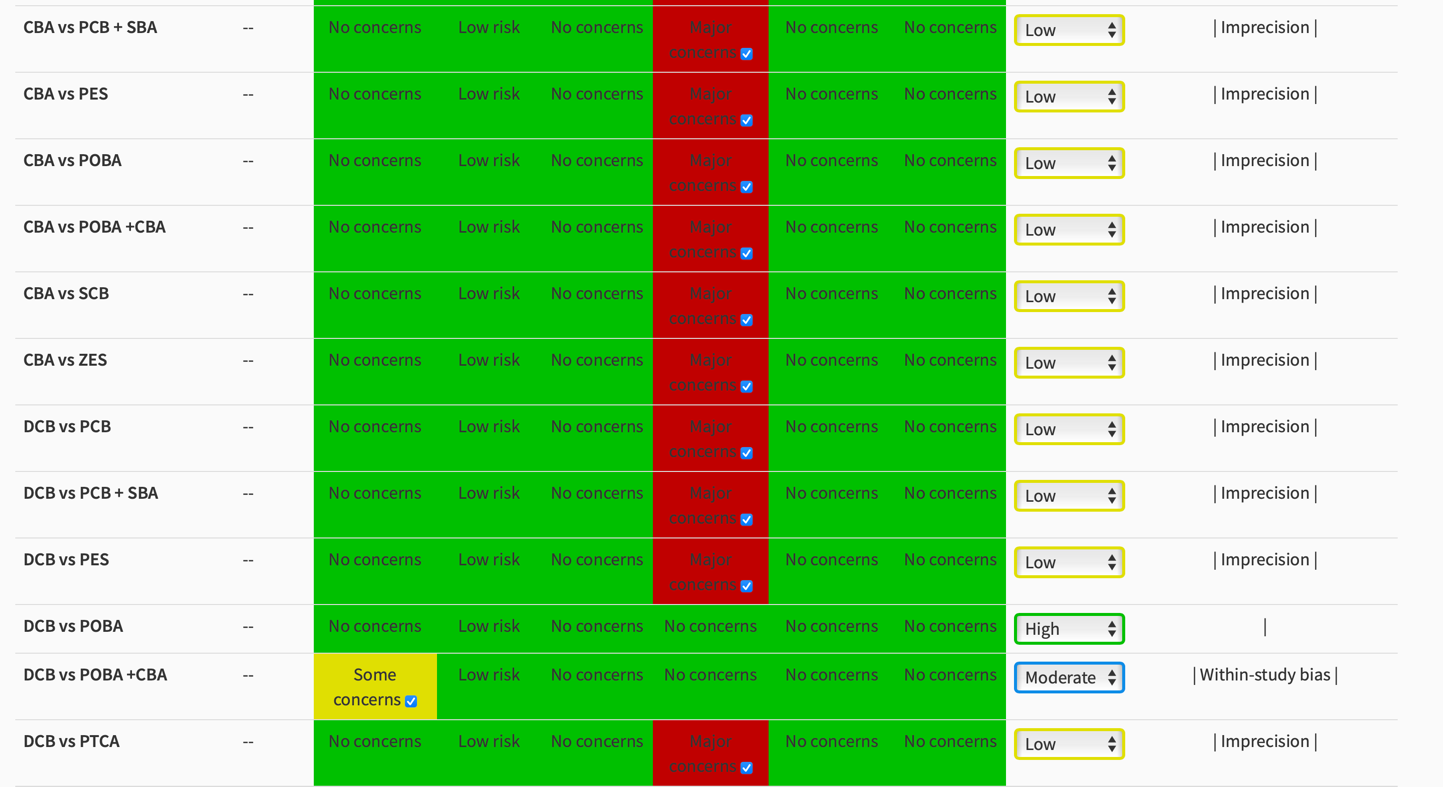

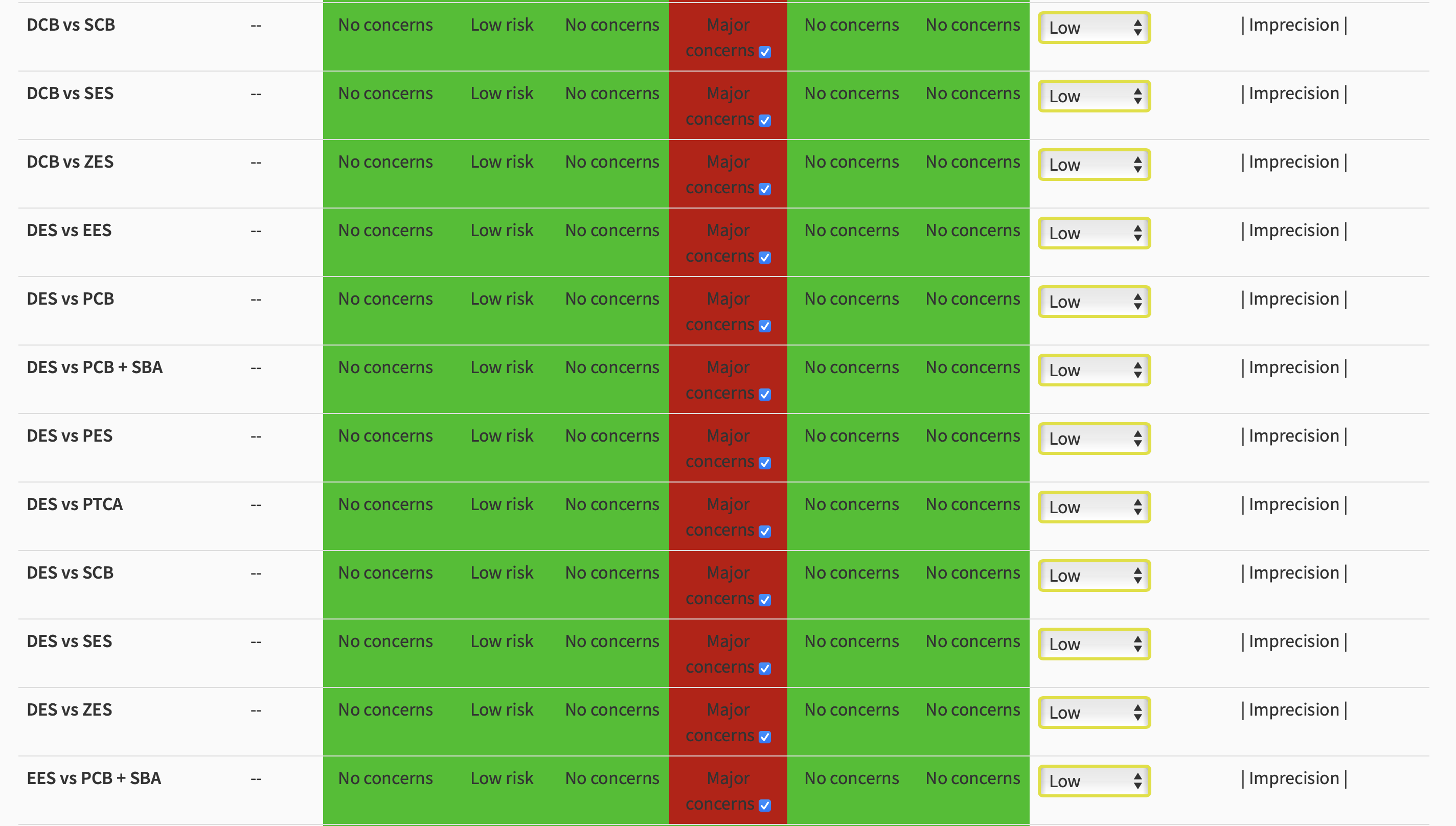

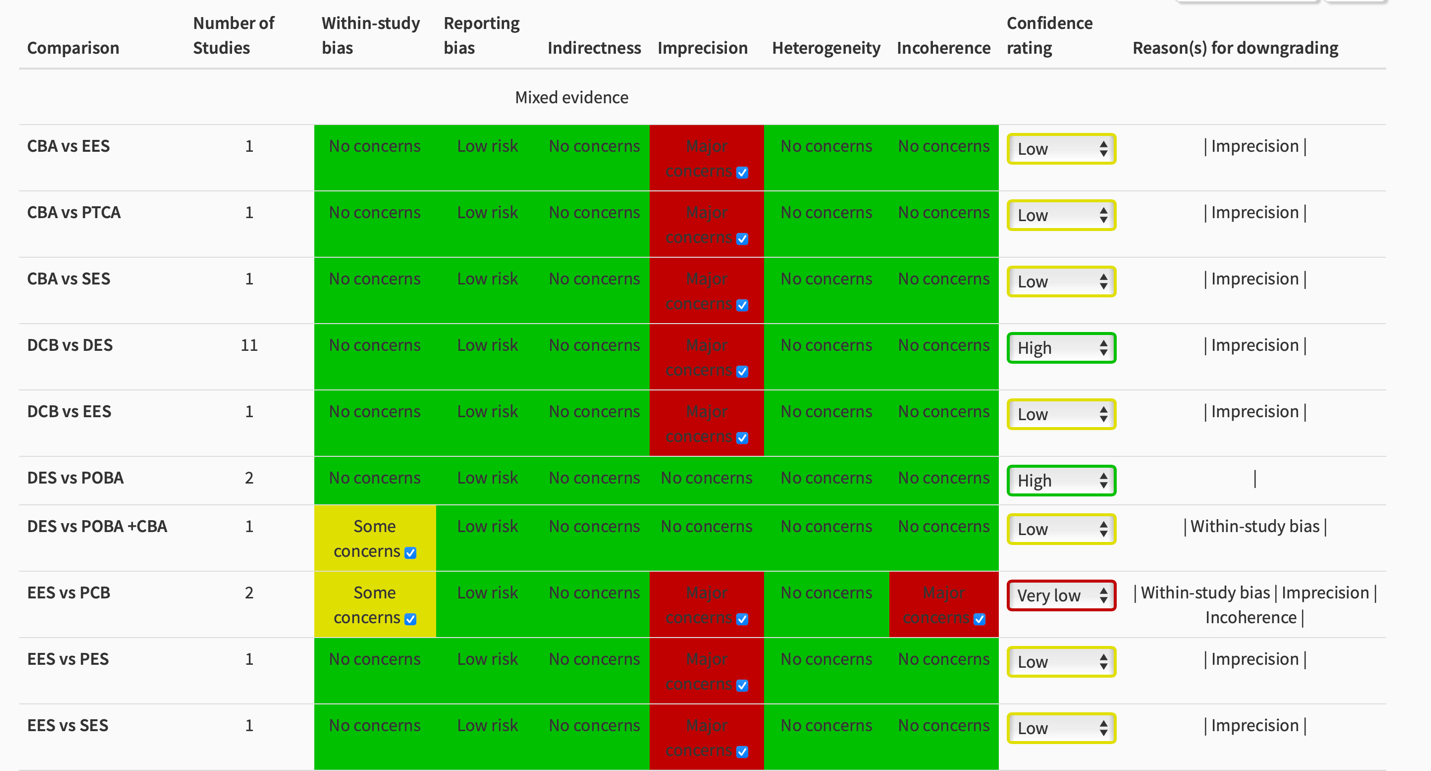

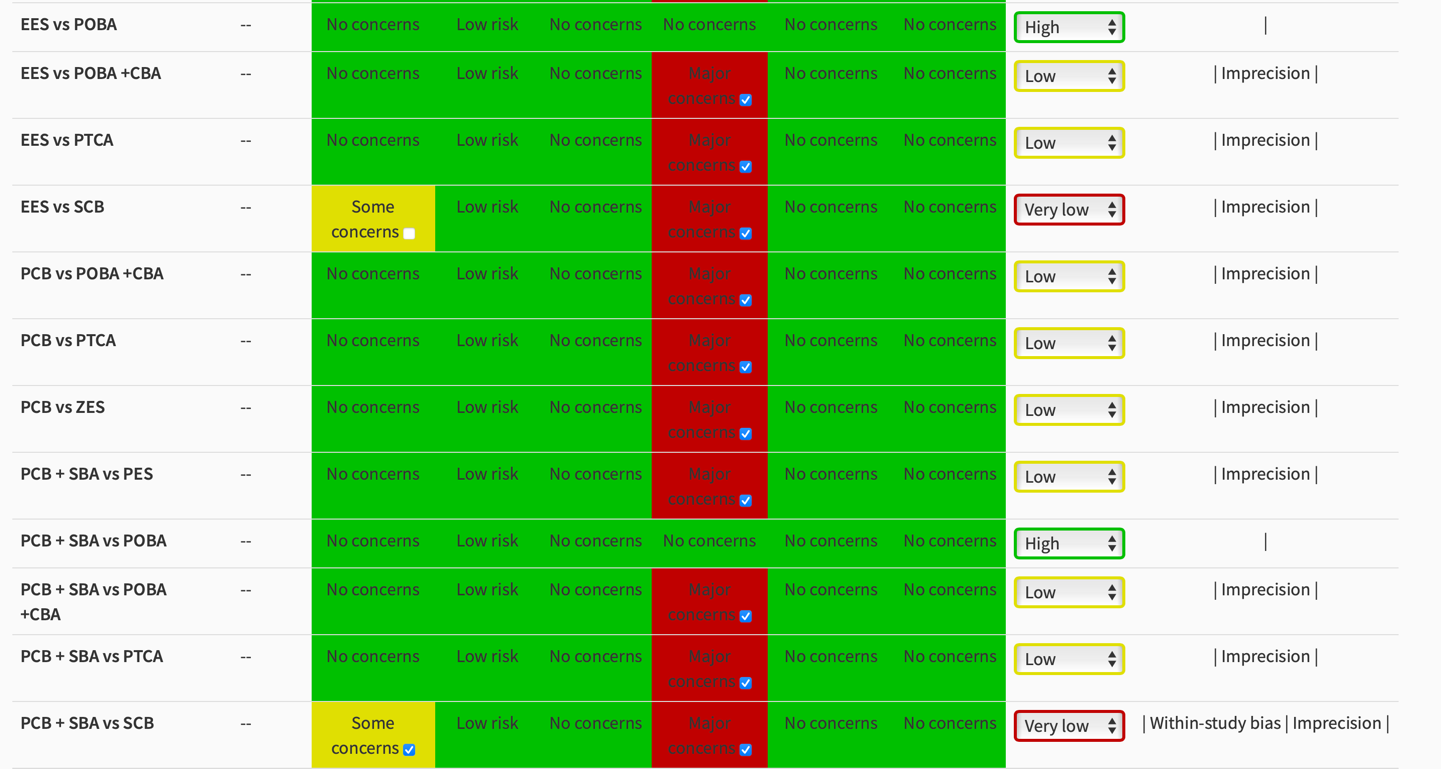

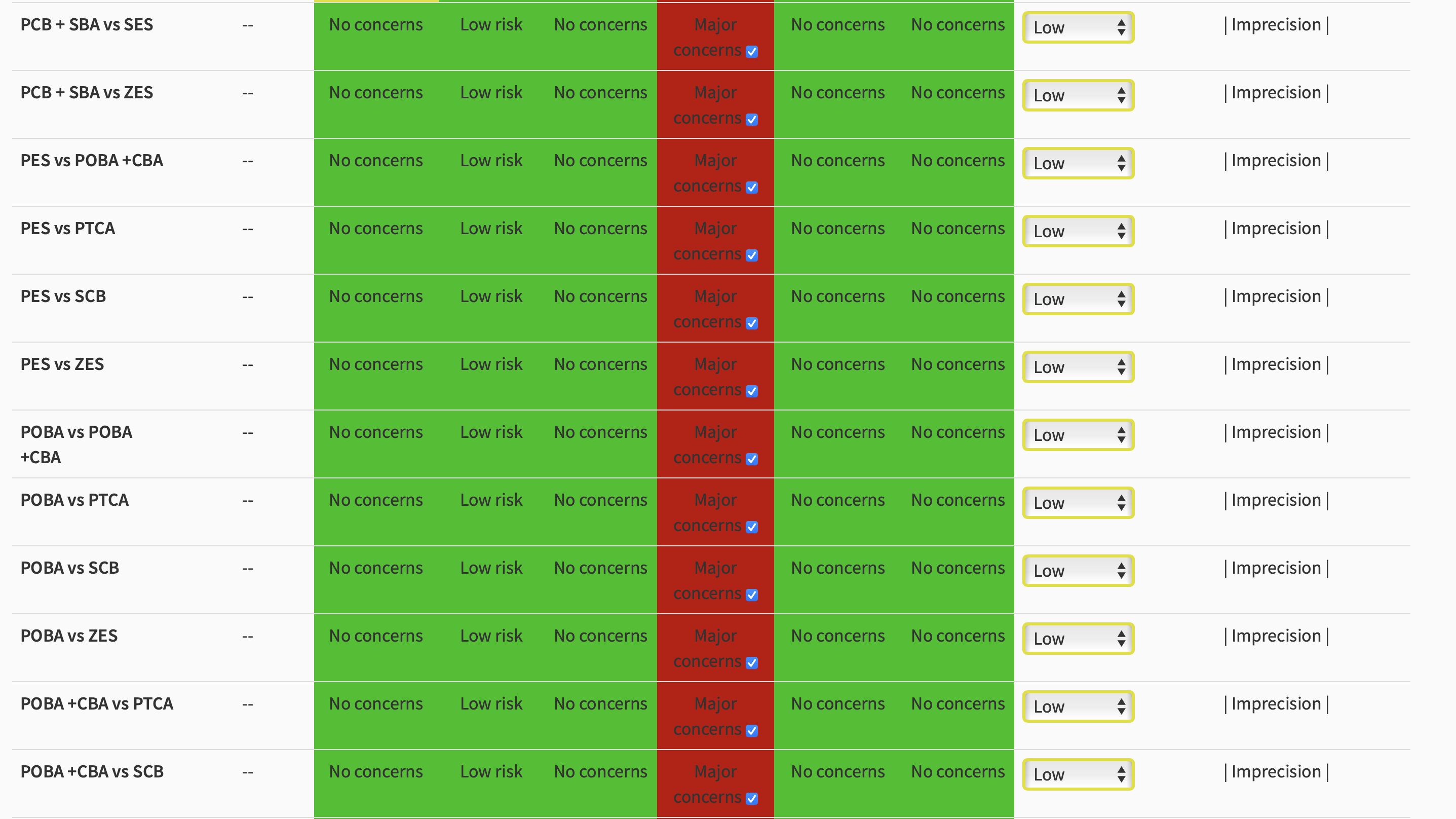

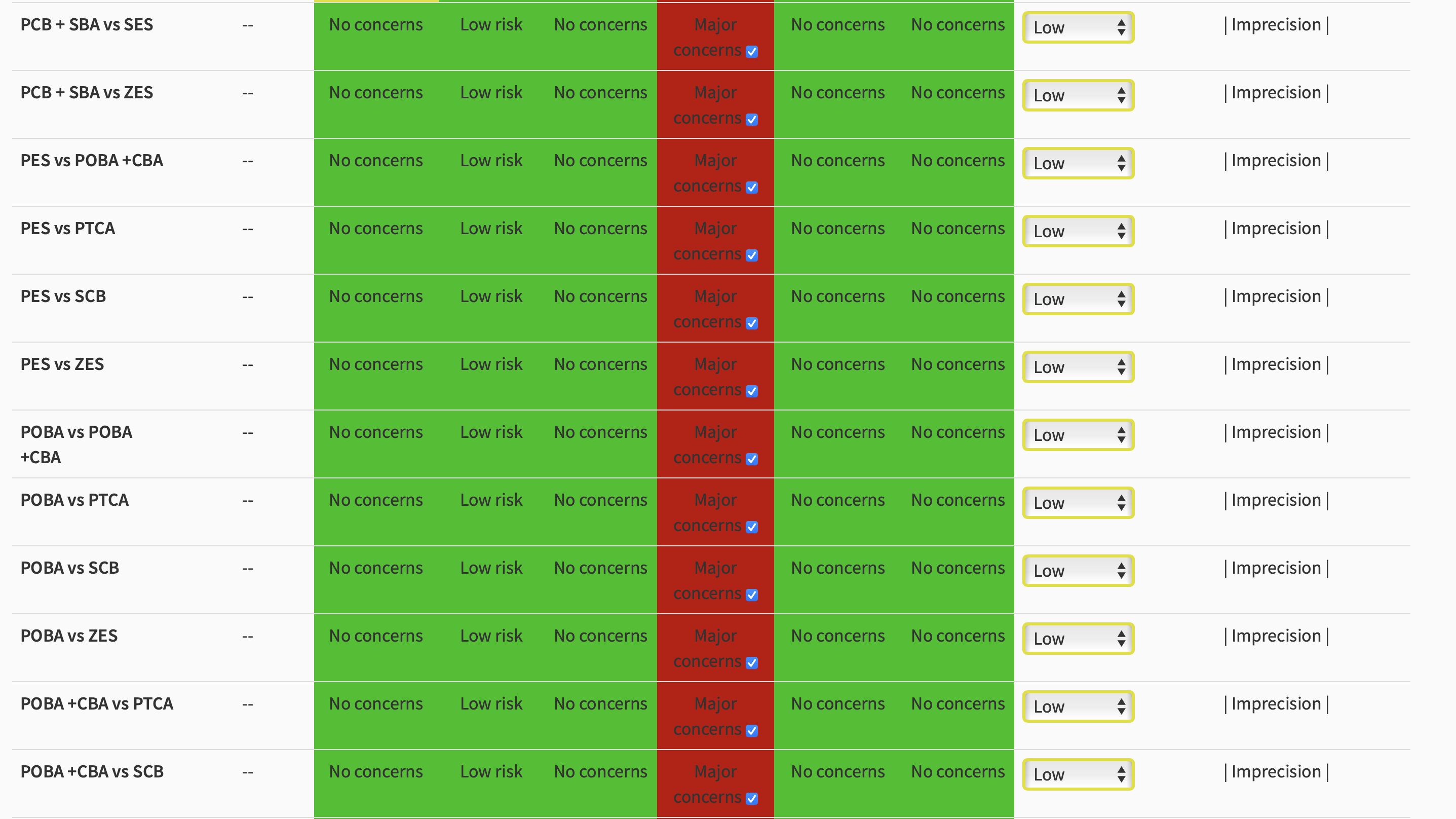

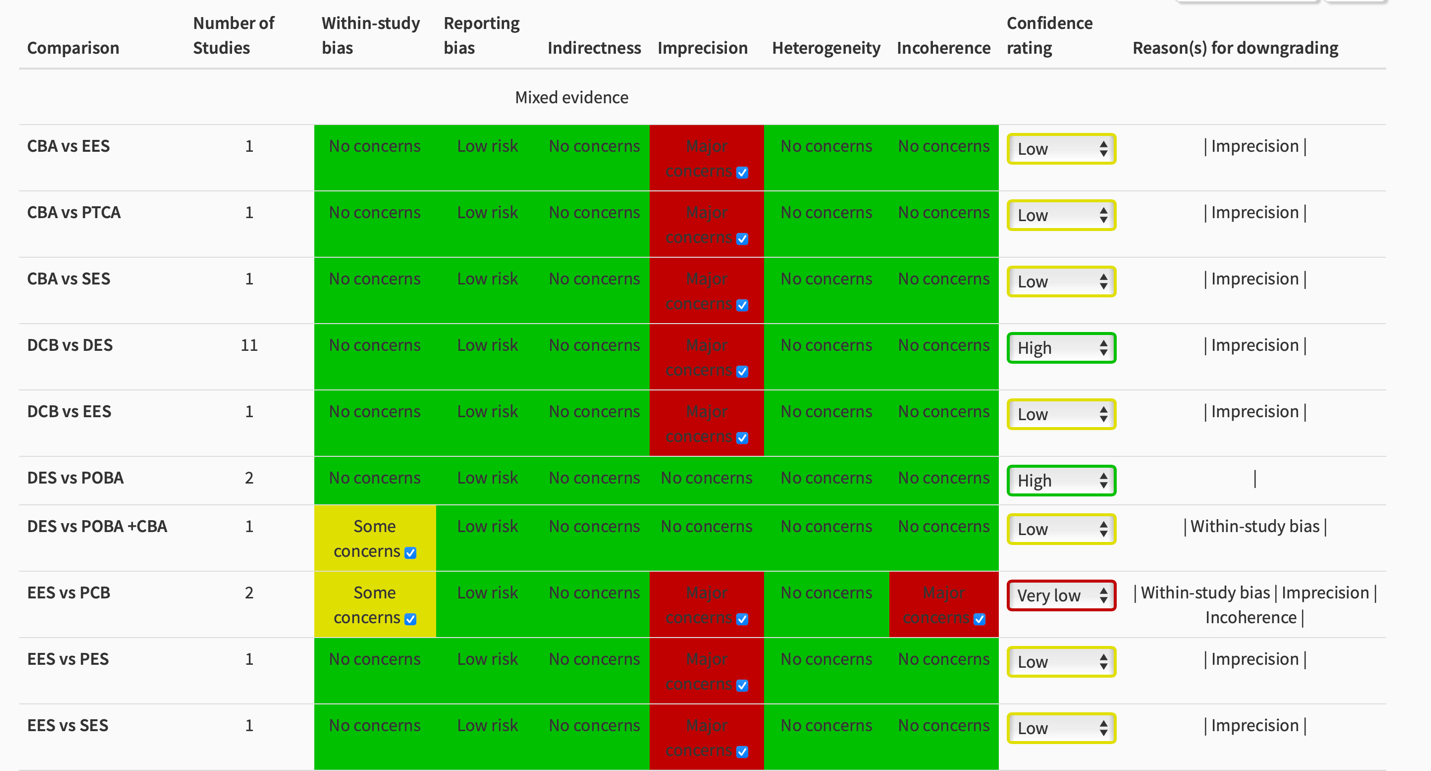

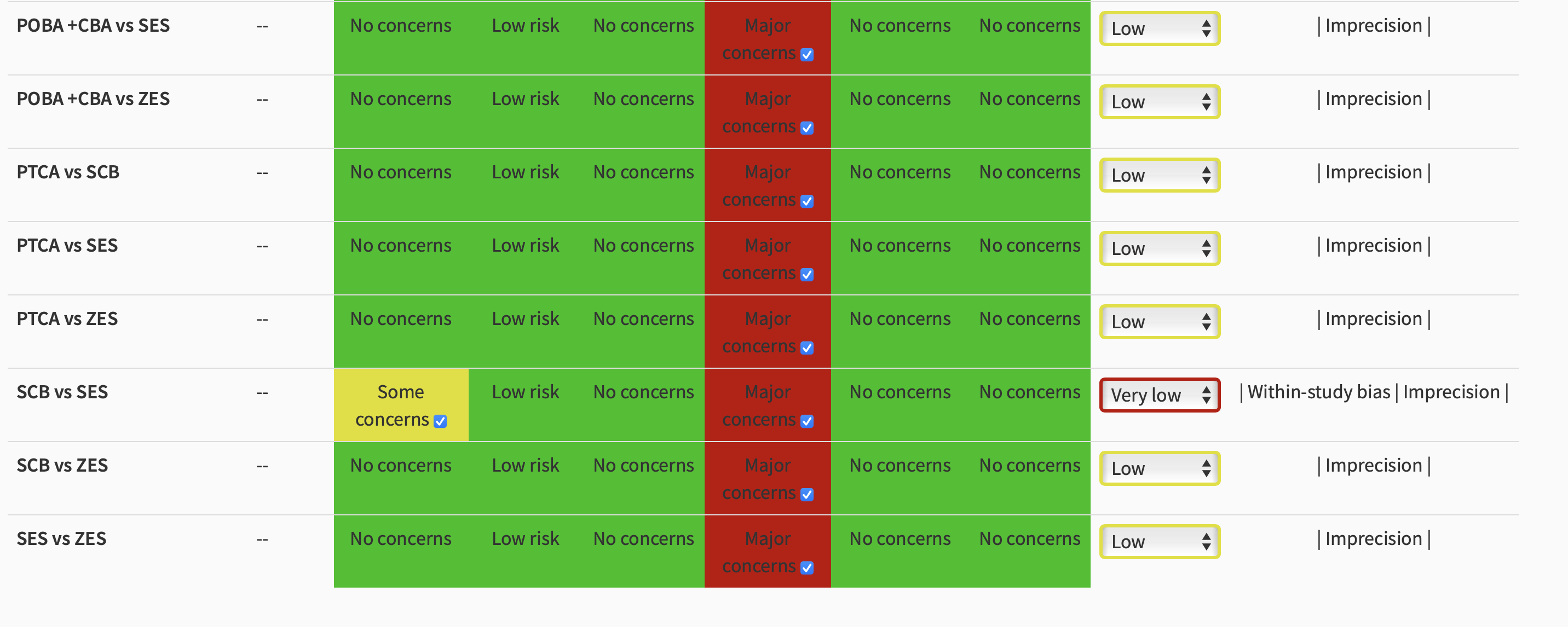


**Figure 2.** The evidence from the TLR analysis was graded and framed according to the Confidence in Network Meta-Analysis approach. The Confidence in Network Meta-Analysis considers the following domains: within-study bias, reporting bias, indirectness, imprecision, heterogeneity, and incoherence. Both direct and indirect evidence are represented separately.

**
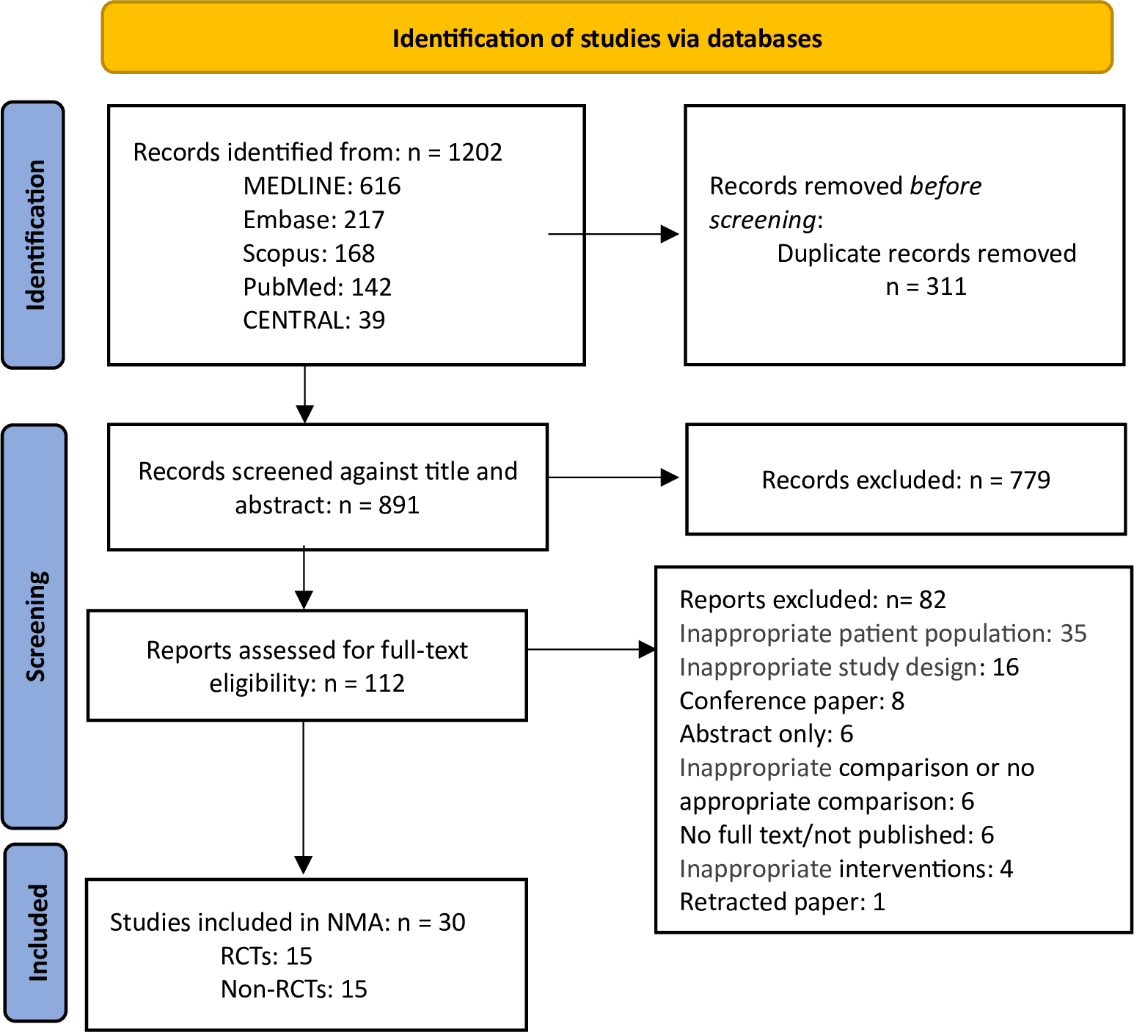
**

**Figure 3.** PRISMA flow diagram for relevant study search and screening


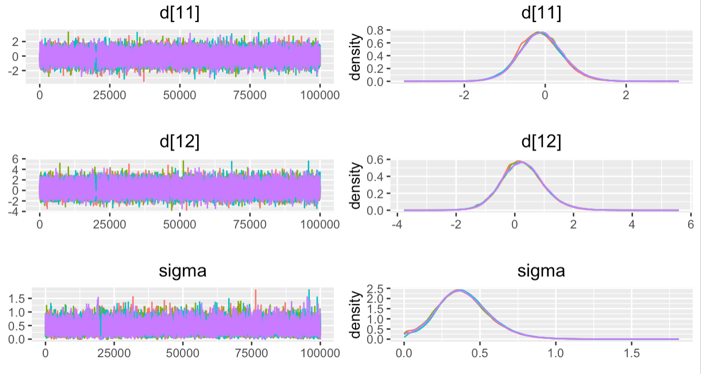

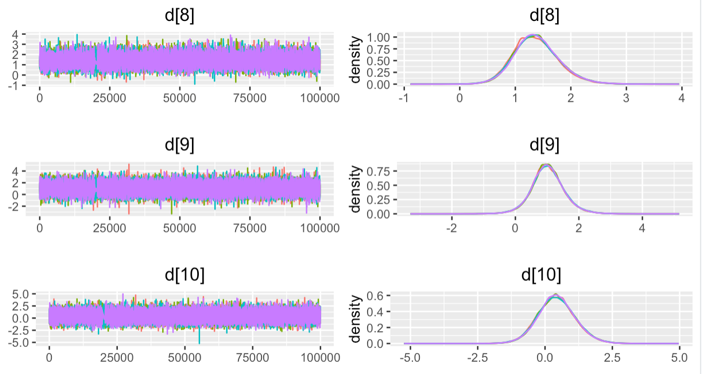

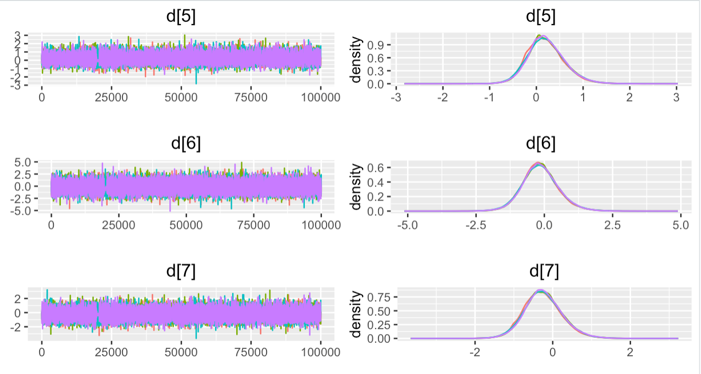

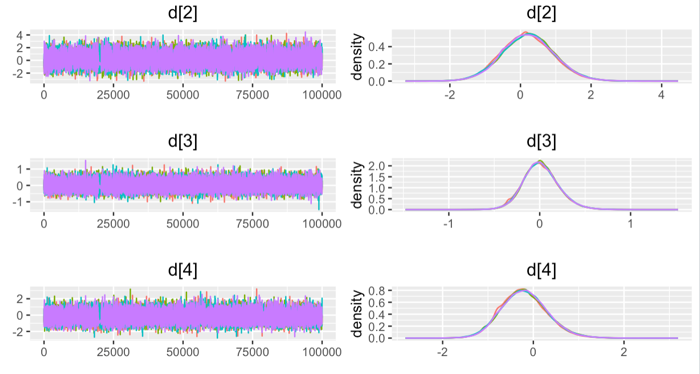


(A)

(B)


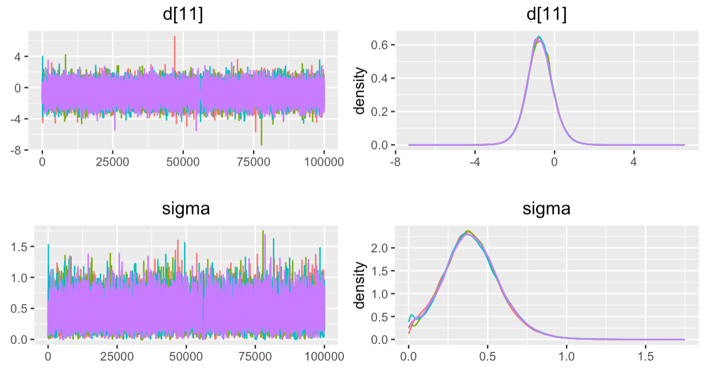

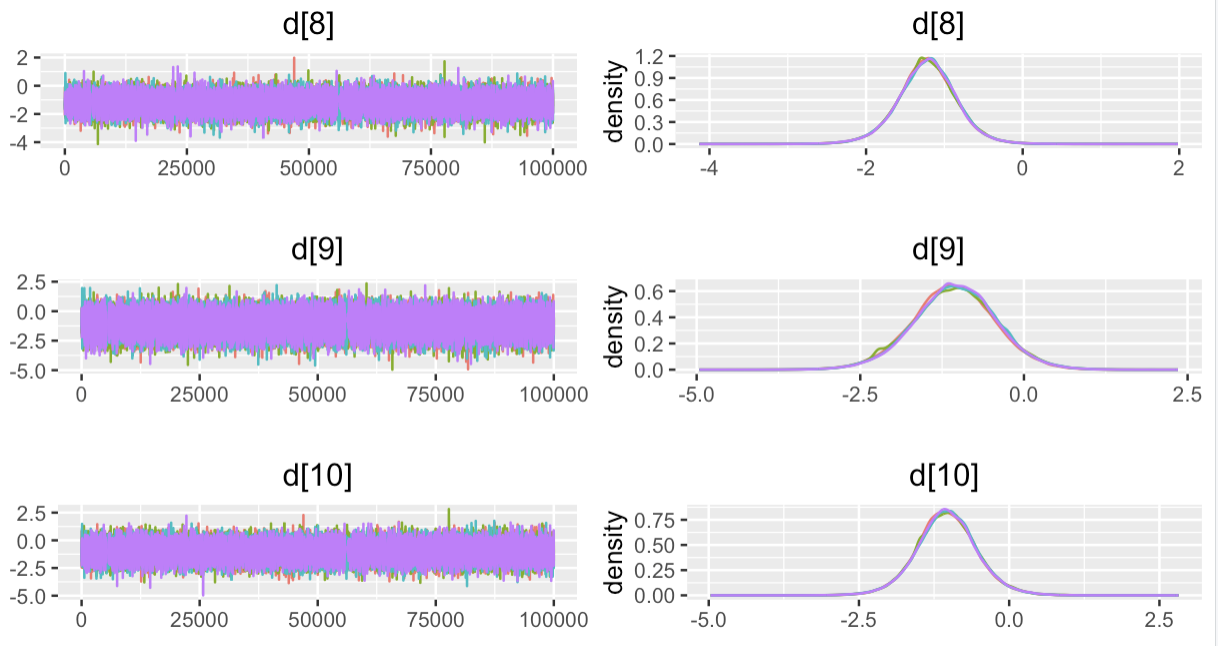

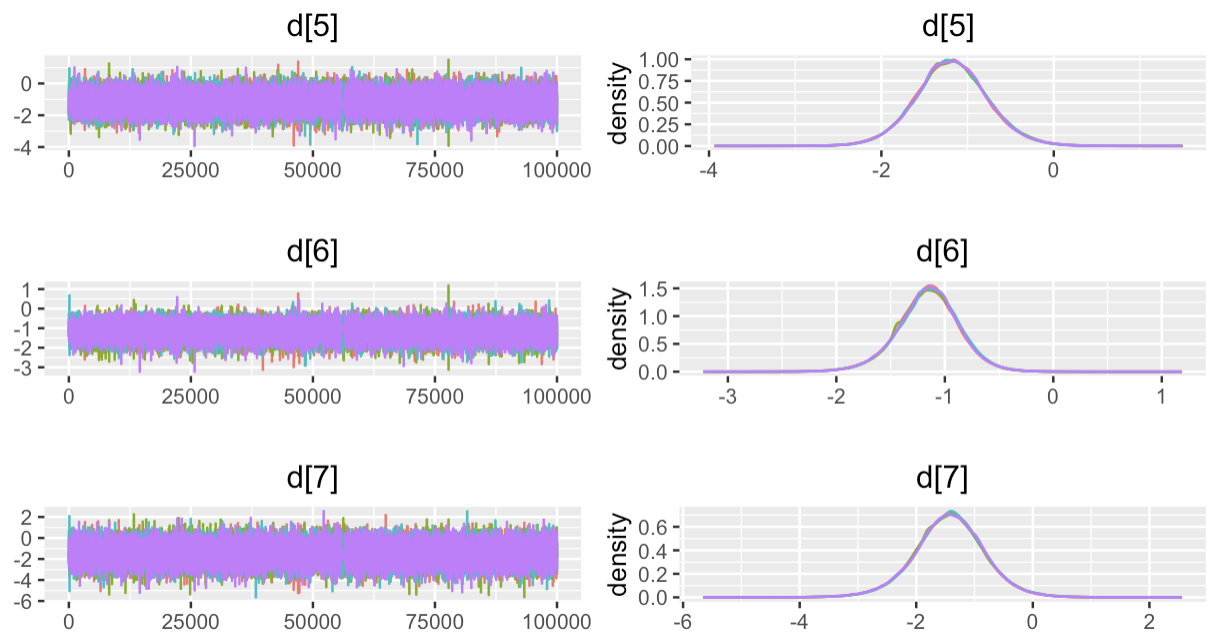

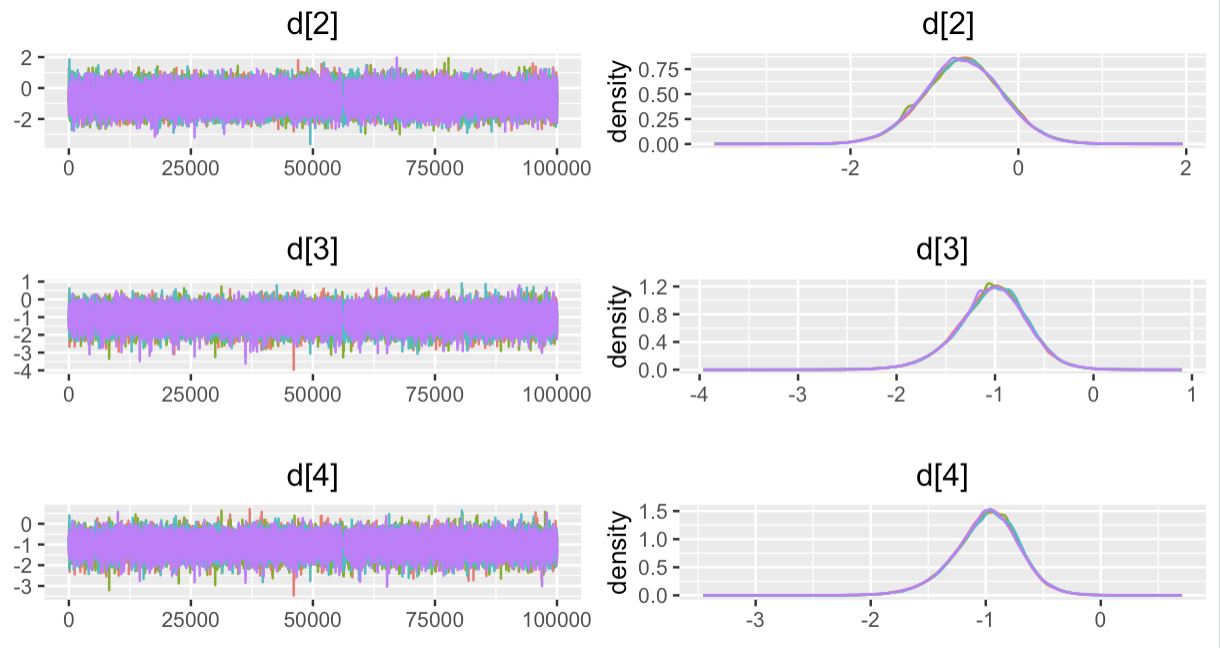

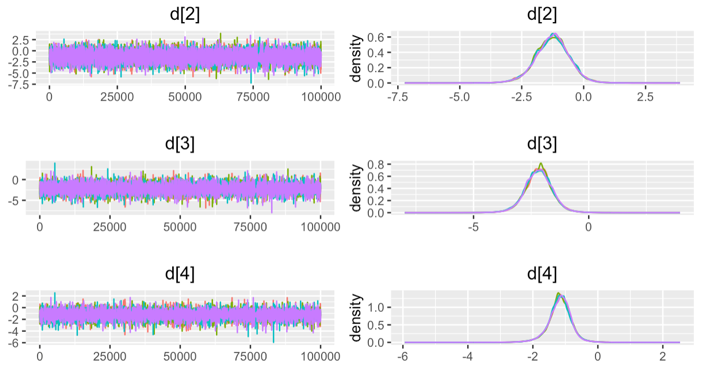

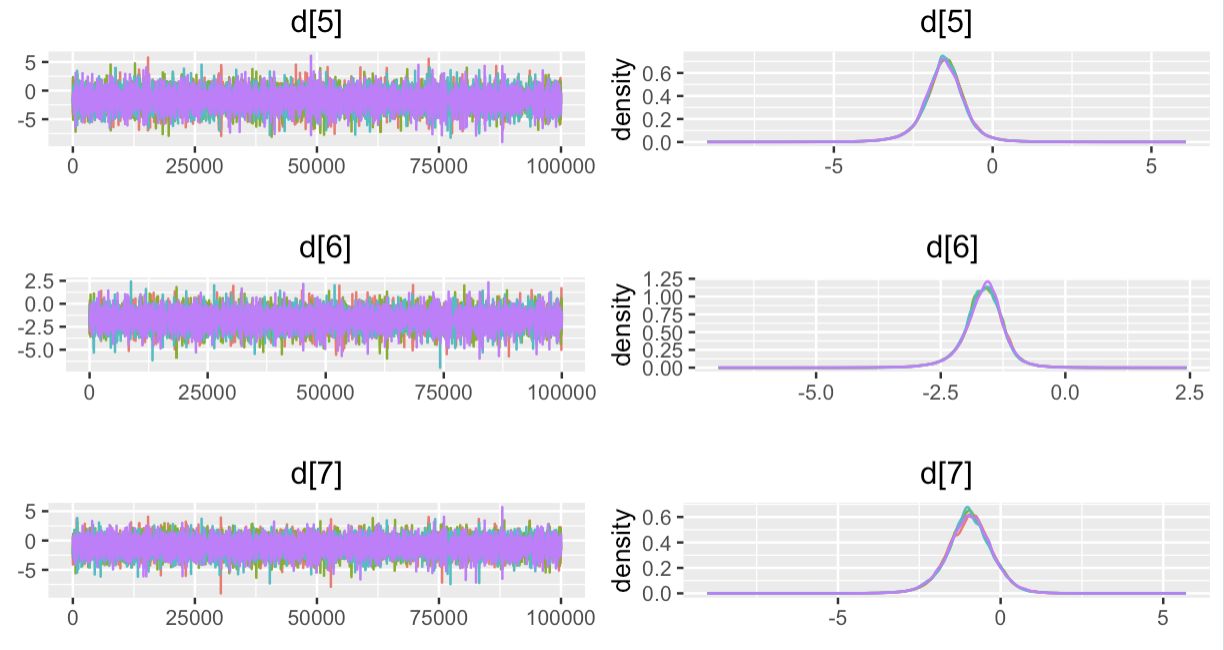

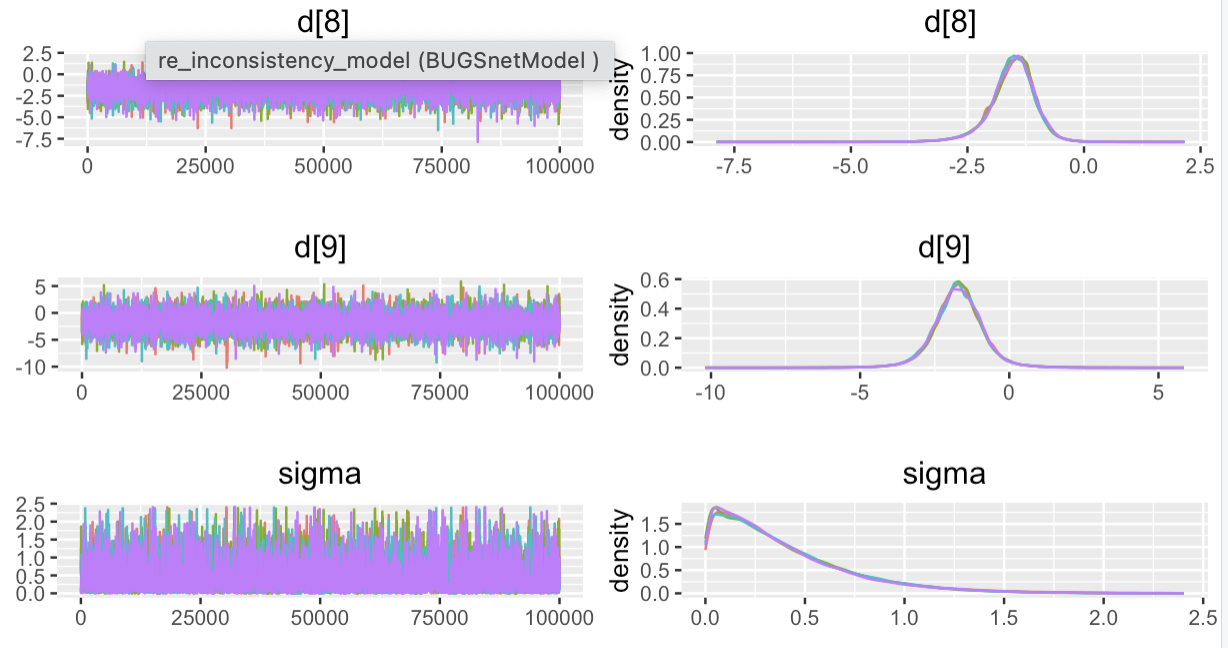


(C)

**(D)**


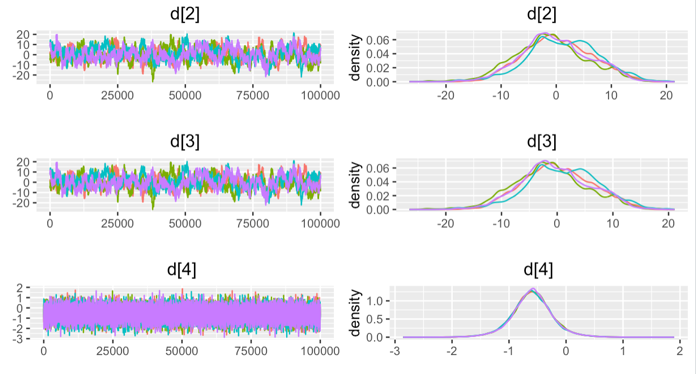

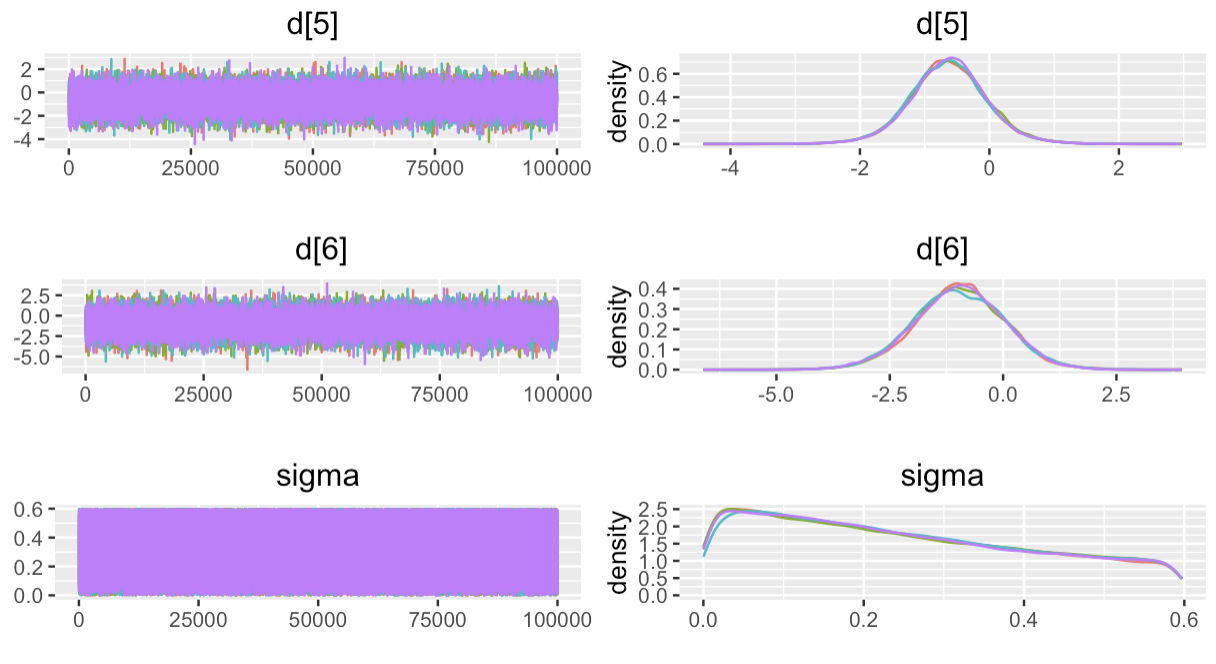

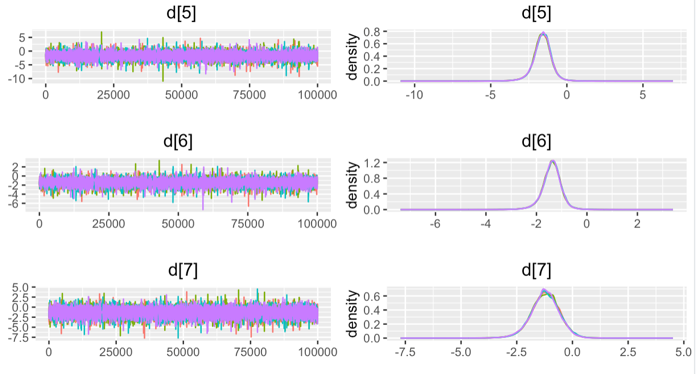

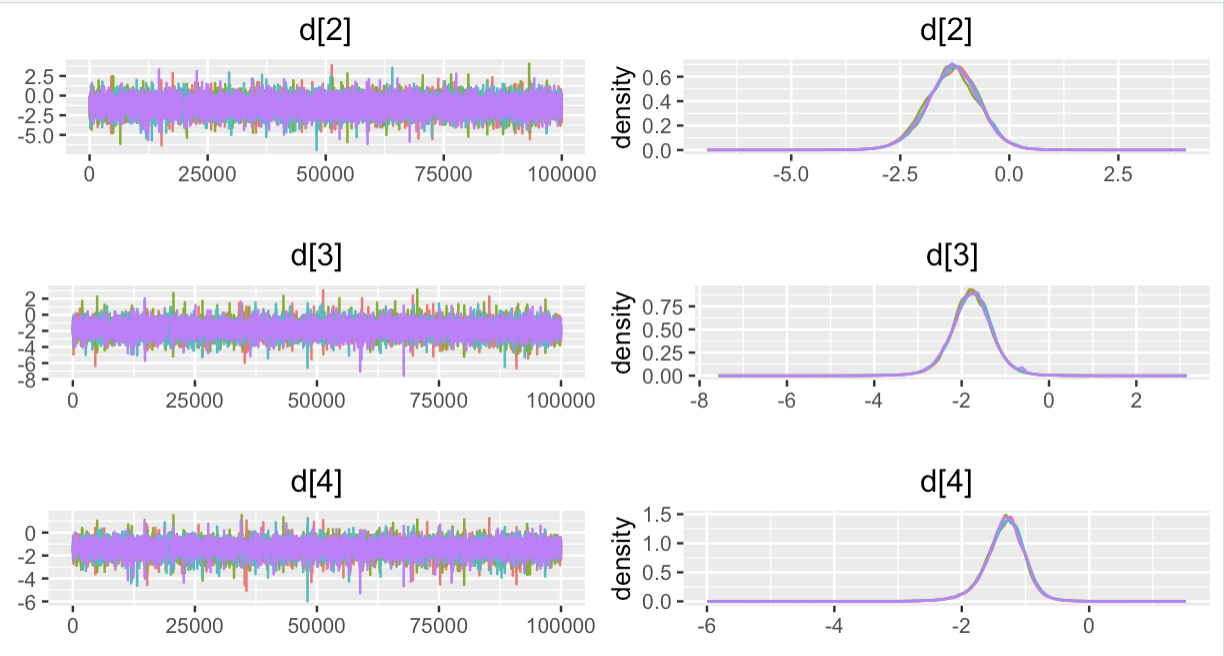

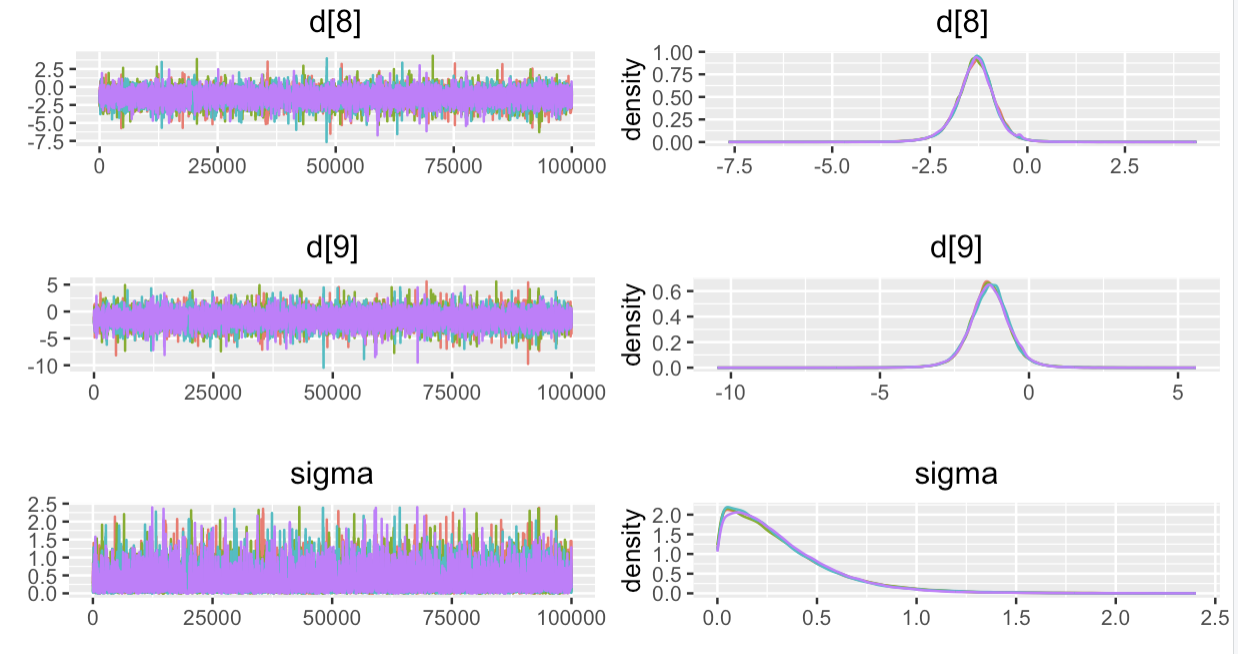


**(E)**

**(F)**


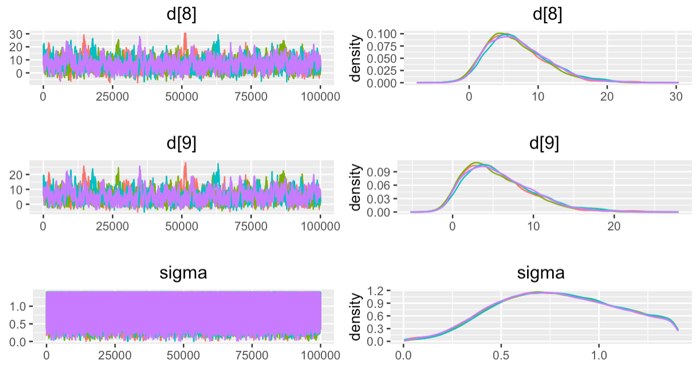

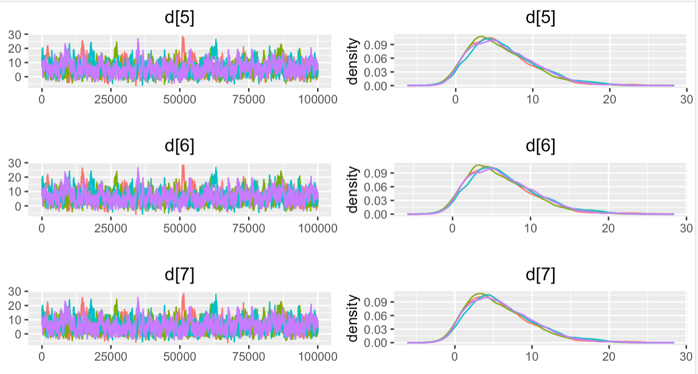

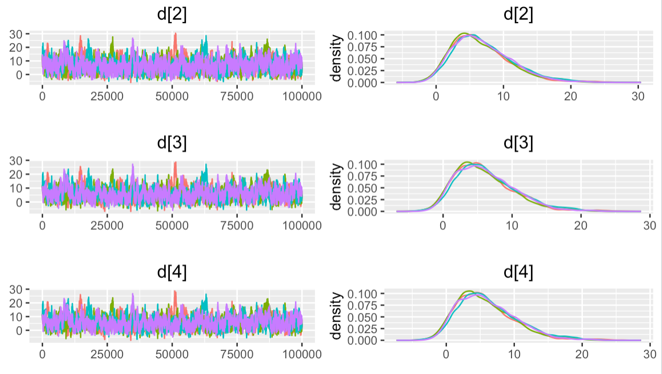

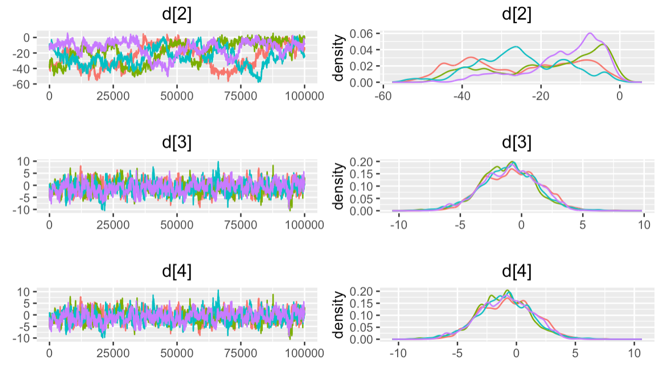

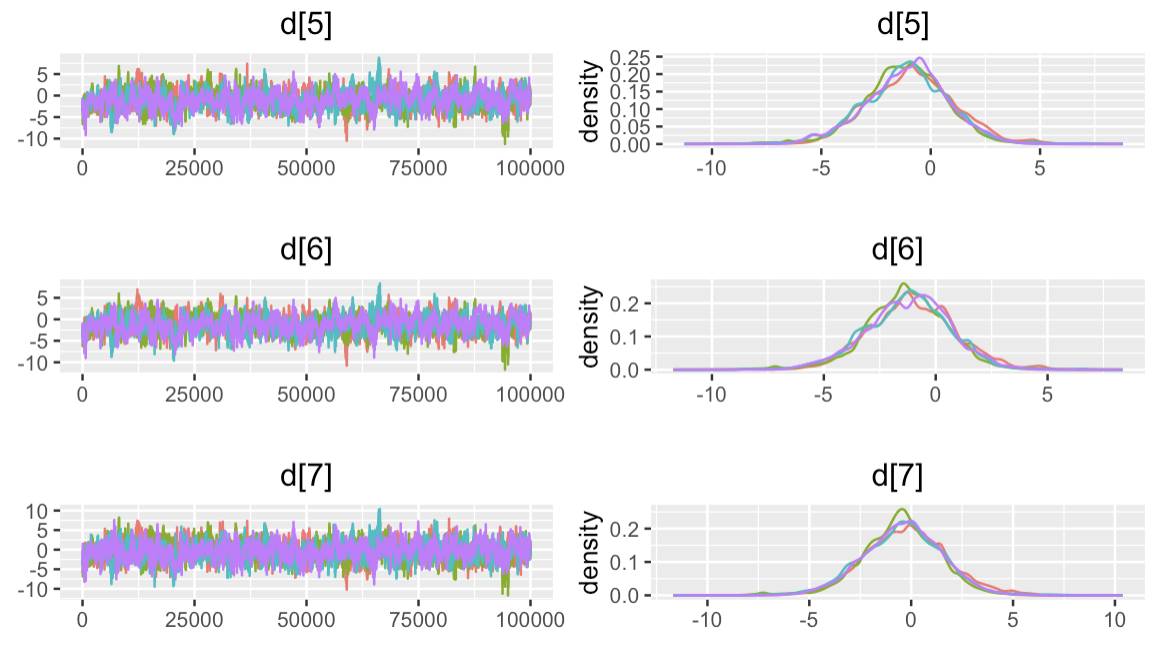

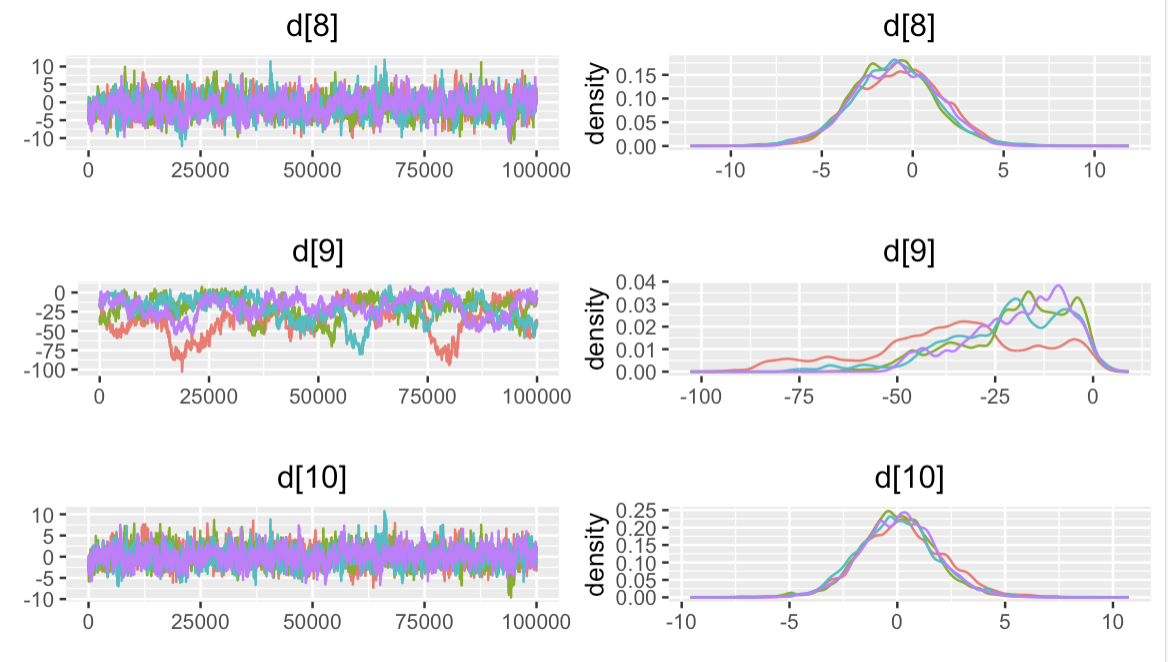

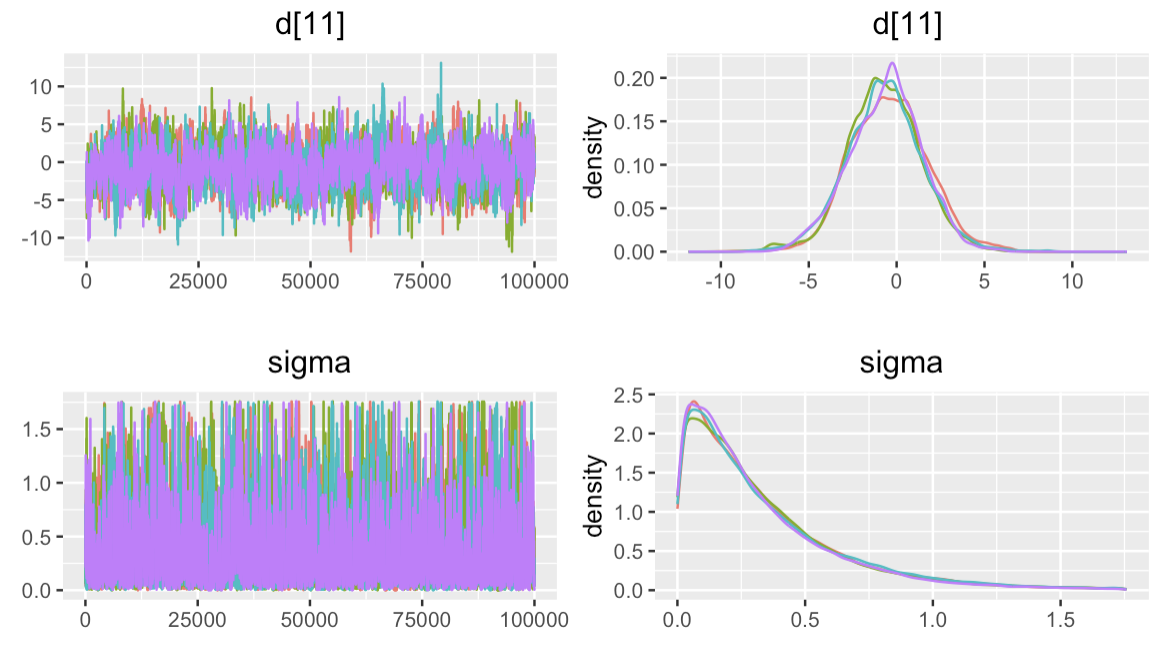


**(G)**


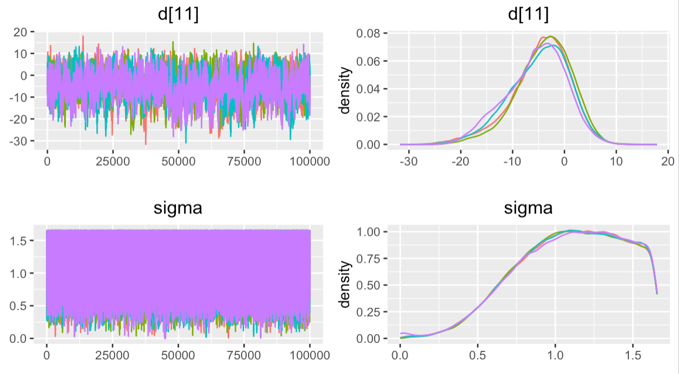

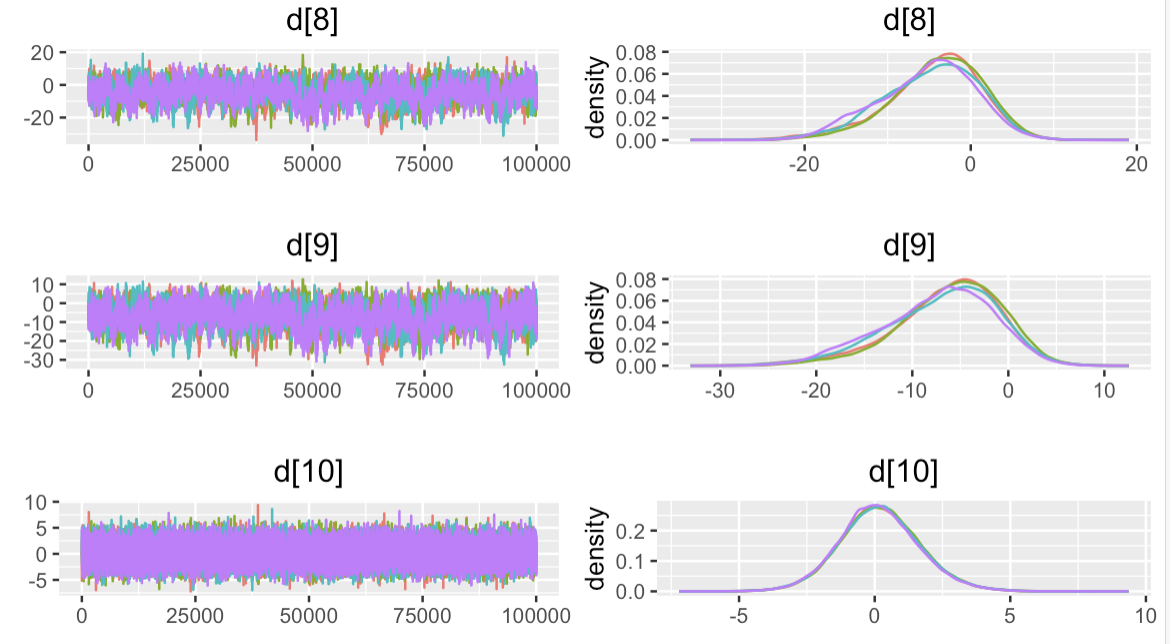

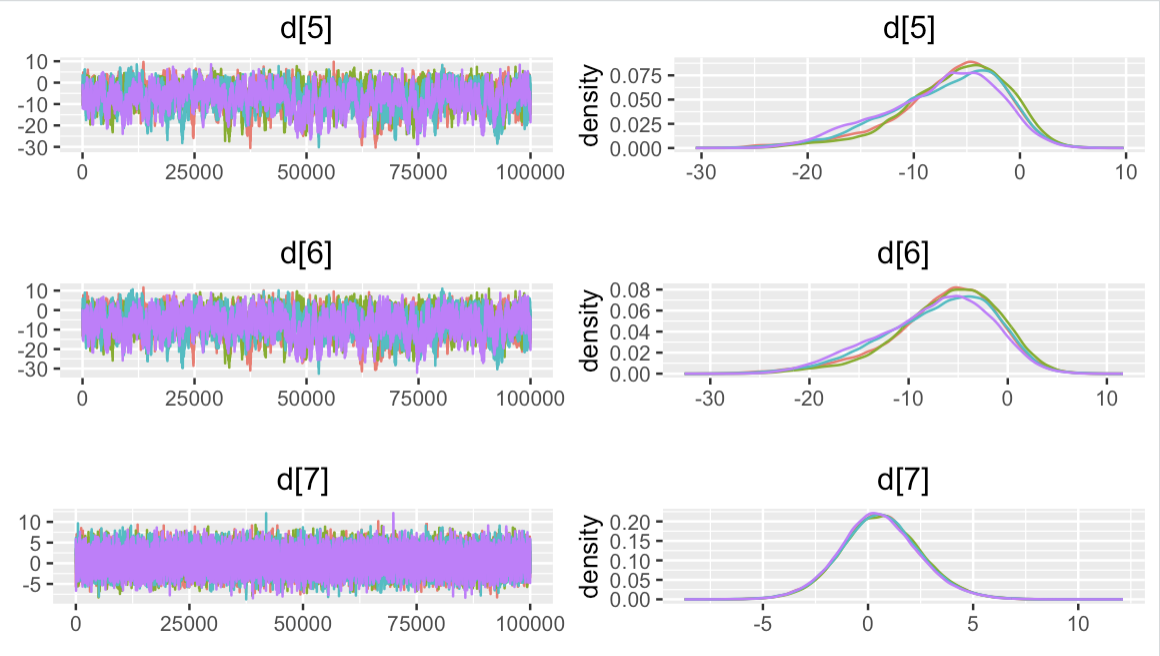

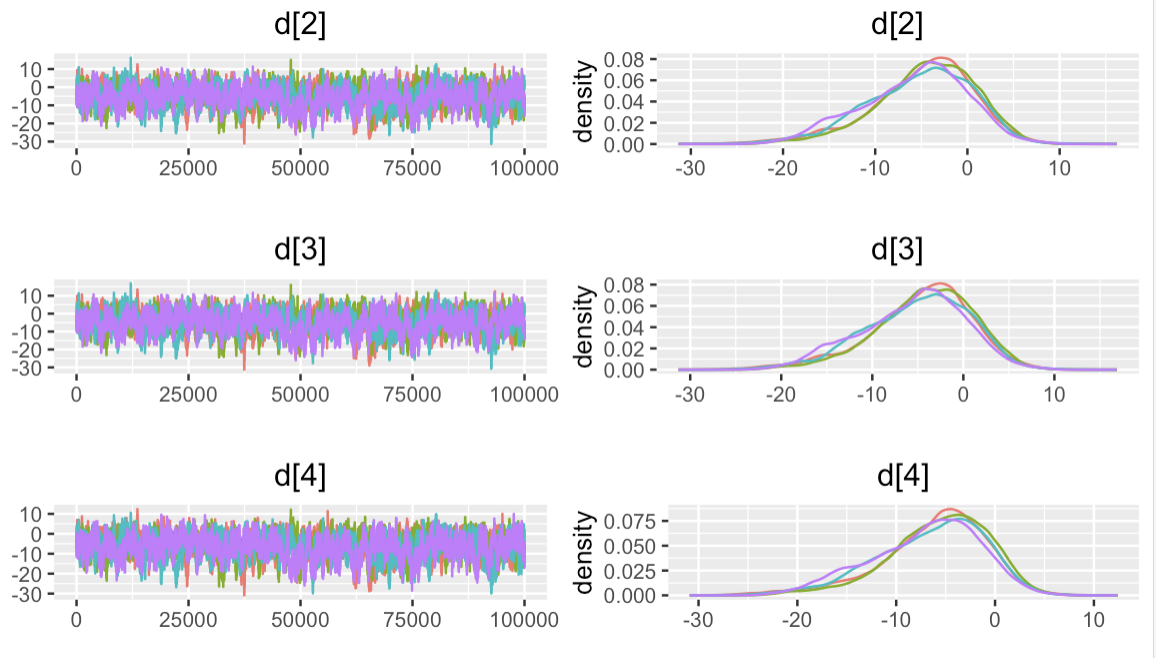


**(H)**


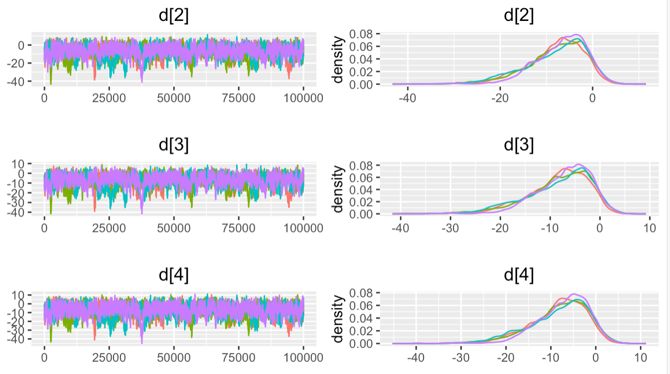

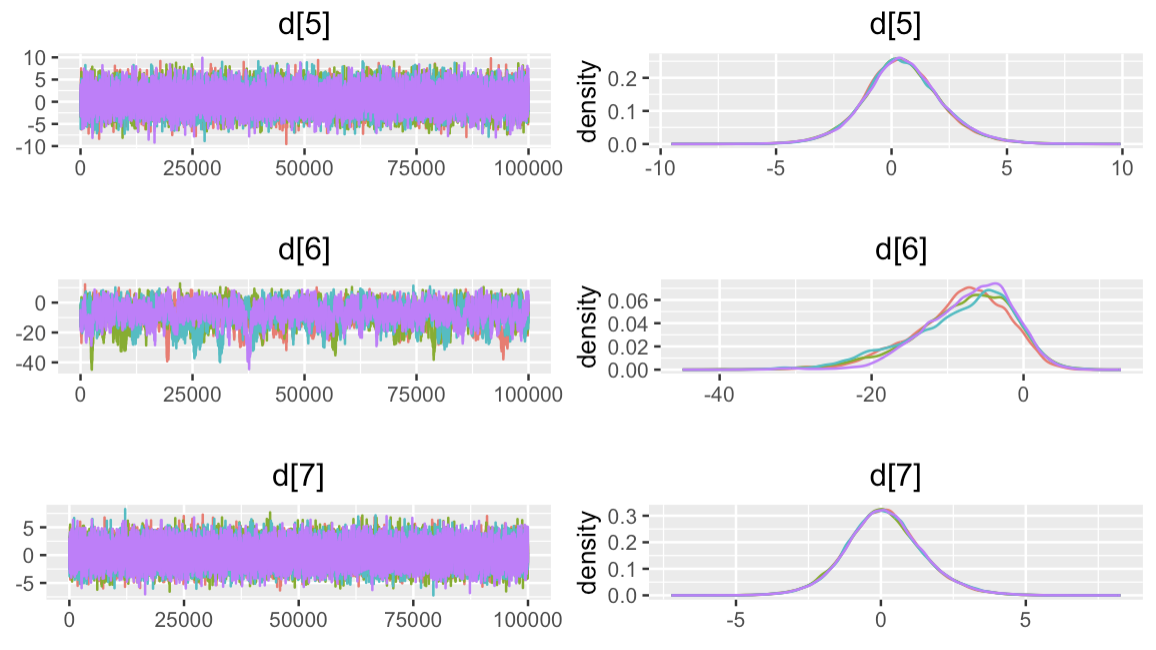

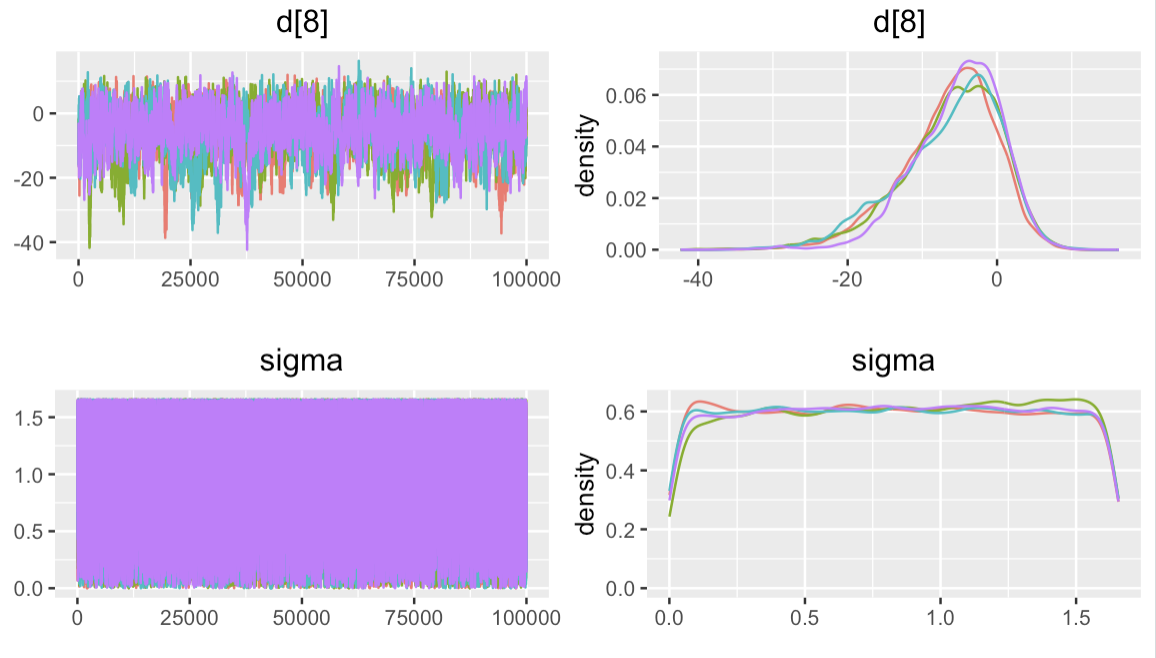


**(I)**


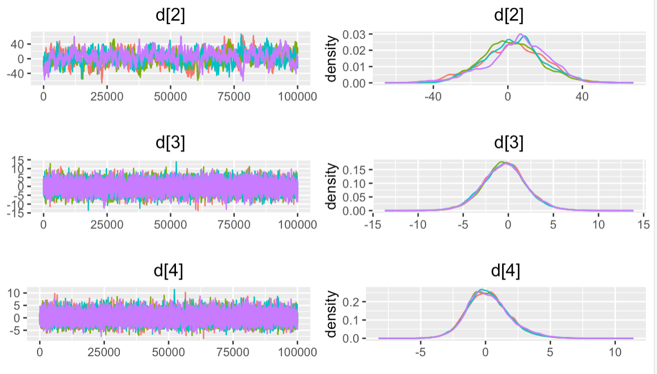

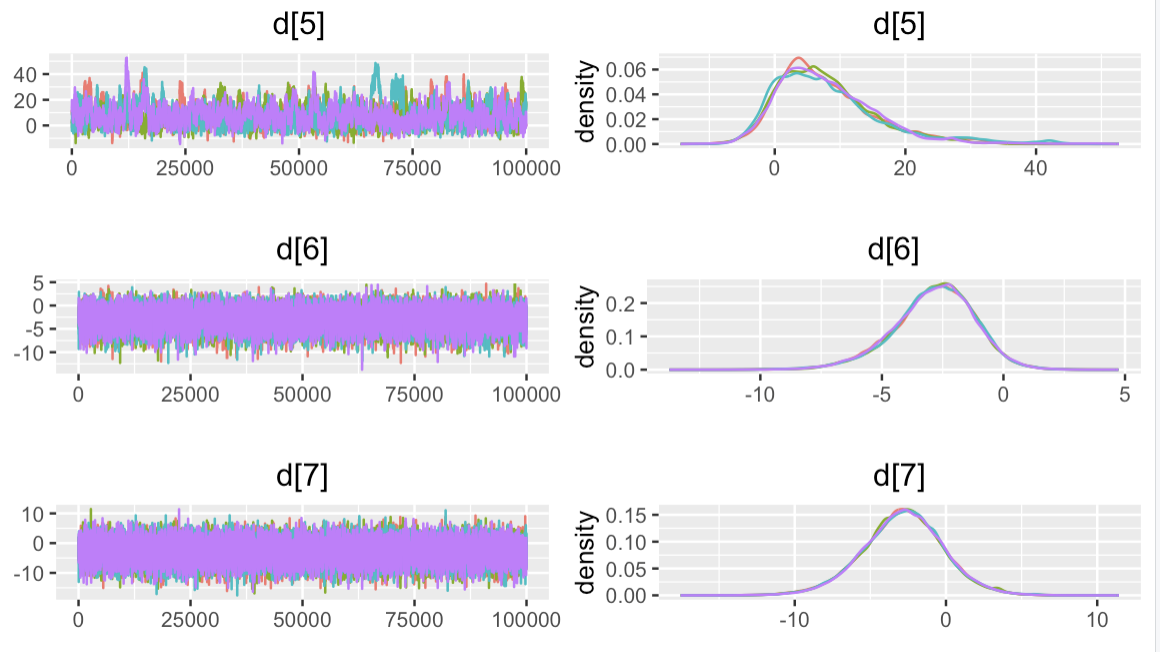

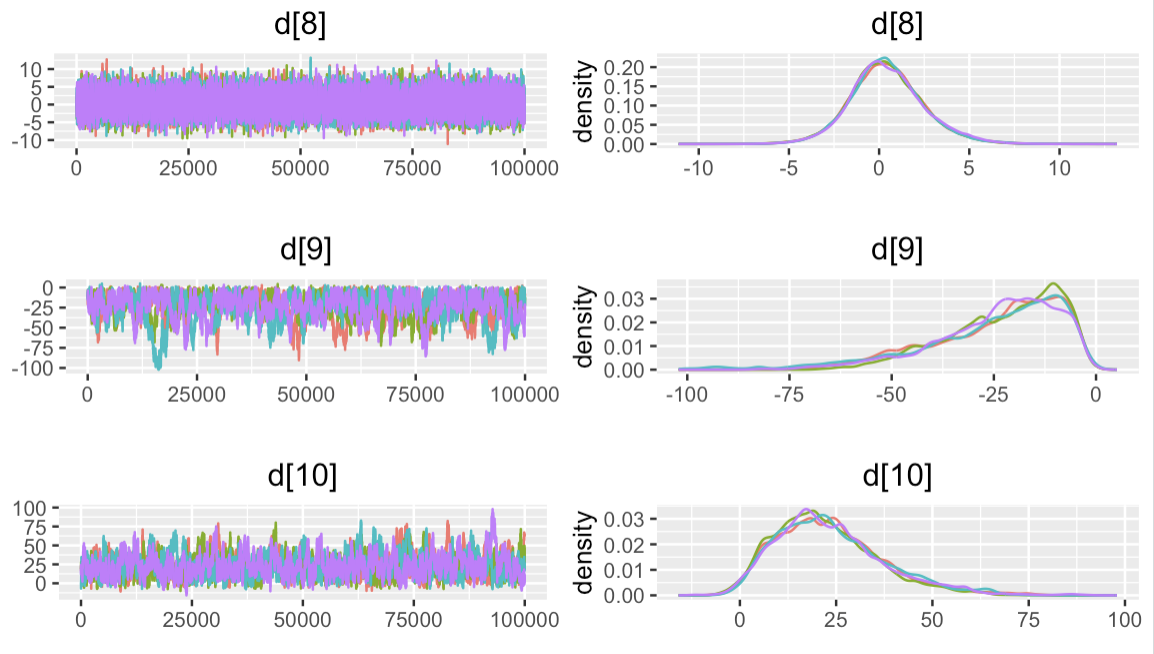

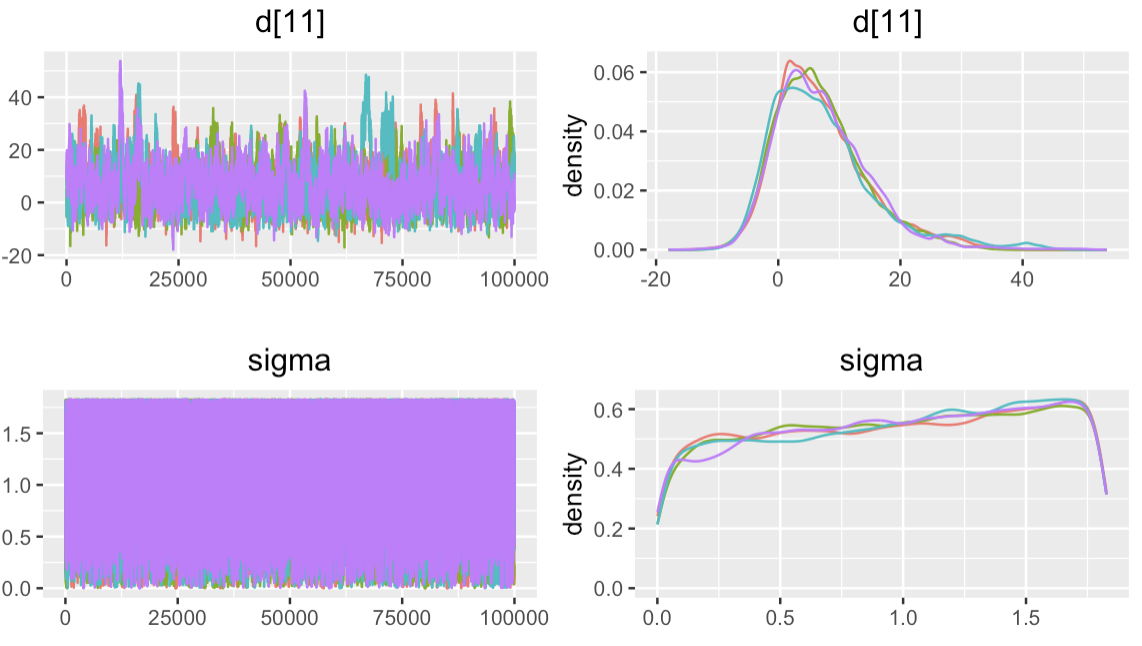


**(J)**

**Figure 4.** Trace Plot and Density Plot from Gelman-Rubin diagnostics (RE model): (A) For TLR; (B) For TLR (RCTs only); (C) For MACE; (D) For MACE (RCTs only); (E) For TLF; (F) For TVR; (G) For MI; (H) For all-cause death; (I) For Cardiac death; (J) For stent thrombosis

**TLF:**

A total of 5 studies reported the TLF events and assigned 2236 patients with DES-ISR to 6 interventions. Of the 7 possible pairwise comparisons, only 4 had direct comparison data. All 5 studies are two-arm studies. There were total of 410 TLF events, with none studies with zero events in any arms. The network plot was connected as shown in Figure 1(C).

In NMA forest plot, compared to POBA, none of the interventions shown to lower occurrence of TLF events statistically significantly compared to POBA [supplementary file, Figure 5(C)].

SUCRA values ranked SCB (64.16) highest and POBA (26.25) lowest. Following the SCB, PES (56.44), DES (56), PCB (55.98), and DCB (41.16) ranked successively in decreasing order [supplementary file, Figure 6(C), Table 2].

The heatmap of league plots of the network estimates confirmed the findings of the NMA forest plot and SUCRA plot. There were no significant differences between POBA and either interventions for the treatment of DES in regard of TLF events [supplementary file, Figure 7(E)]. Pairwise meta-analysis of pair of interventions shown in supplementary file, Figure 11.

**TVR:**

A total of 12 studies reported the TVR events and assigned 4537 patients with DES-ISR to 9 interventions. Of the 36 possible pairwise comparisons, only 11 had direct comparison data. Of 12 studies, 11 were two-arm while remaining 1 was multi-arm study. There were total of 671 TVR events, with one study with at least one zero event. The network plot was connected as shown in Figure 1(D).

In NMA forest plot, when compared to POBA, POBA+CBA (OR: 6.40; 95% CrI 0.05 to 17.40) might significantly increase the occurrence of the TVR events while no differences with other interventions [supplementary file, Figure 5(D)].

SUCRA values ranked POBA (96.32) highest and POBA+CBA (19.7) lowest. Following the POBA, SES (61.37), DES (56.53), PES (53.92), EES (50.34), DCB (46.67), PCB (37.36), and CBA (27.79) ranked in decreasing order [supplementary file, Figure 6(D), Table 2].

The heatmap of league plots of the network estimates confirmed the findings of the NMA forest plot and SUCRA plot. Compared to POBA+CBA, treatment of DES-ISR with POBA only might lower the occurrence of TVR events significantly [supplementary file, Figure 7(D)].

In pairwise meta-analysis of pair of interventions, the DES group had significantly lower occurrence of the TVR events compared with the DCB group. There was low-medium level of heterogeneity for for DES vs DCB (supplementary file, Figure 12).

**All cause death:**

A total of 14 studies reported the MI events and assigned 3590 patients with DES-ISR to 11 interventions. Of the 55 possible pairwise comparisons, only 10 had direct comparison data. All 14 were two-arm studies. There were total of 146 all-cause death events, with 2 studies with at least one zero event. The network plot was connected as shown in Figure 1(F).

In NMA forest plot, there were no significant differences between the other interventions and POBA as comparator in the RE model [supplementary file, Figure 5(F)].

SUCRA values ranked PCB (76.39) highest and PES (15.89) lowest in the RE model. Following the PCB, PCB+SBA (73.13), SCB (71.8), EES (71.17), and DCB (53.62) ranked in decreasing order. Remining interventions had SUCRA values below 50 ([supplementary file, Figure 6(F), Table 2].

The heatmap of league plots of the network estimates confirmed the findings of the NMA forest plot. There were no significant differences among the different interventions [supplementary file, Figure 7(F)].

In pairwise meta-analysis of pair of interventions, there were no differences between DES vs DCB. There was high level of heterogeneity for DES vs DCB [supplementary file, Figure 14].

**Cardiac death**:

A total of 10 studies reported the cardiac death events and assigned 4014 patients with DES-ISR to 6 interventions. Of the 15 possible pairwise comparisons, only 5 had direct comparison data. All 10 studies were two-arm studies. There were total of 73 cardiac death events, with 4 studies with at least one zero event. The network plot was connected as shown in Figure 1(G).

In NMA forest plot, there was no significant differences between POBA and other interventions for the treatment of DES-ISR in regard of the cardiac death risk [supplementary file, Figure 5(G)

SUCRA values ranked PCB (83.74) highest and DES (7.82; FE model: 2.81) lowest. Following the PCB, EES (81.93), SES (61.73), POBA (48.16), and DCB (16.64) ranked in decreasing order in both the models (death [supplementary file, Figure 6(G), Table 2].

The heatmap of league plots of the network estimates confirmed the findings of the NMA forest plot and SUCRA plot. Compared to DCB and DES, treatment of the DES-ISR with PCB and EES might significantly lower the occurrence of cardiac death events [supplementary file, Figure 7(G)]. Pairwise meta-analysis of pair of interventions shown in supplementary file, Figure 15.

**Stent thrombosis:**

A total of 12 studies reported the stent thrombosis events and assigned 2737 patients with DES-ISR to 11 interventions. Of the 55 possible pairwise comparisons, only 12 had direct comparison data. Of 12 studies, 11 were two-arm studies while 1 was multi-arm study. There were total of 44 stent thrombosis events, with 7 studies with at least one zero event. The network plot was connected as shown in Figure 1(H).

In NMA forest plot, compared with POBA, the treatment of DES-ISR with SCB (OR -20.60, 95% CrI -61.35 to -3.77) might significantly lower the occurrence of stent thrombosis but its treatment with SES (OR 21.33, 95% CrI 1.67 to 55.93) might significantly increase the occurrence of stent thrombosis [supplementary file, Figure 5(H)].

SUCRA values ranked SCB (98.53) highest and SES (1.92) lowest. Following the SCB, PCB (77.04), PES (73.78), DCB (56.53), POBA (49.85), DES (49.37), POBA+CBA (46.55) and CBA (46.01) ranked in decreasing order. Other remaining interventions had SUCRA values less than 45 [supplementary file, Figure 6(H); Table 2]

The heatmap of league plots of the network estimates confirmed the findings of the NMA forest plot and SUCRA plot. The treatment of the DES-ISR with SCB might significantly lower the occurrence of stent thrombosis events compared with PCB, PES, DCB, DES, POBA, POBA+CBA, ZES, EES and SES. Similarly, treatment of the DES-ISR with PCB, PES, DCB, POBA, DES and POBA+CBA might significantly lower the occurrence of stent thrombosis events compared with SES [supplementary file, Figure 7(H)].

In pairwise meta-analysis of pair of interventions, there was no significant differences between DES vs DCB and DES vs POBA in regards of the stent thrombosis events. There was no heterogeneity for DES vs DCB and DES vs POBA death (supplementary file, Figure 16).

**
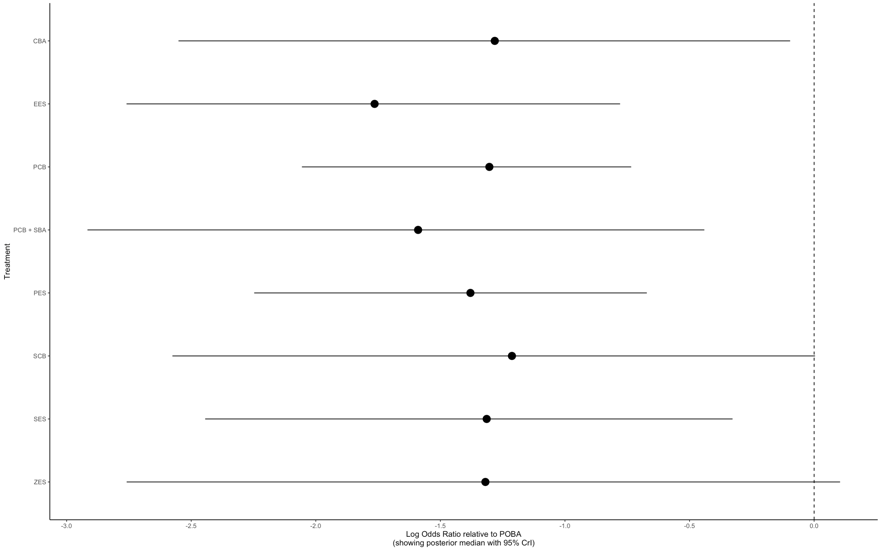
**
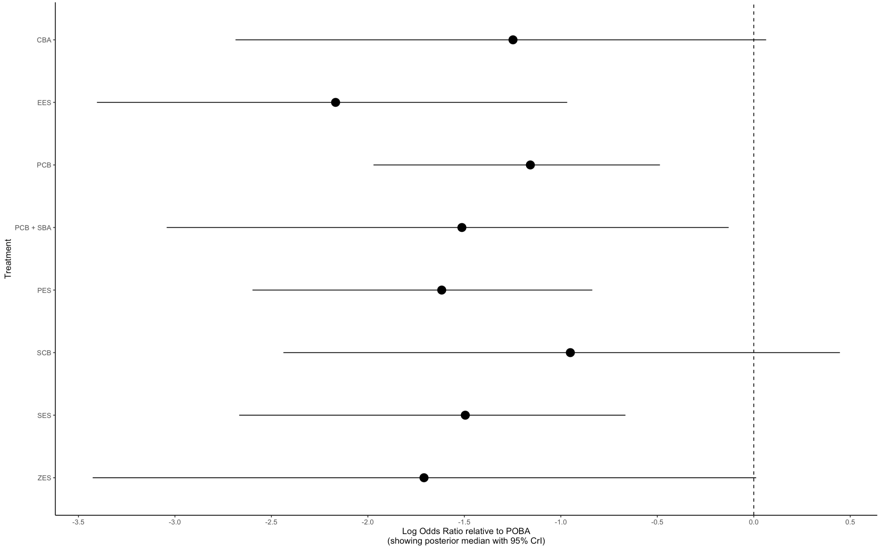


**
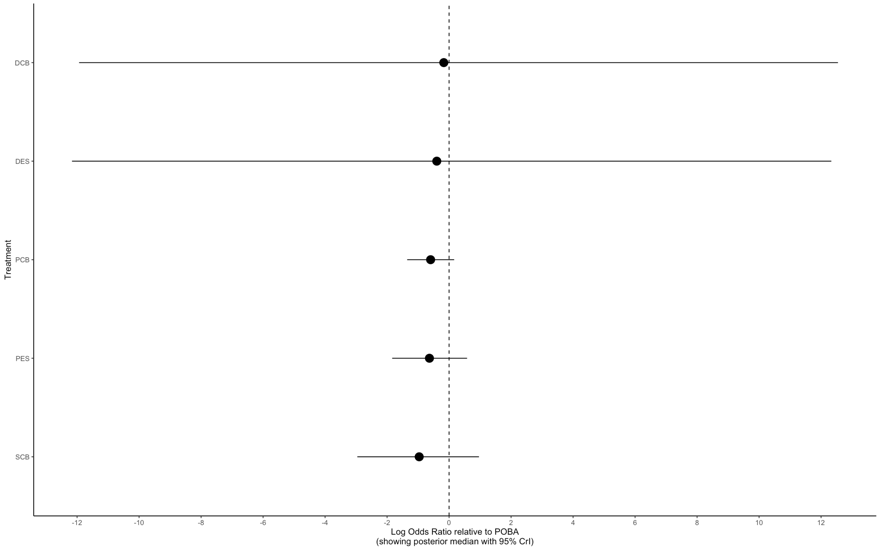
**
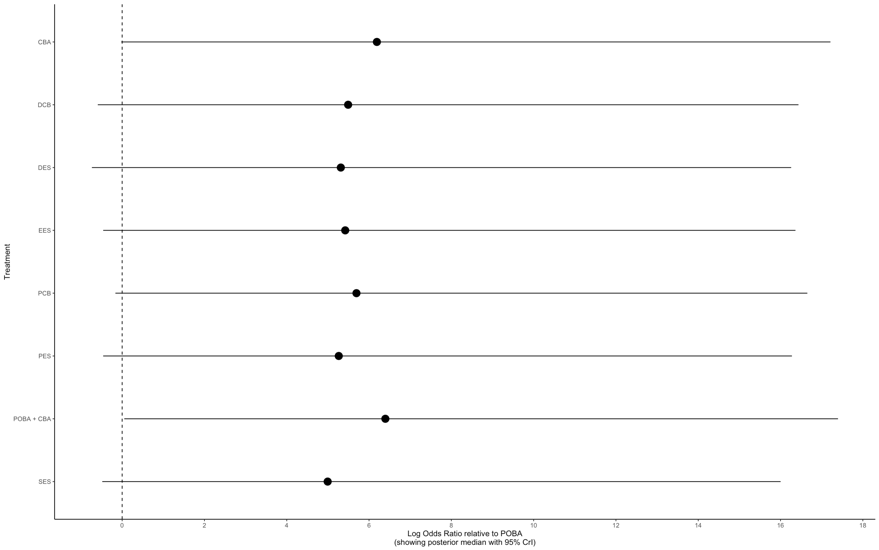
 **(A) (B)**

**(C) (D)**

**
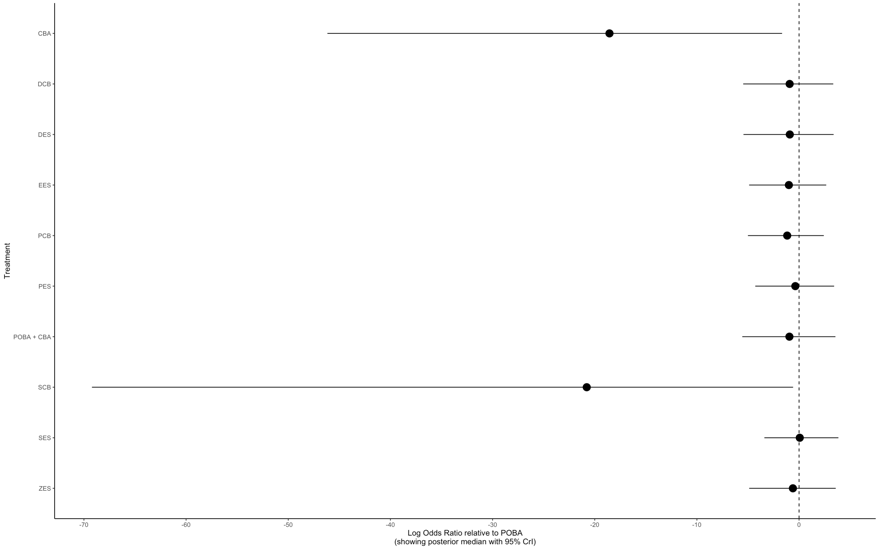
**
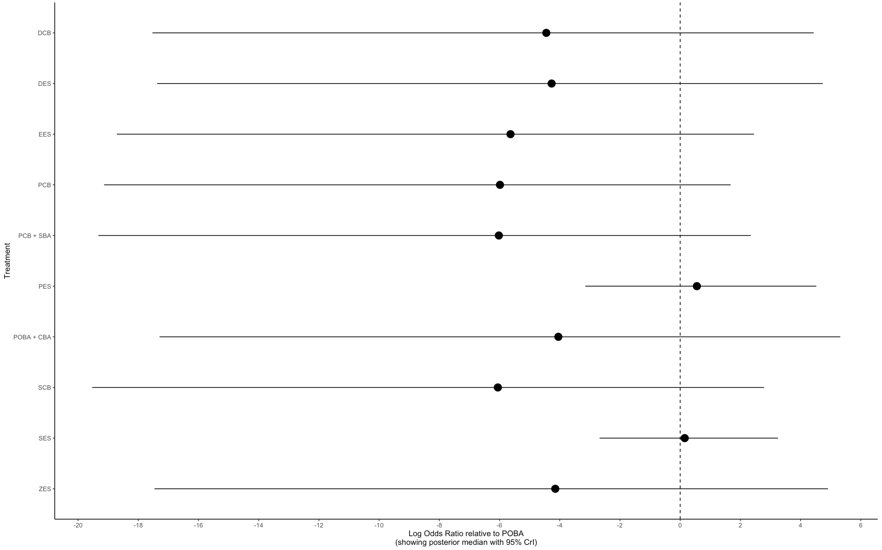


**(E) (F)**

**
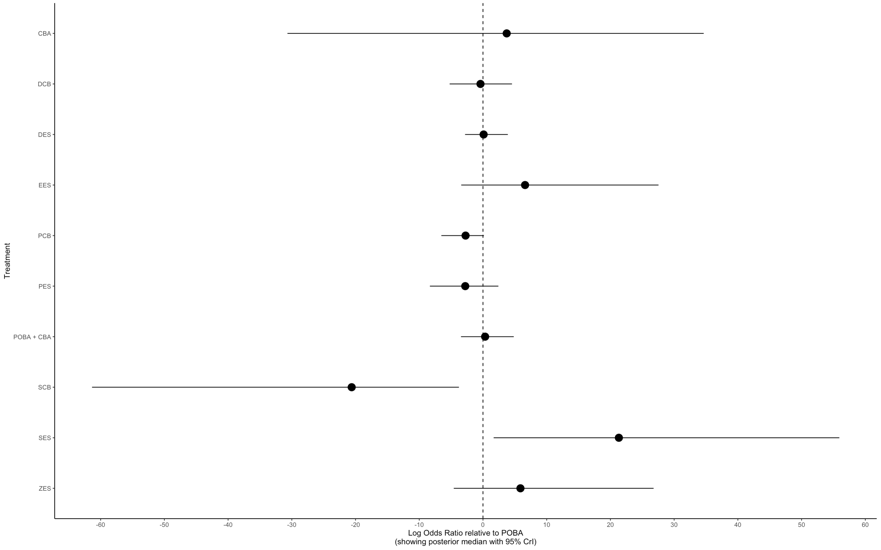

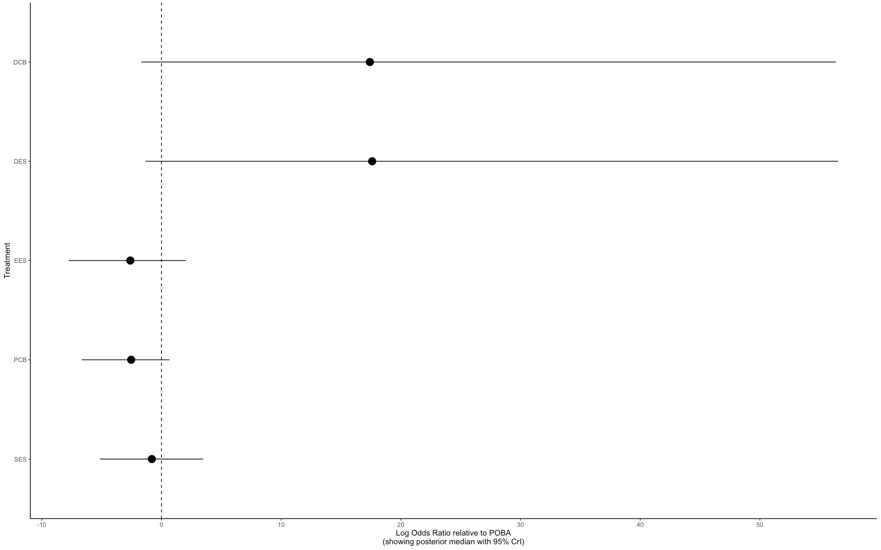
**

**(G) (H)**

**Figure 5.** NMA Forest plots display effect estimates of different interventions compared with POBA: (A) For TLR (RCTs only), (B) For MACE (RCTs only), (C) For TLF, (D) For TVR, (E) For MI, (F) For all-cause death, (G) For cardiac death, (H) For stent thrombosis


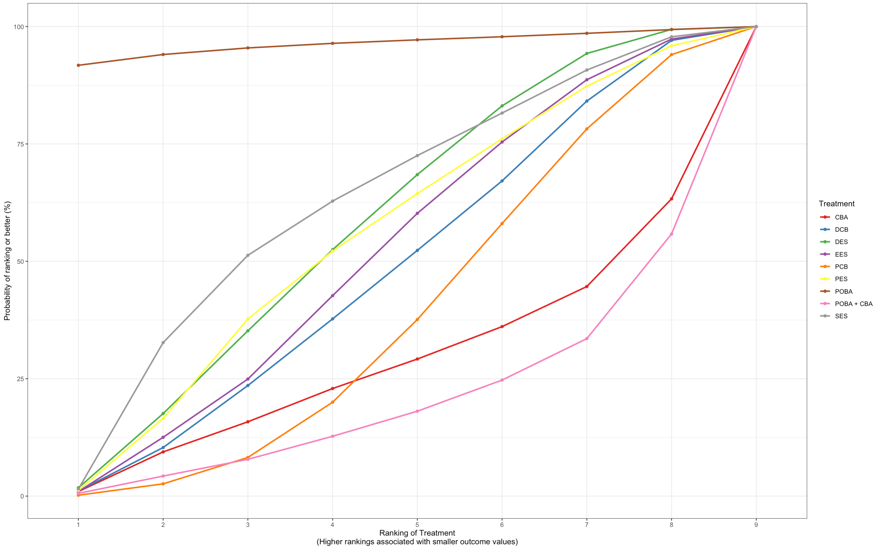

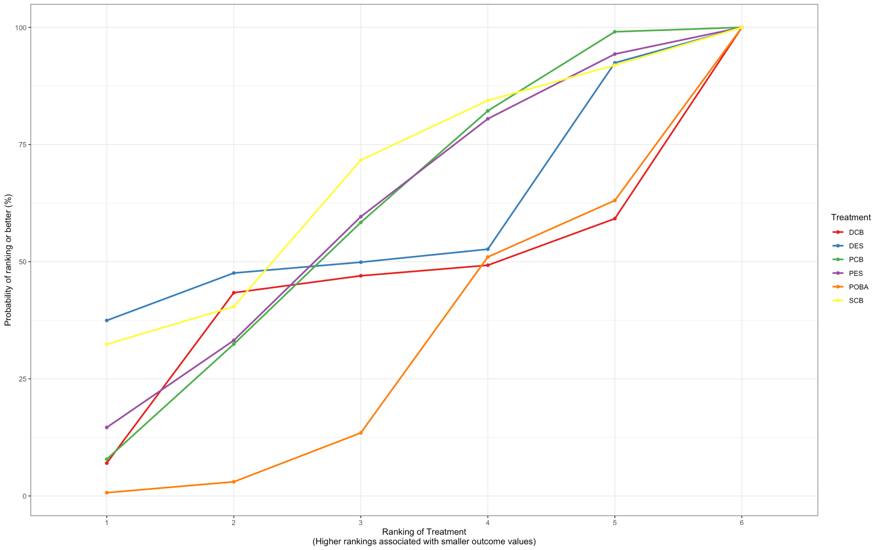

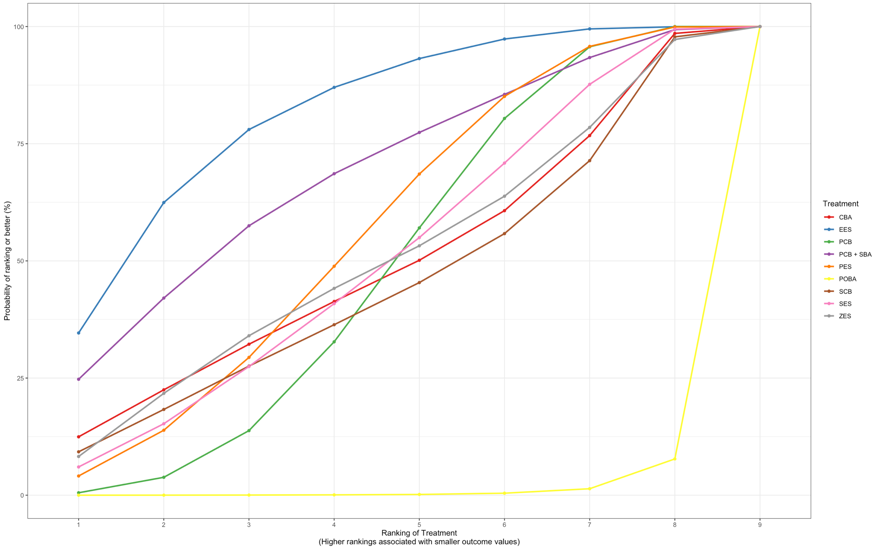

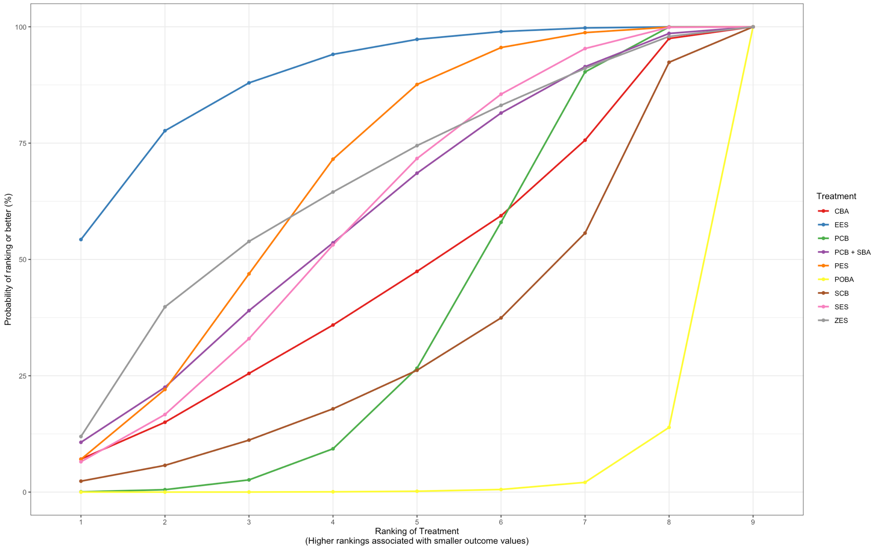
 (A) (B)

(C) (D)


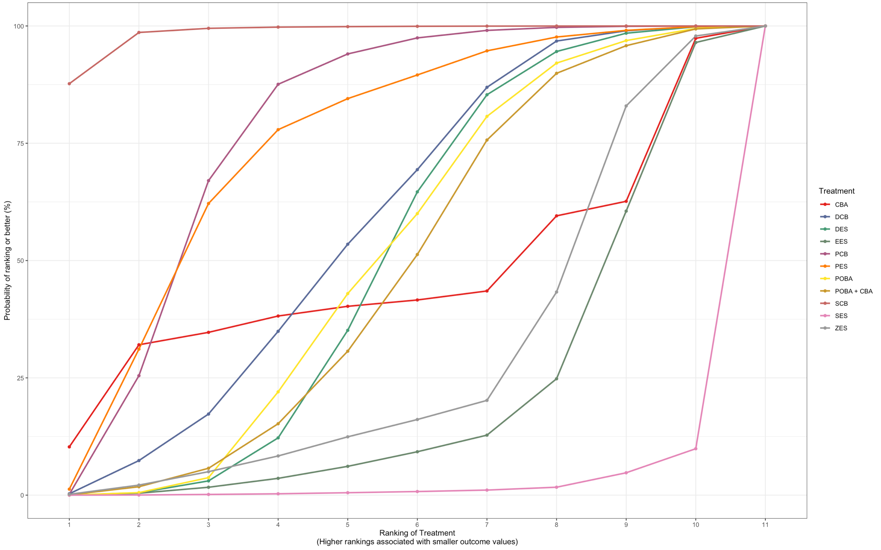

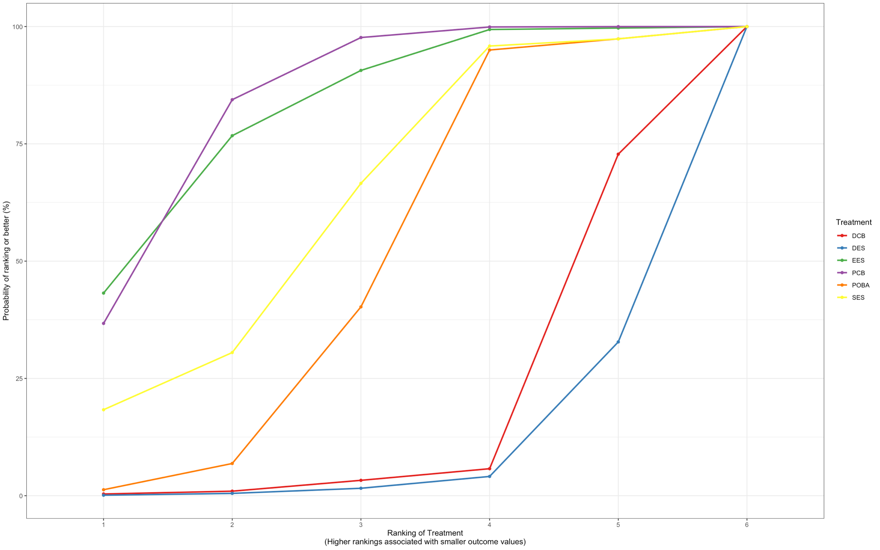

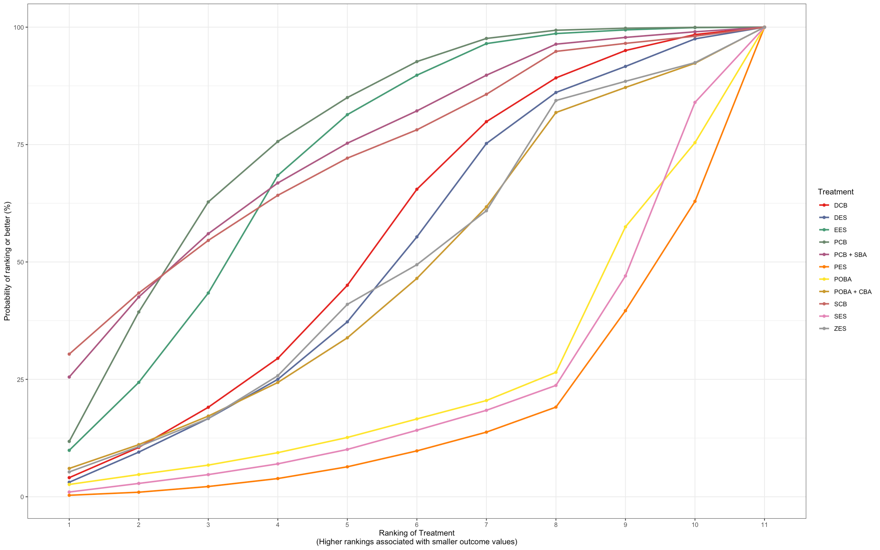

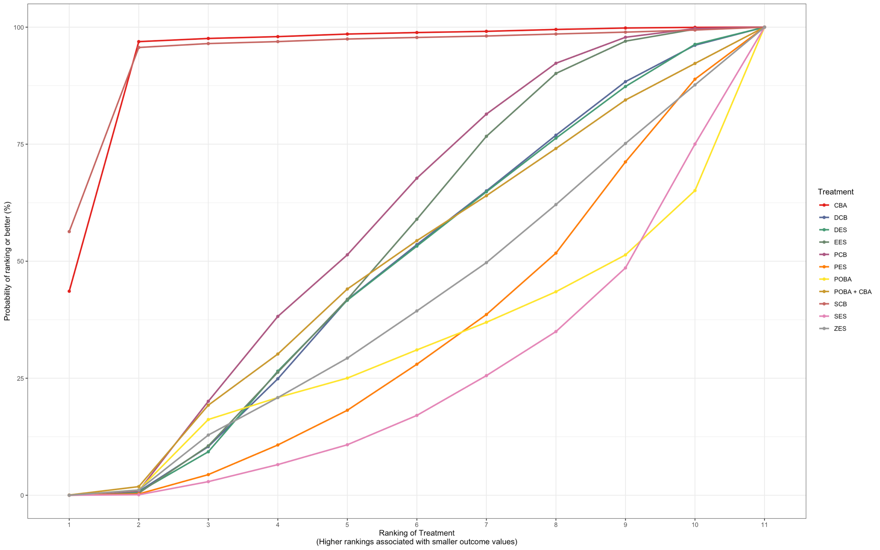
 (E) (F)

(G) (H)

**Figure 6**. Cumulative probability ranking chart and ranking probability histogram using the RE model. The SUCRA intuitively displays the sorting probability of each intervention group in the form of curves. (A) For TLR (RCT only), (B) For MACE (RCTs only), (C) For TLF, (D) For TVR, (E) For MI, (F) For all-cause death, (G) For cardiac death, (H) For stent thrombosis


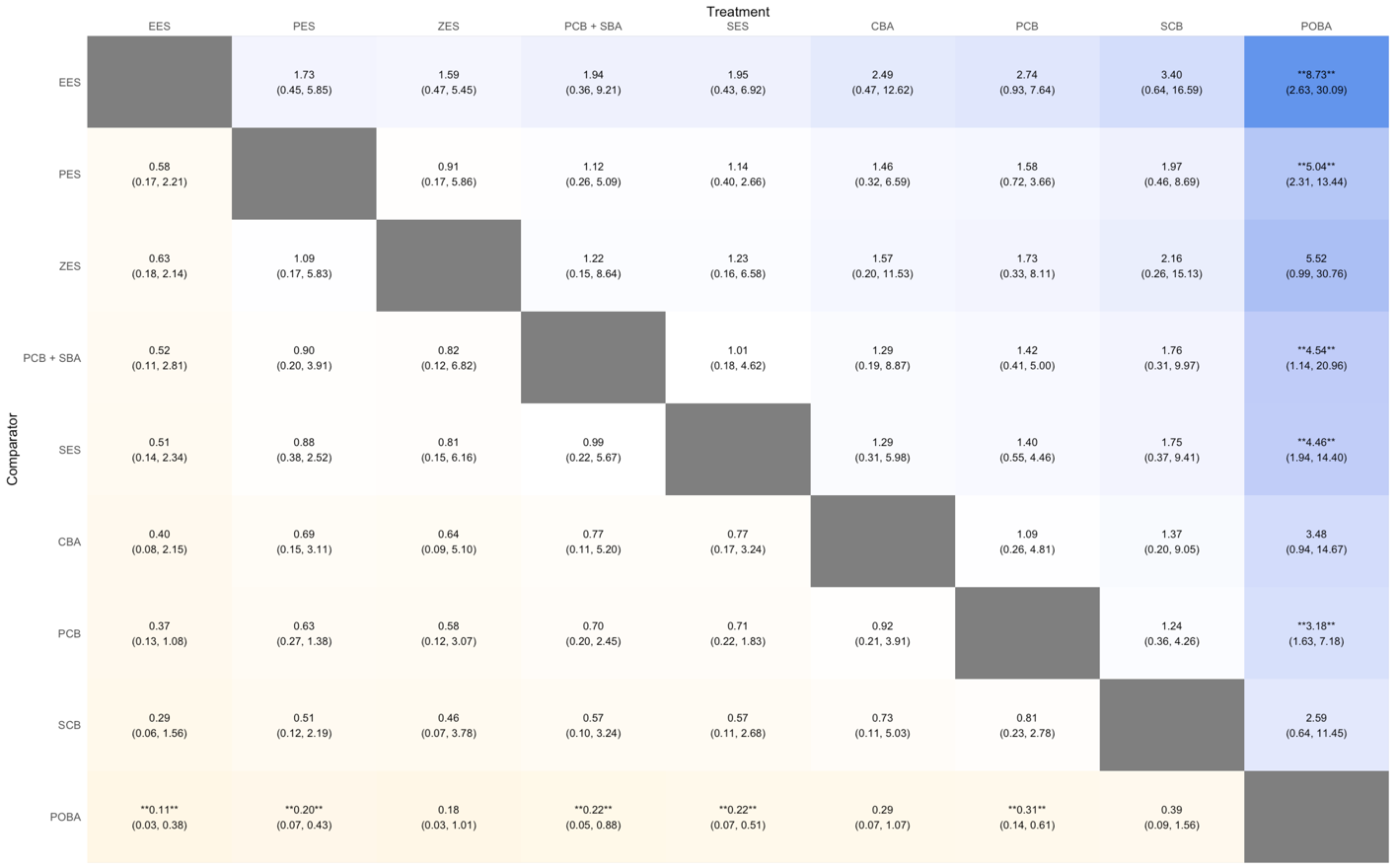
 (A)


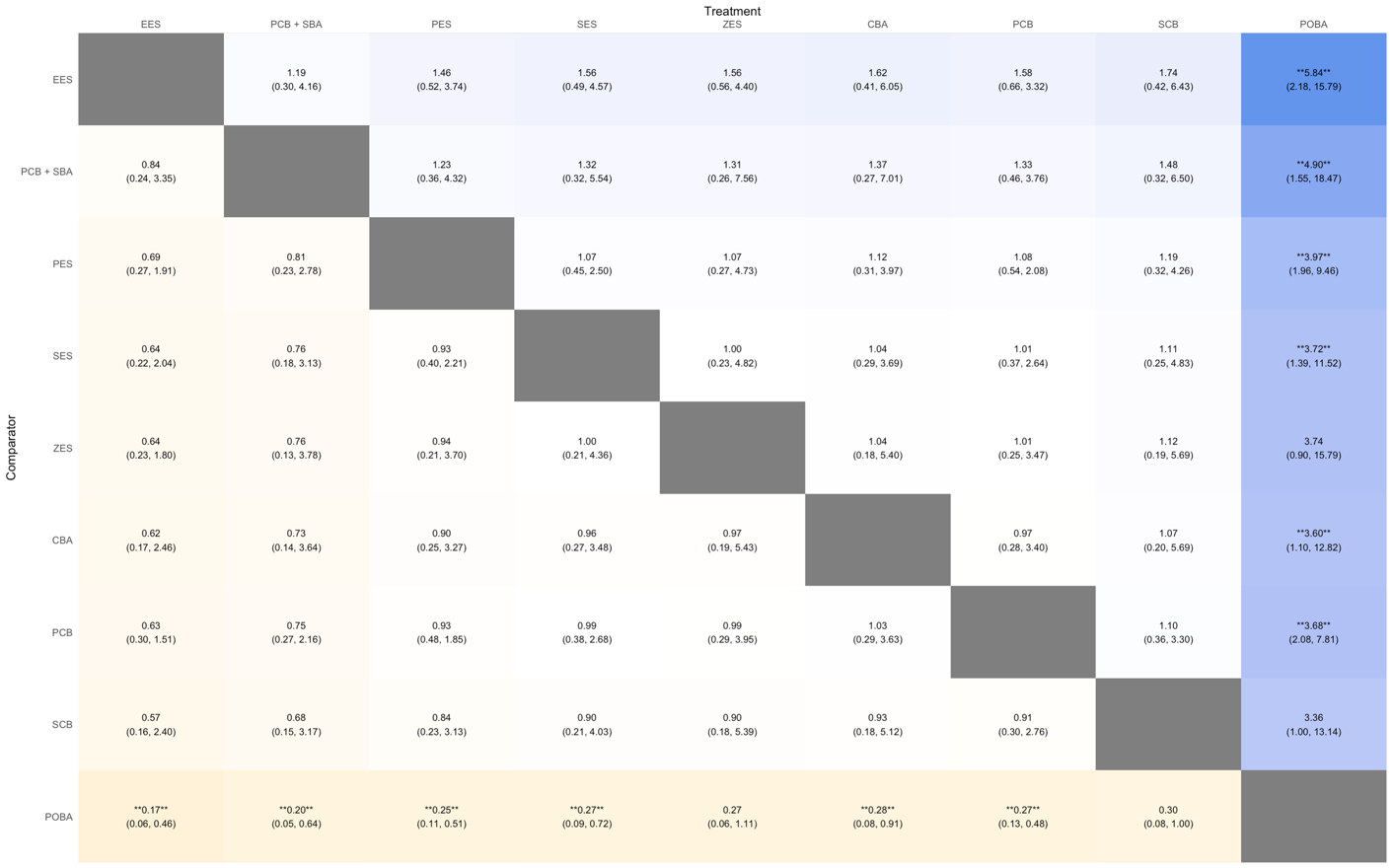
 (B)


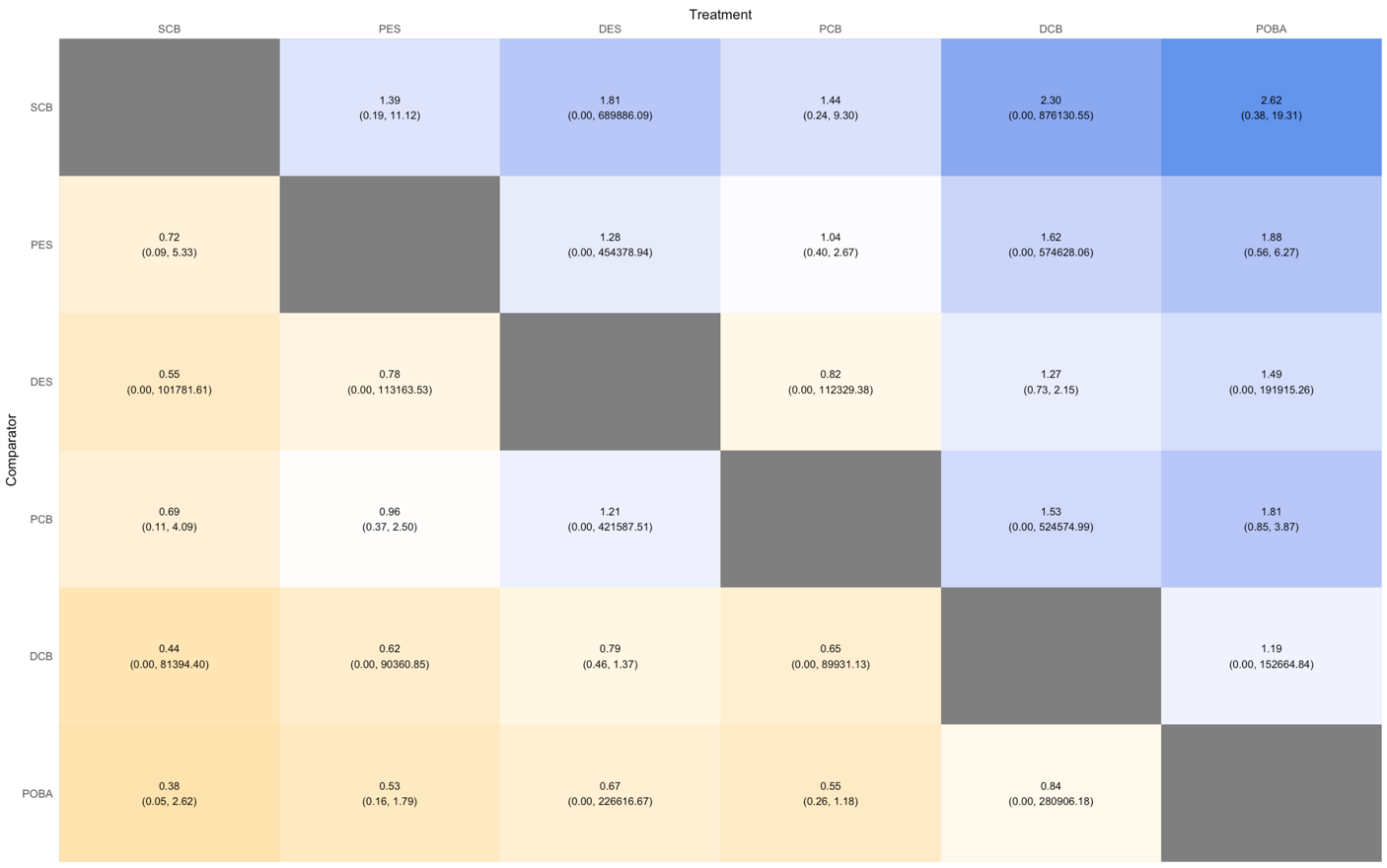


(C)


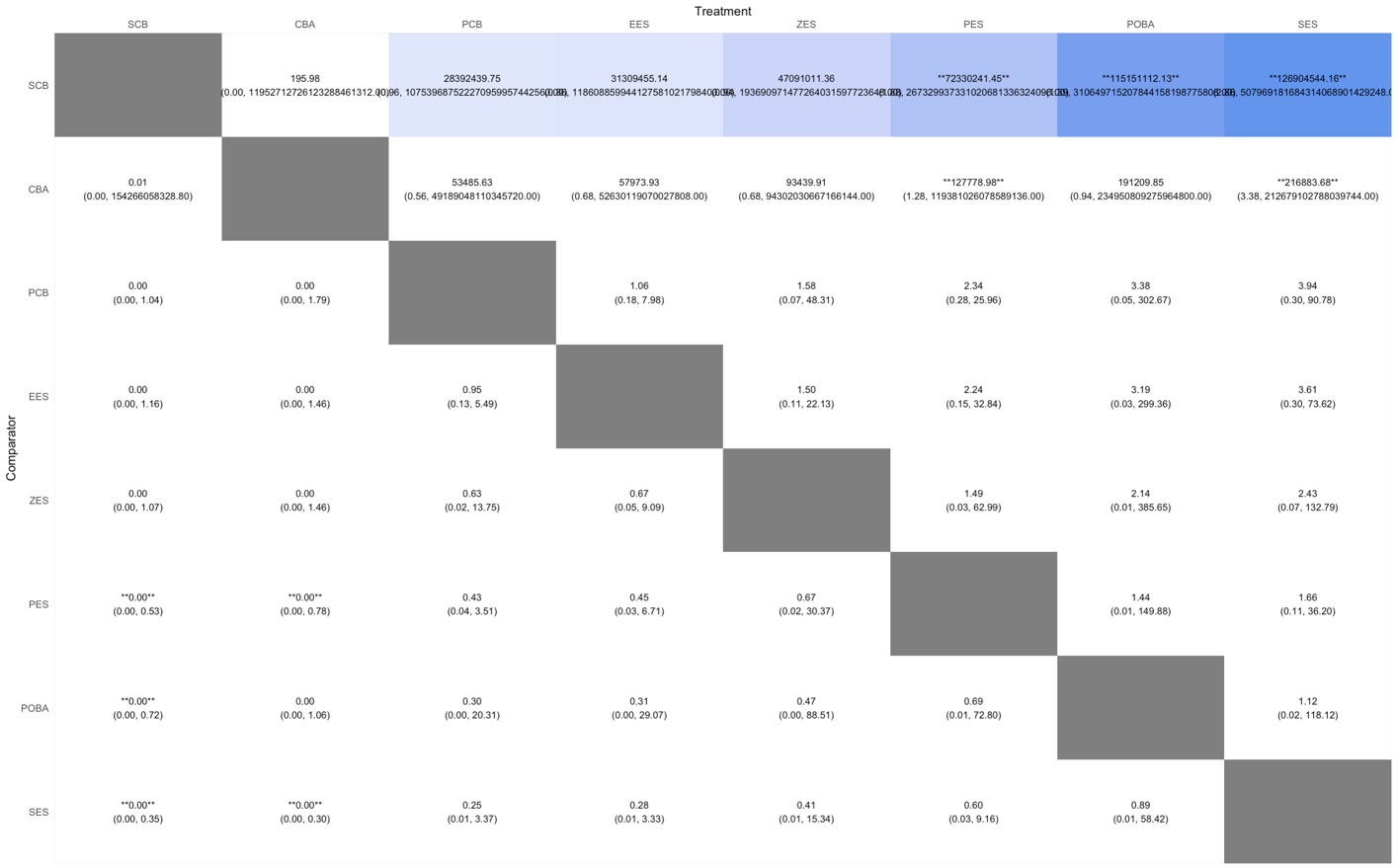

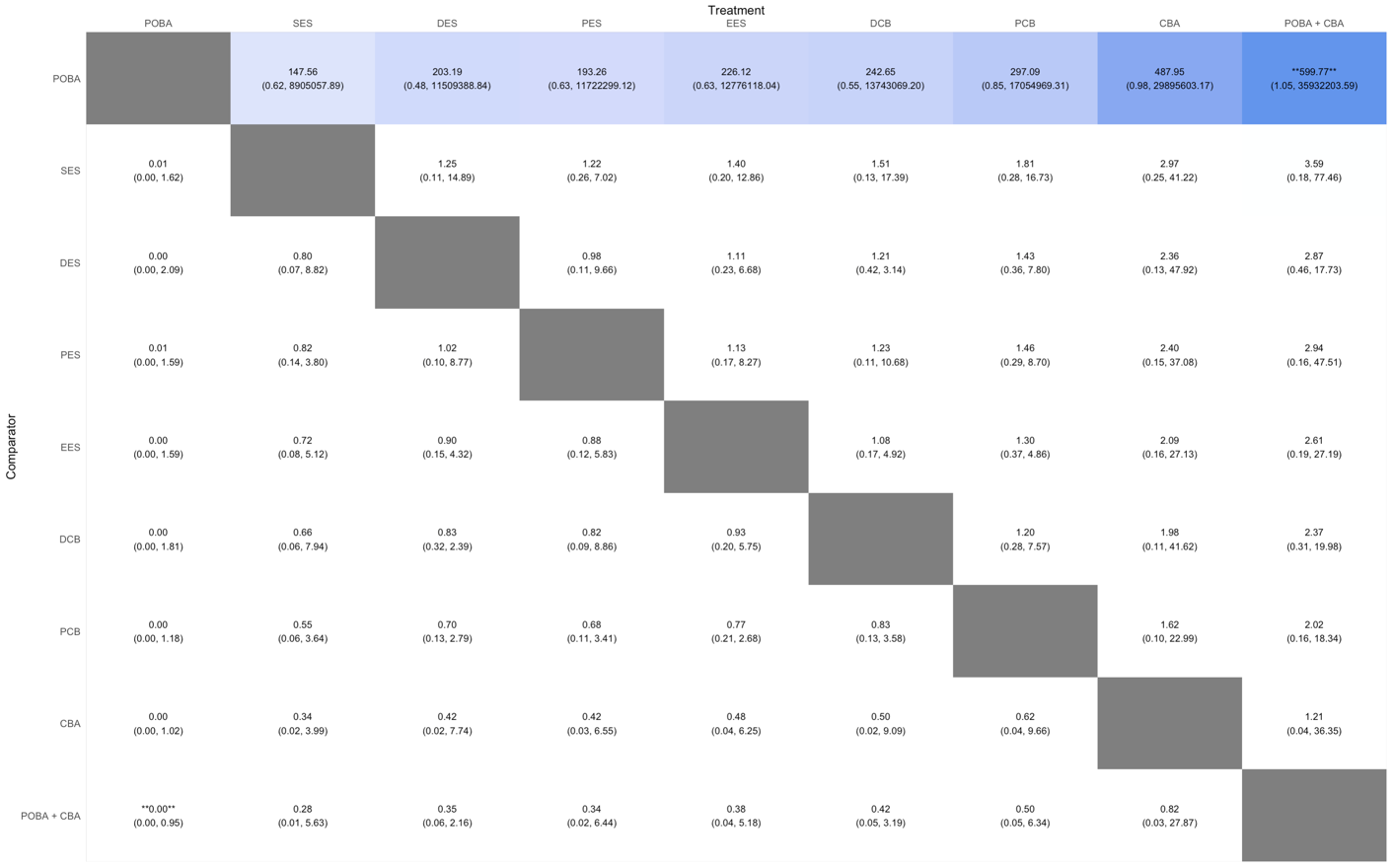
 (D)

(E)


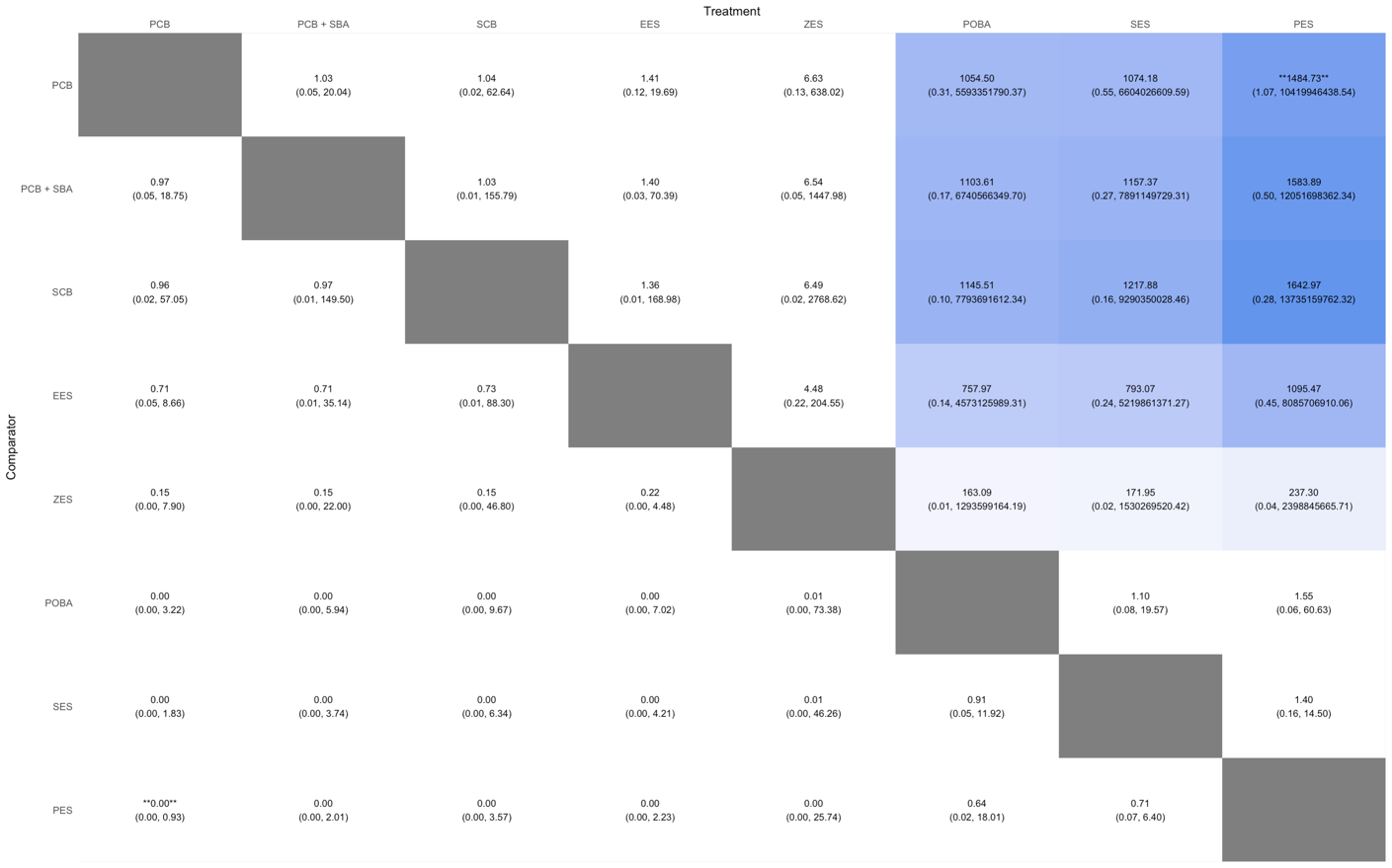

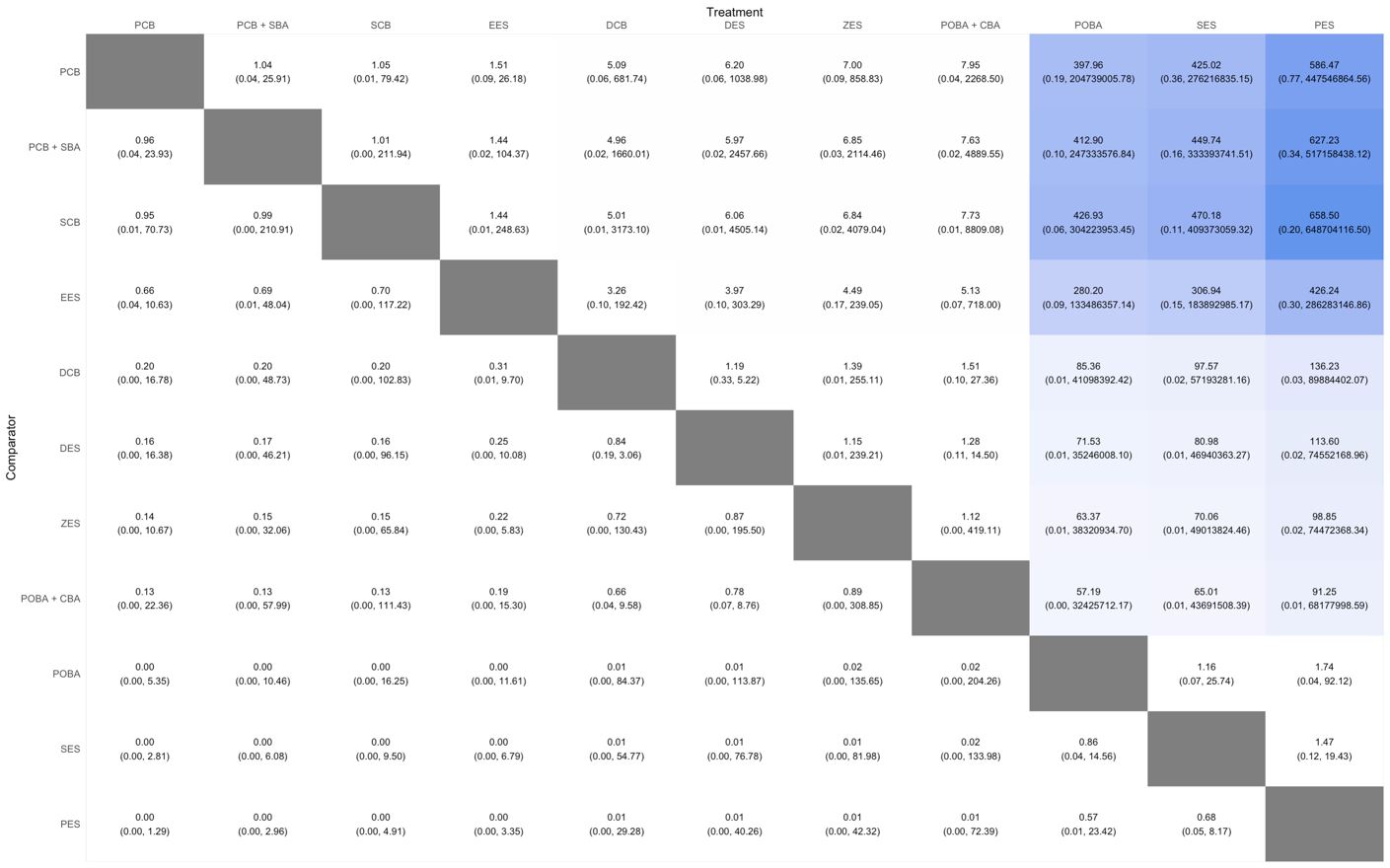
 (F)

(G)


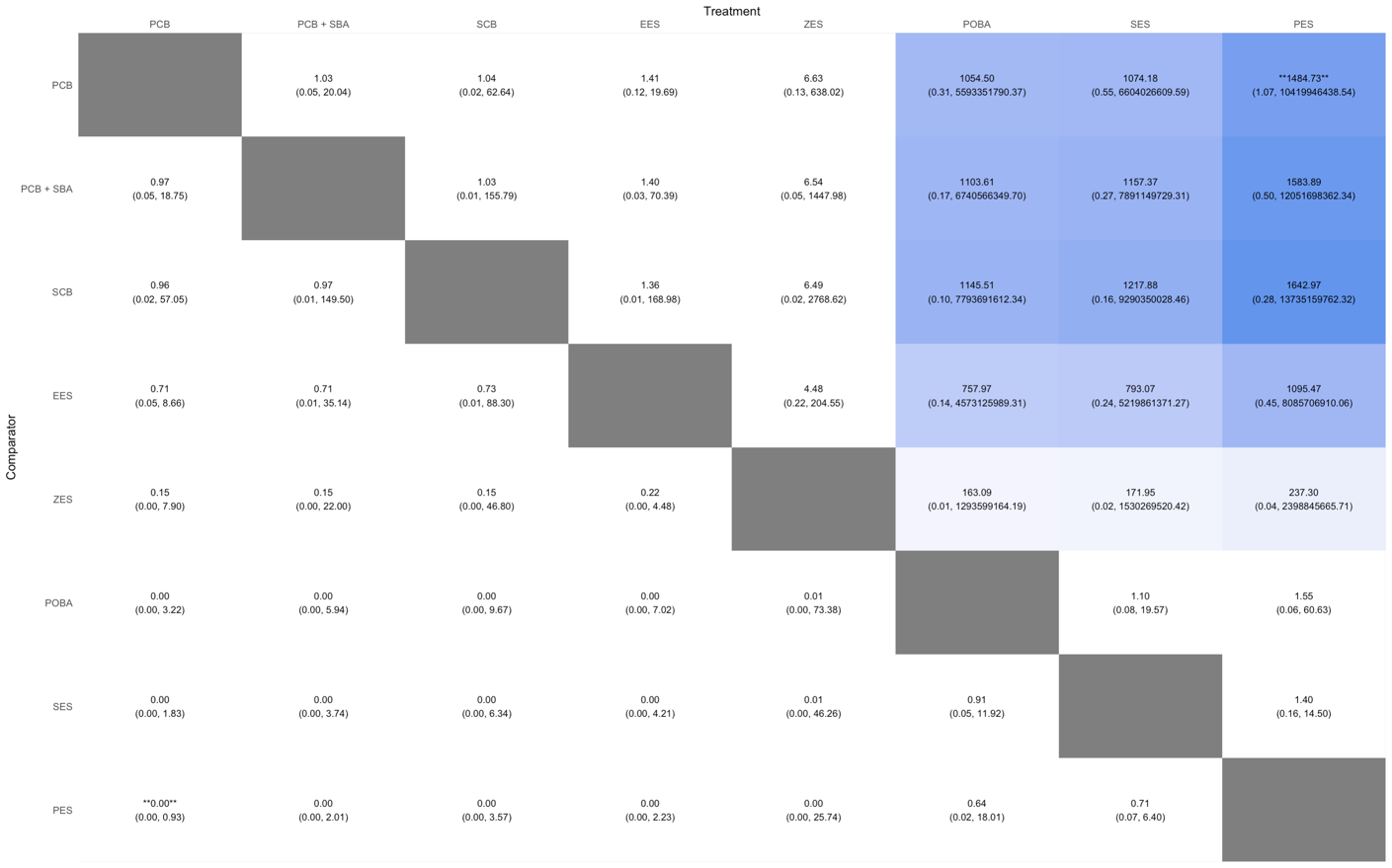
 (H)

**Figure 7.** Ranking chart heat map. (A) For TLR (RCTs only); (B) For MACE (RCTs only); (C) For TLF; (D) For TVR; (E) For MI; (F) For all cause death; (G) For cardiac death; (H) For stent thrombosis. Data are ORs (95% CrI) of the treatment on the top, compared with the comparator on the left. OR > 1.0 shows an advantage for the treatment, whereas OR < 1.0 shows an advantage for the comparator. Statistically significant results (P < 0.05) are marked by the symbols (**). OR = odds ratio; CrI = credible interval.


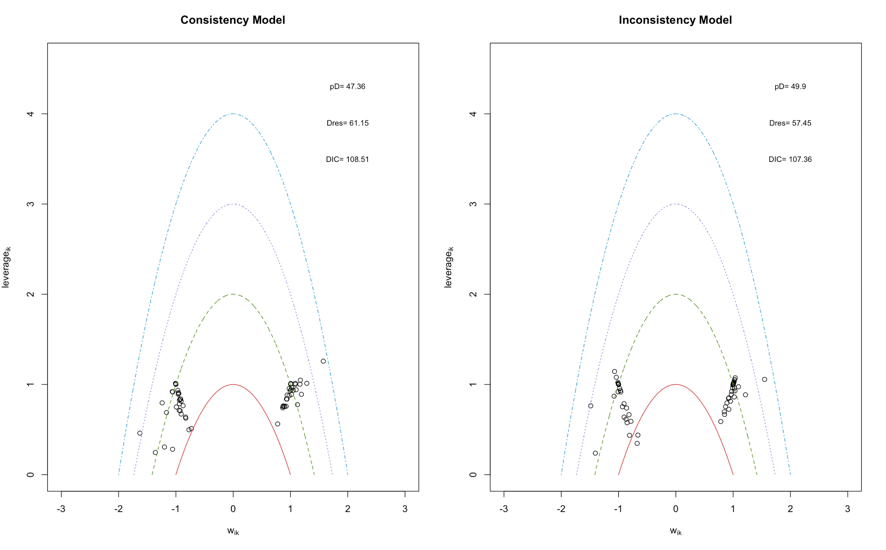

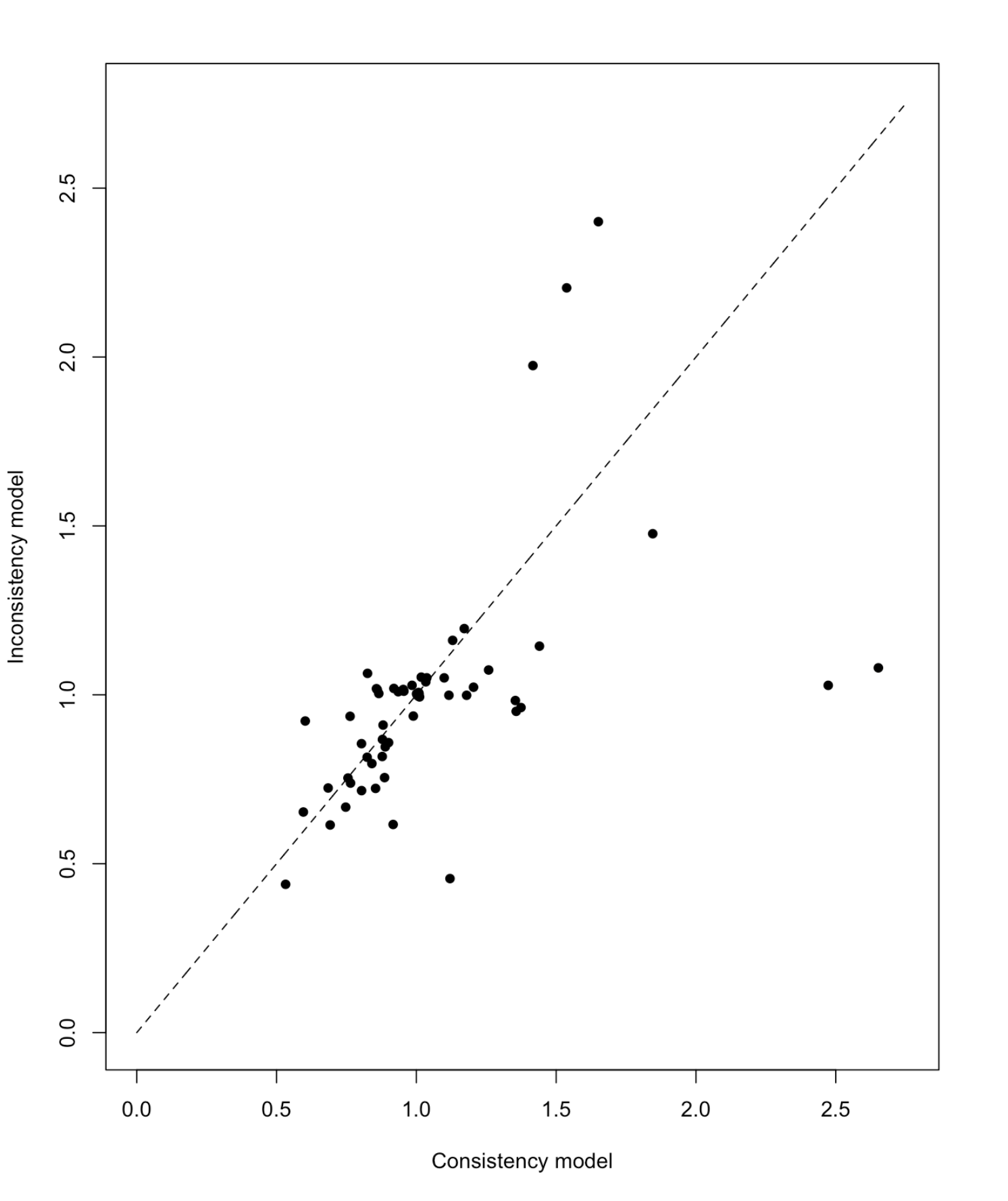


(A)


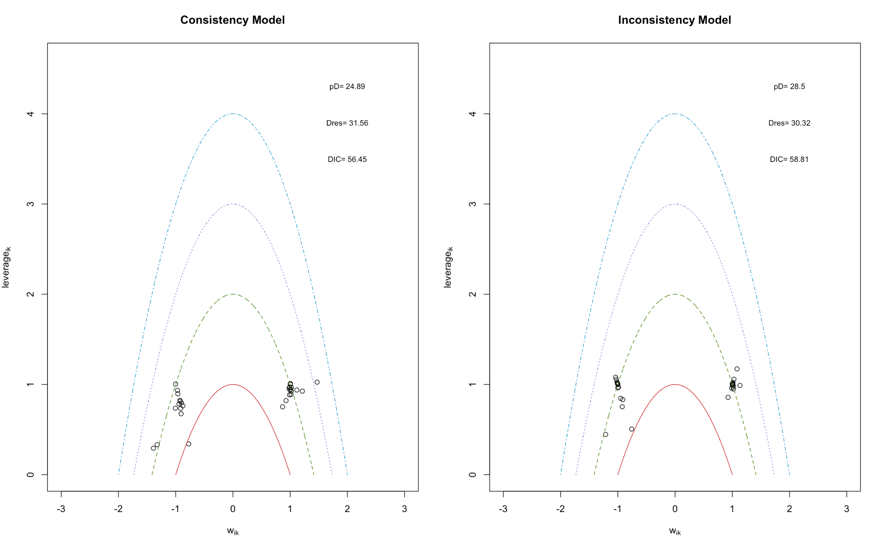

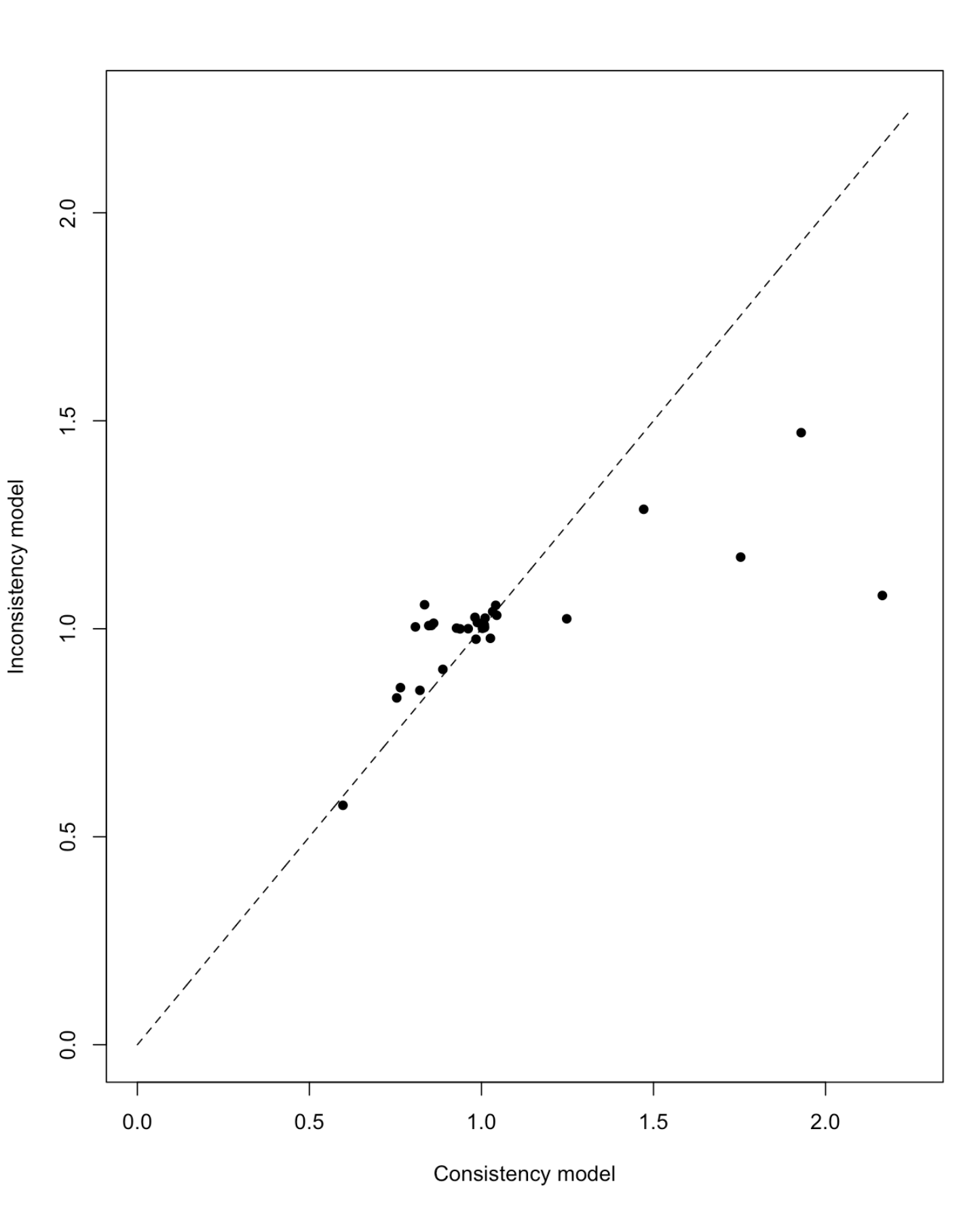


(B)


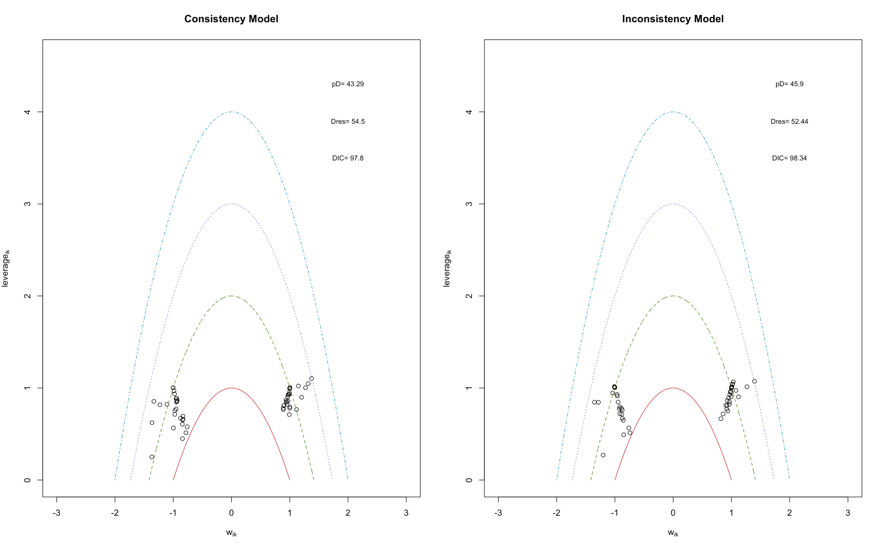

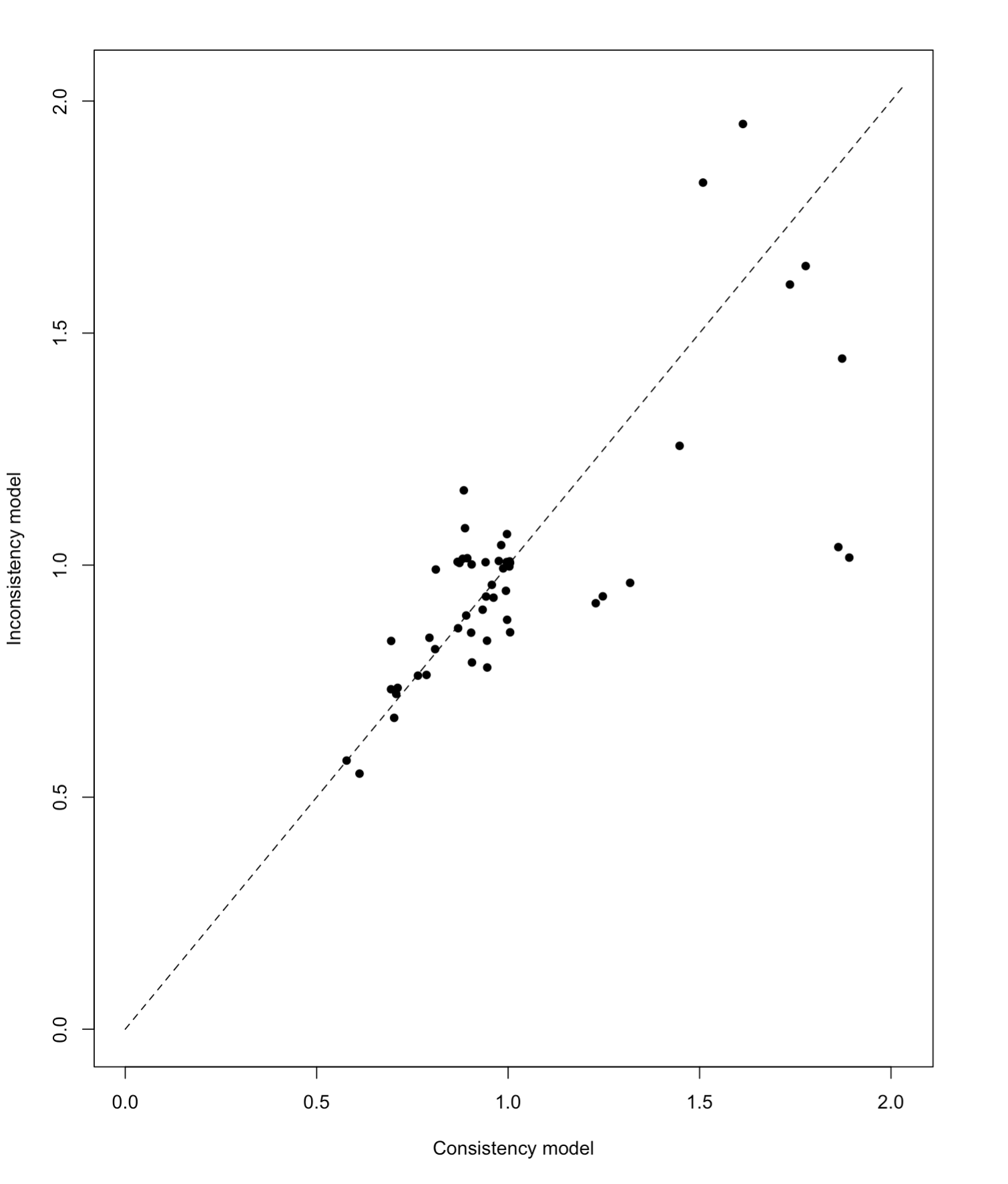


(C)


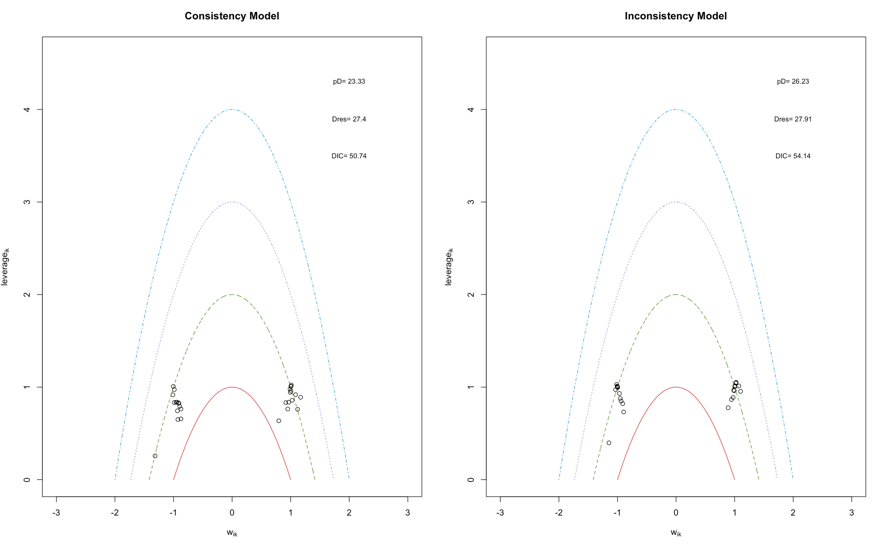

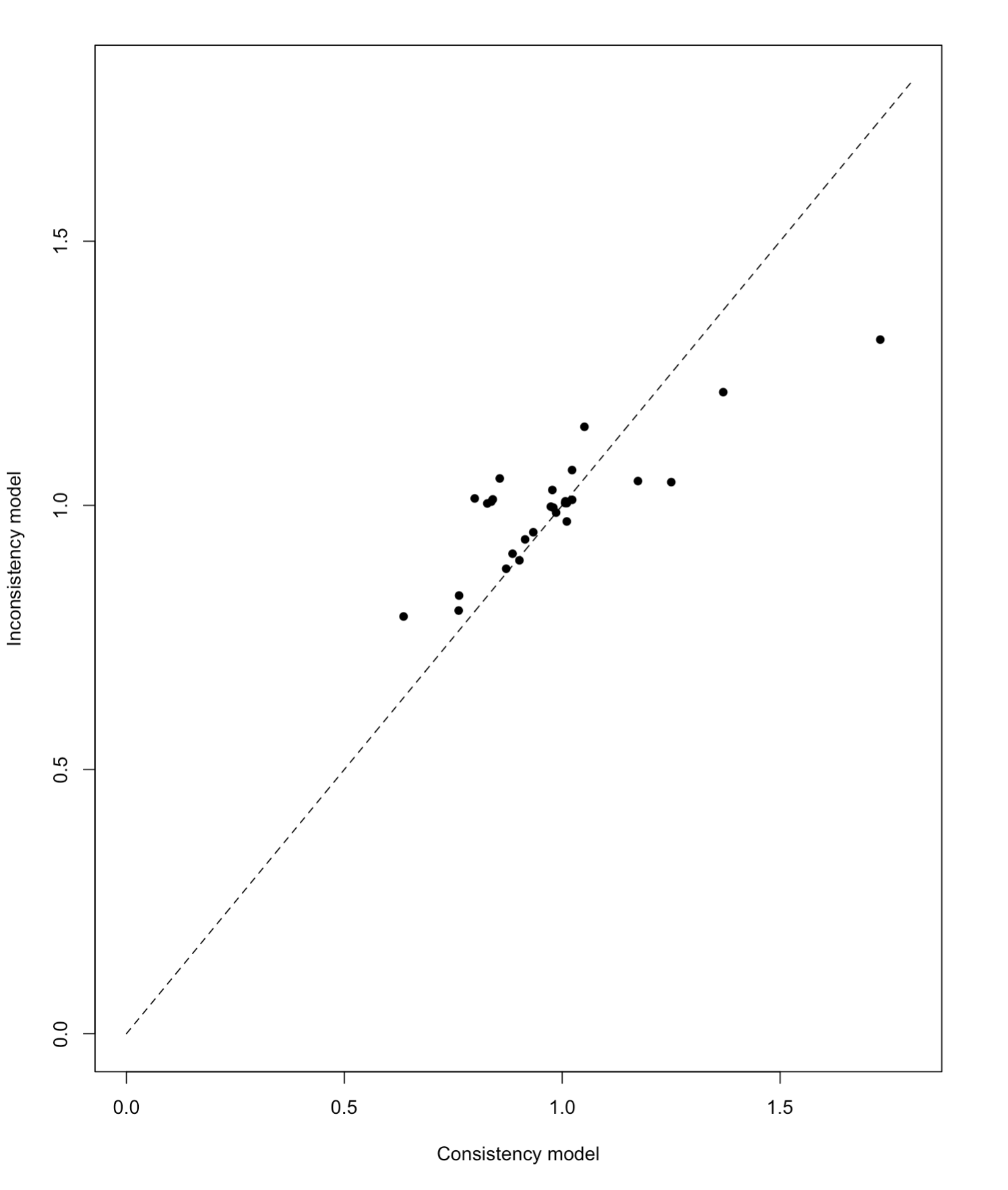


**(D)**


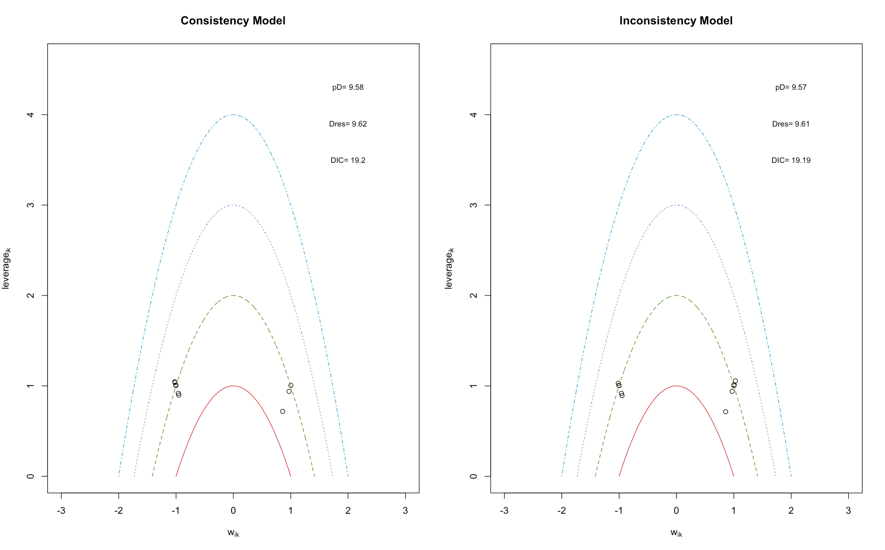

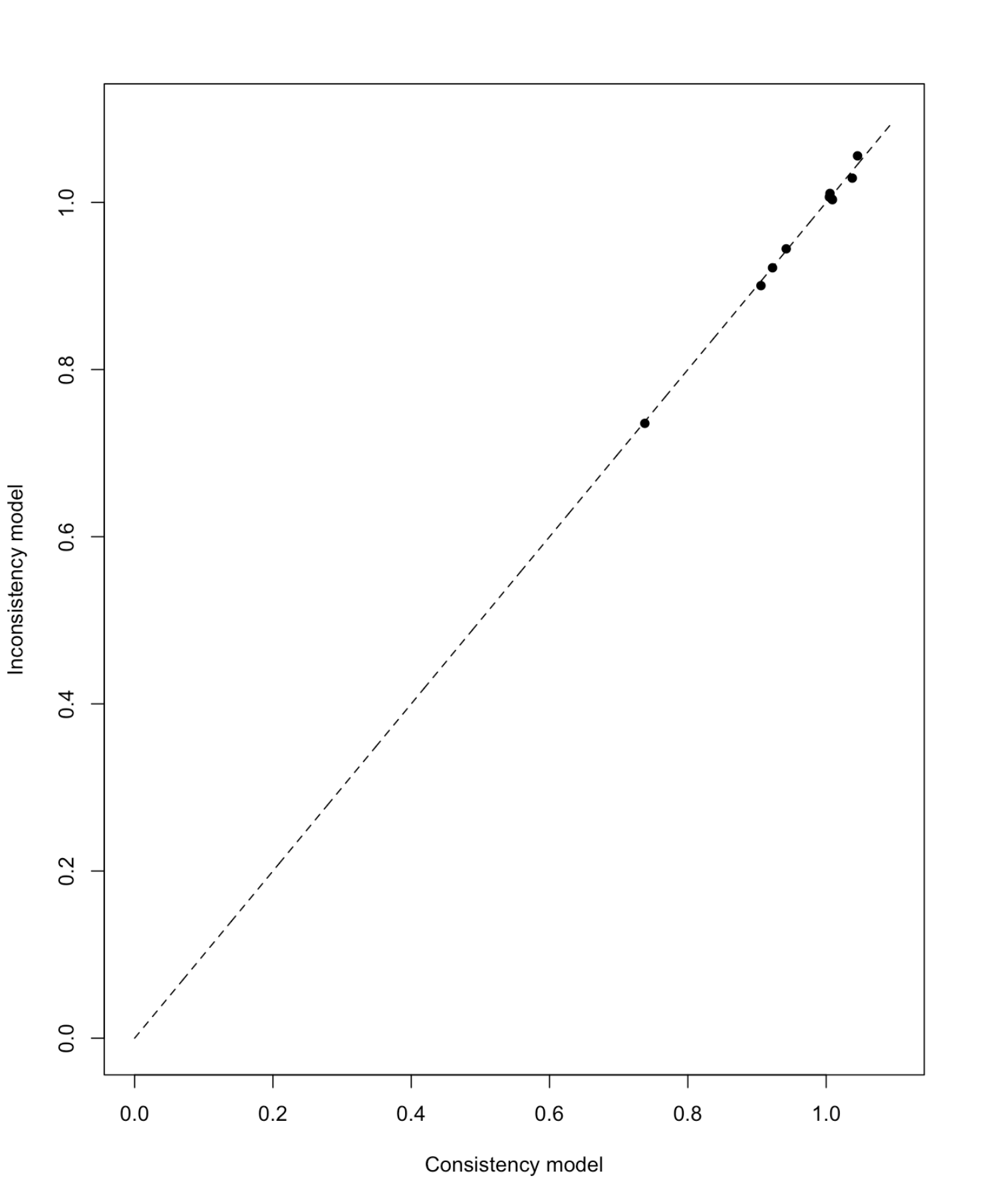


**(E)**


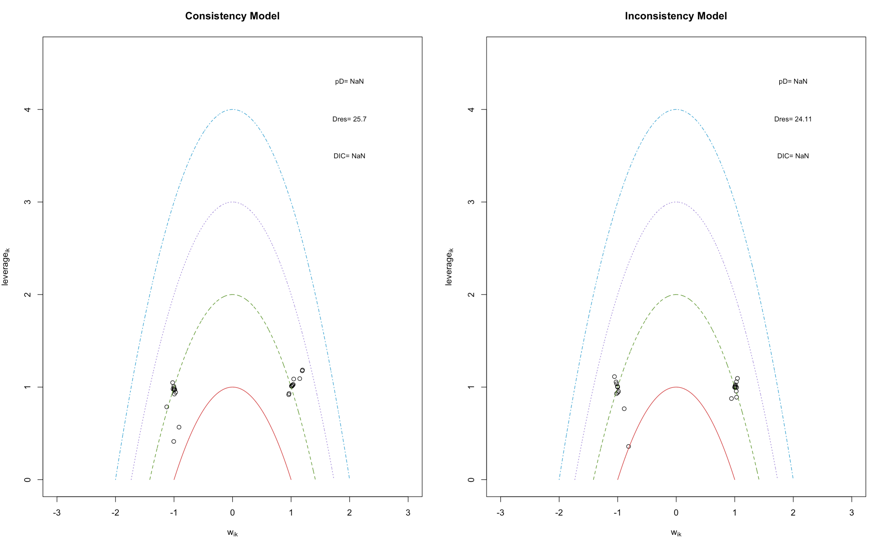

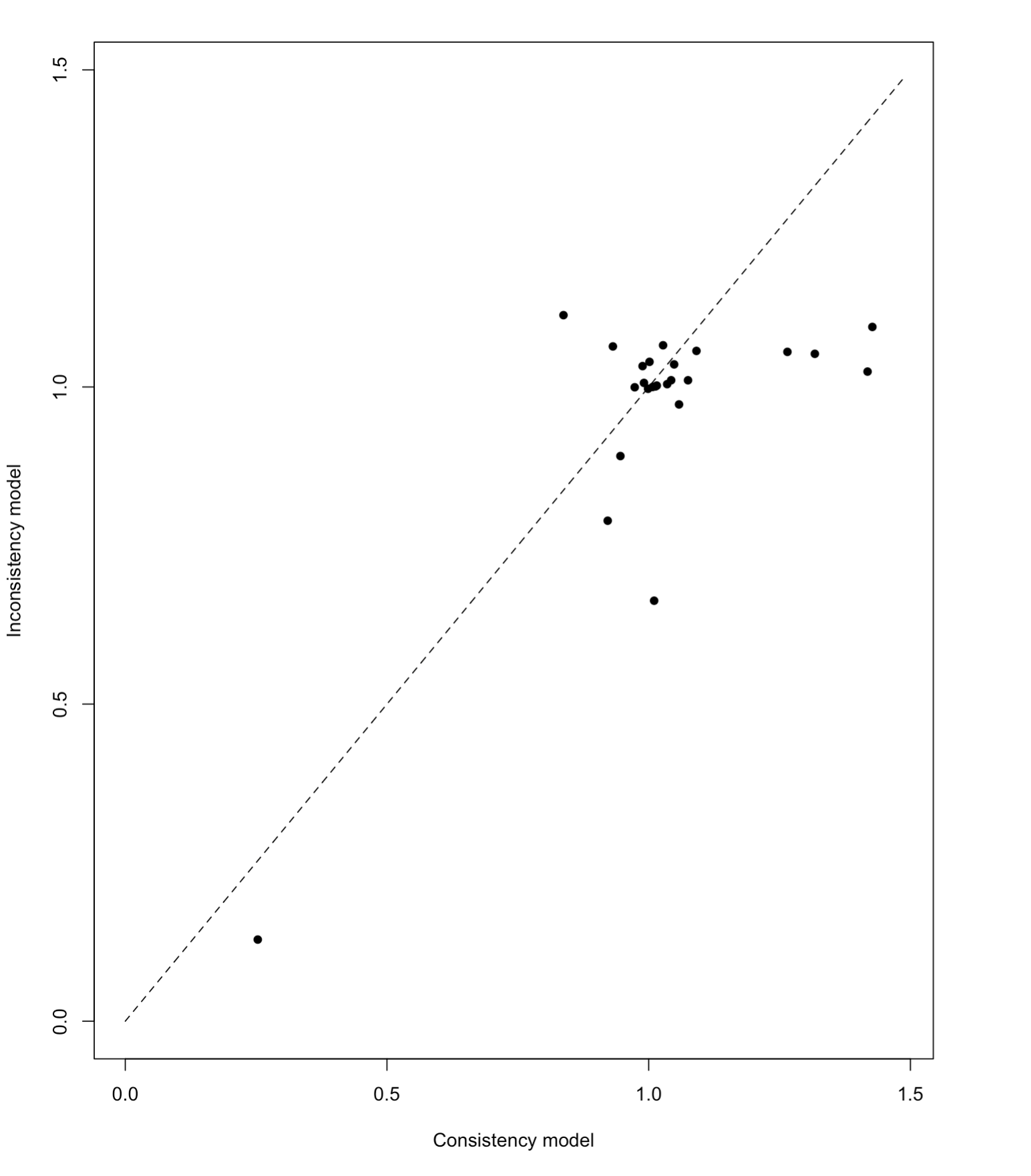


**(F)**

**(G)**


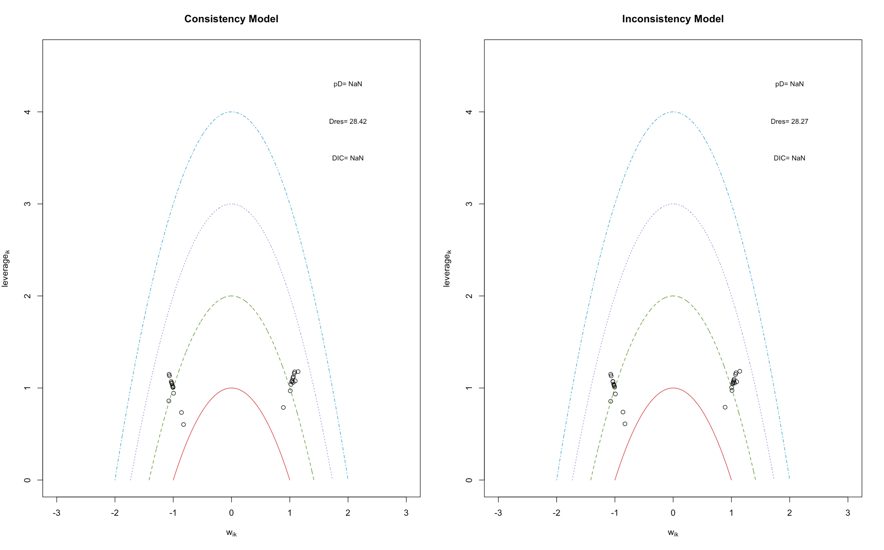

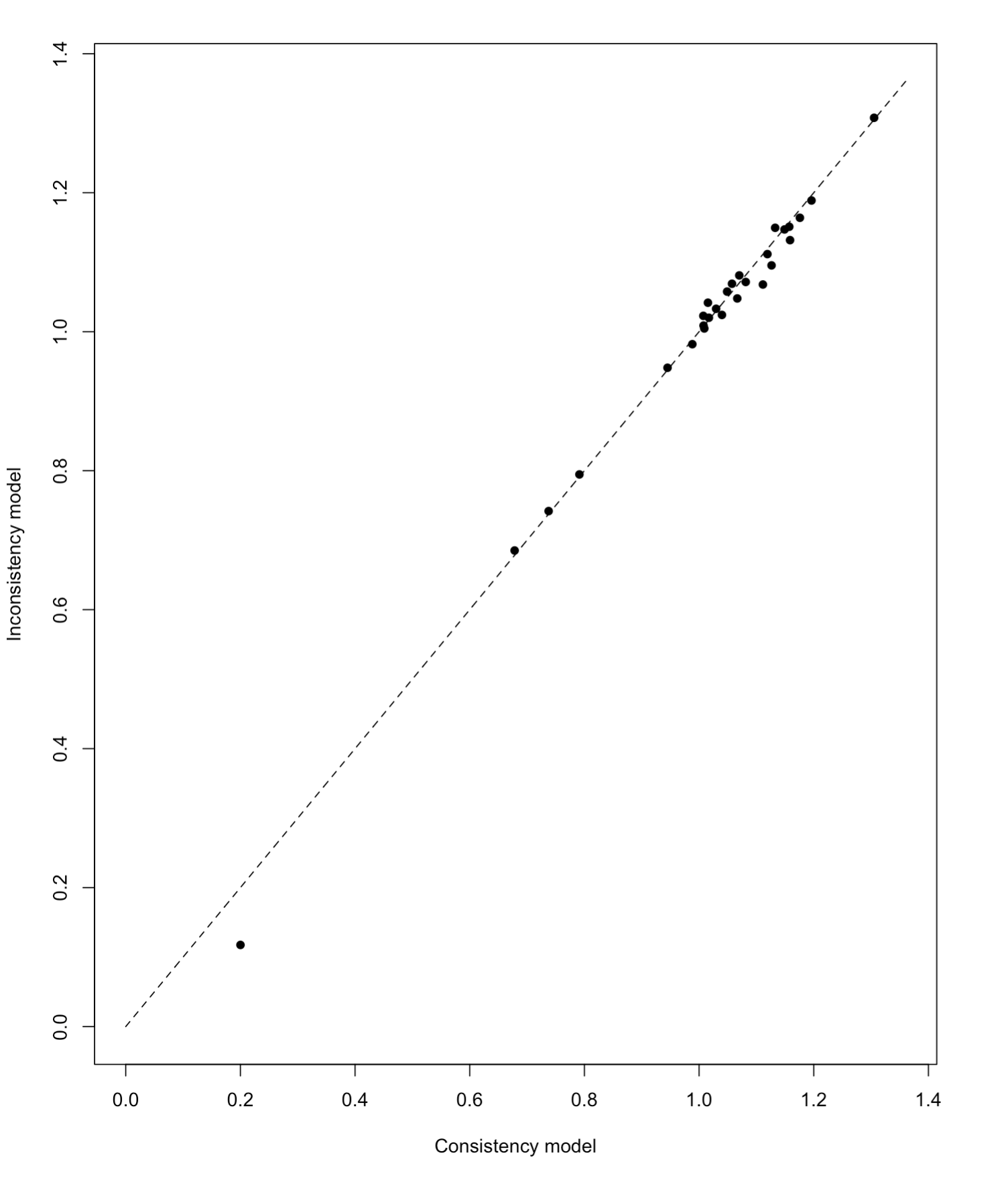

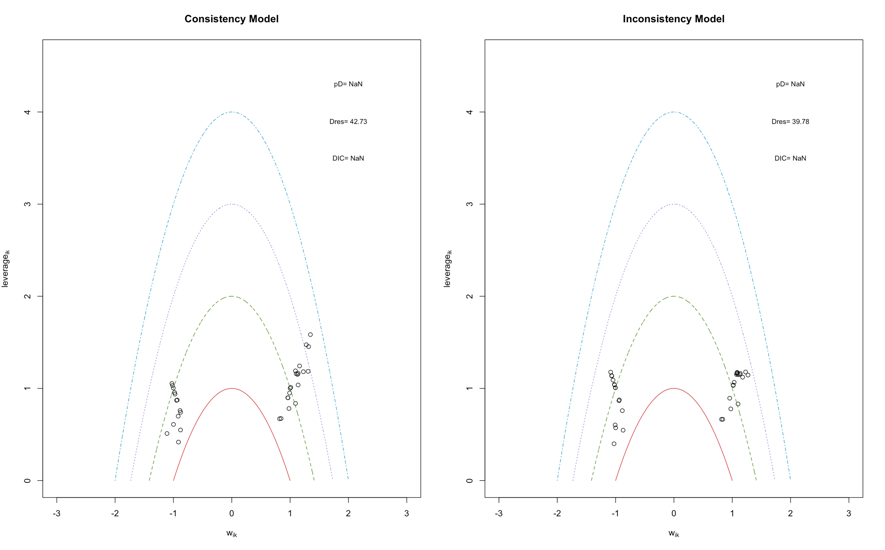

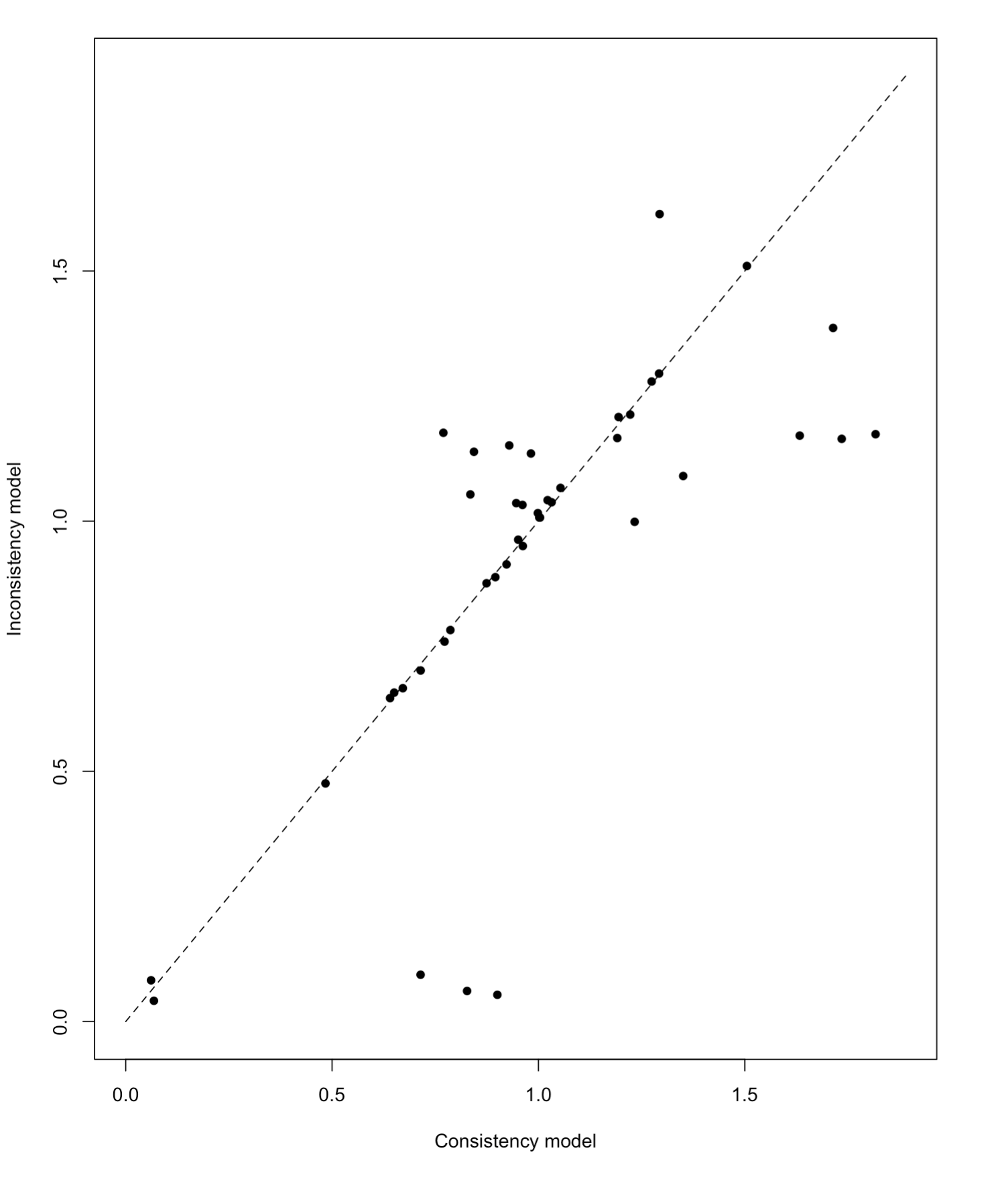


**(H)**


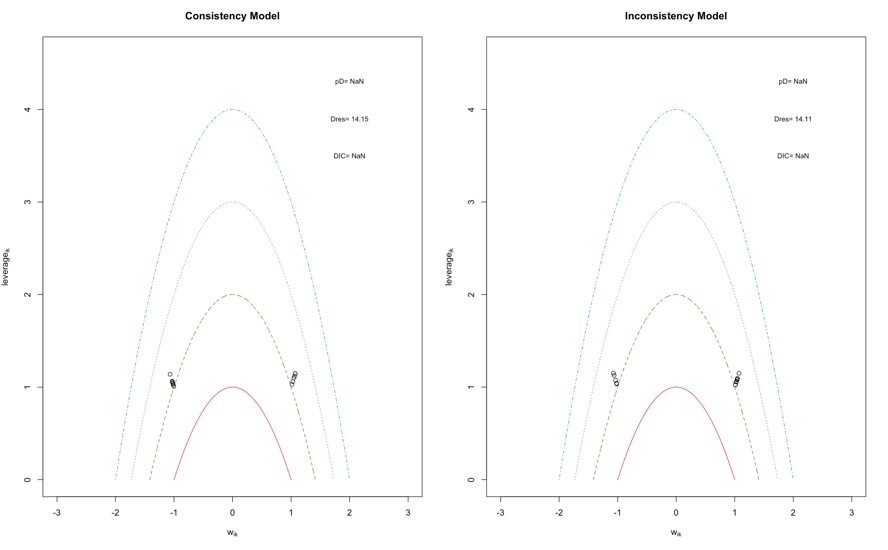

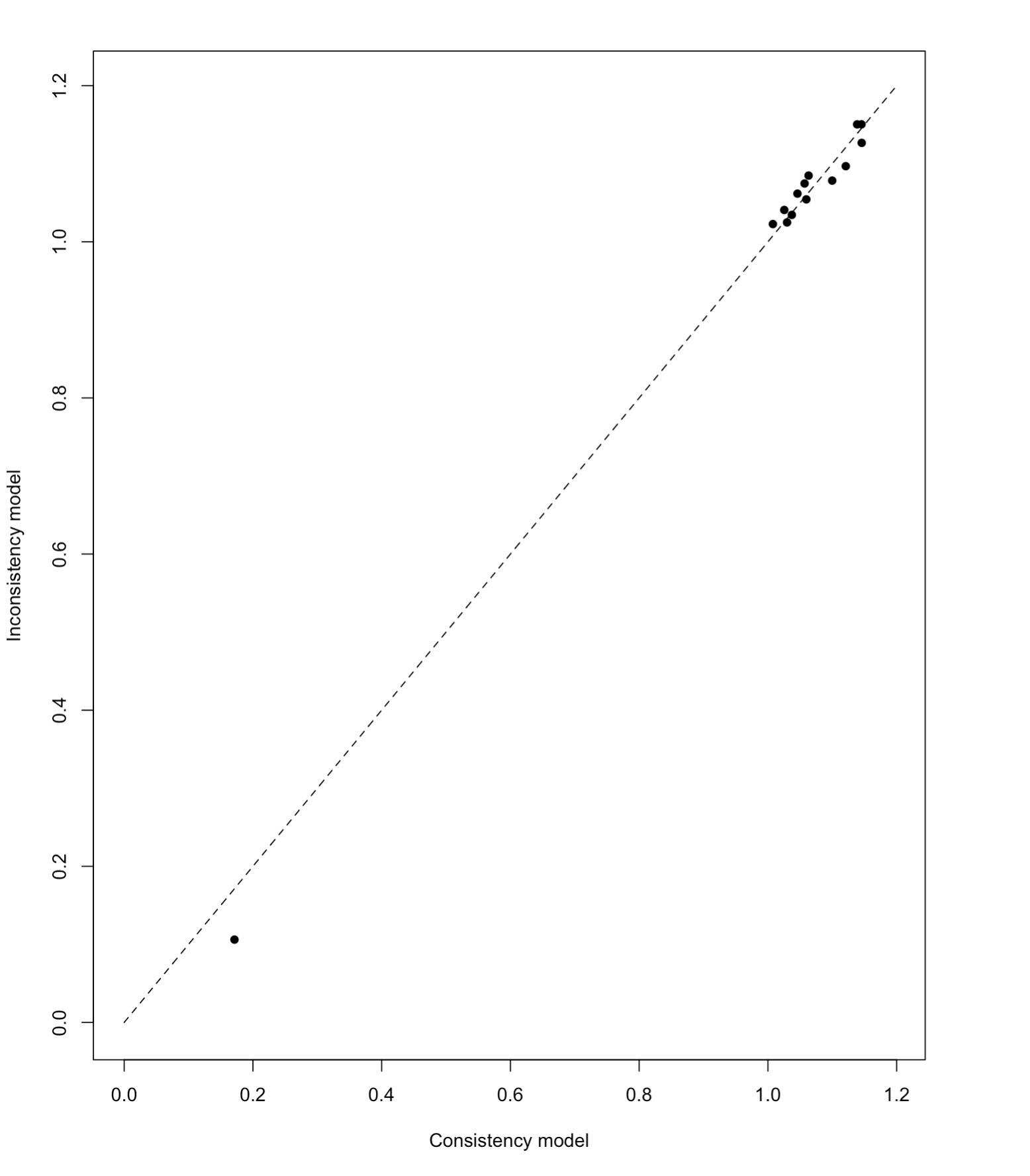


**(I)**

**(J)**


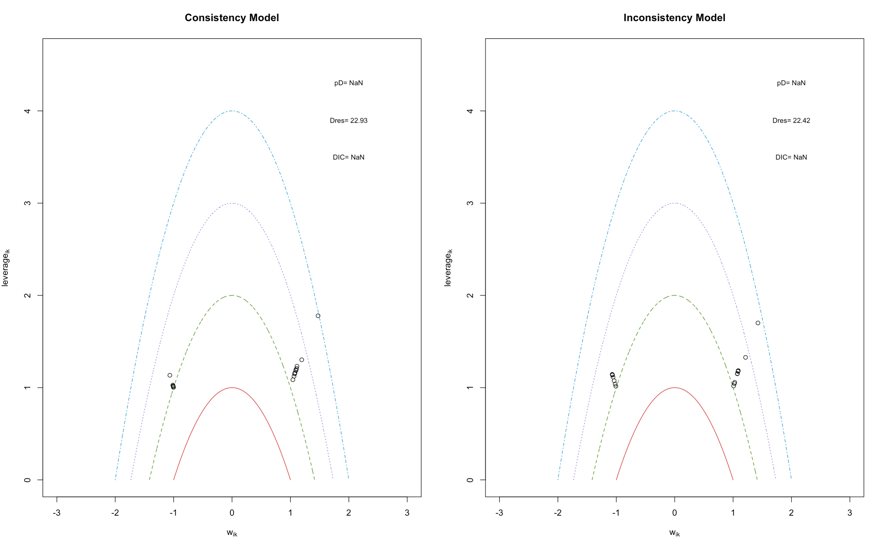

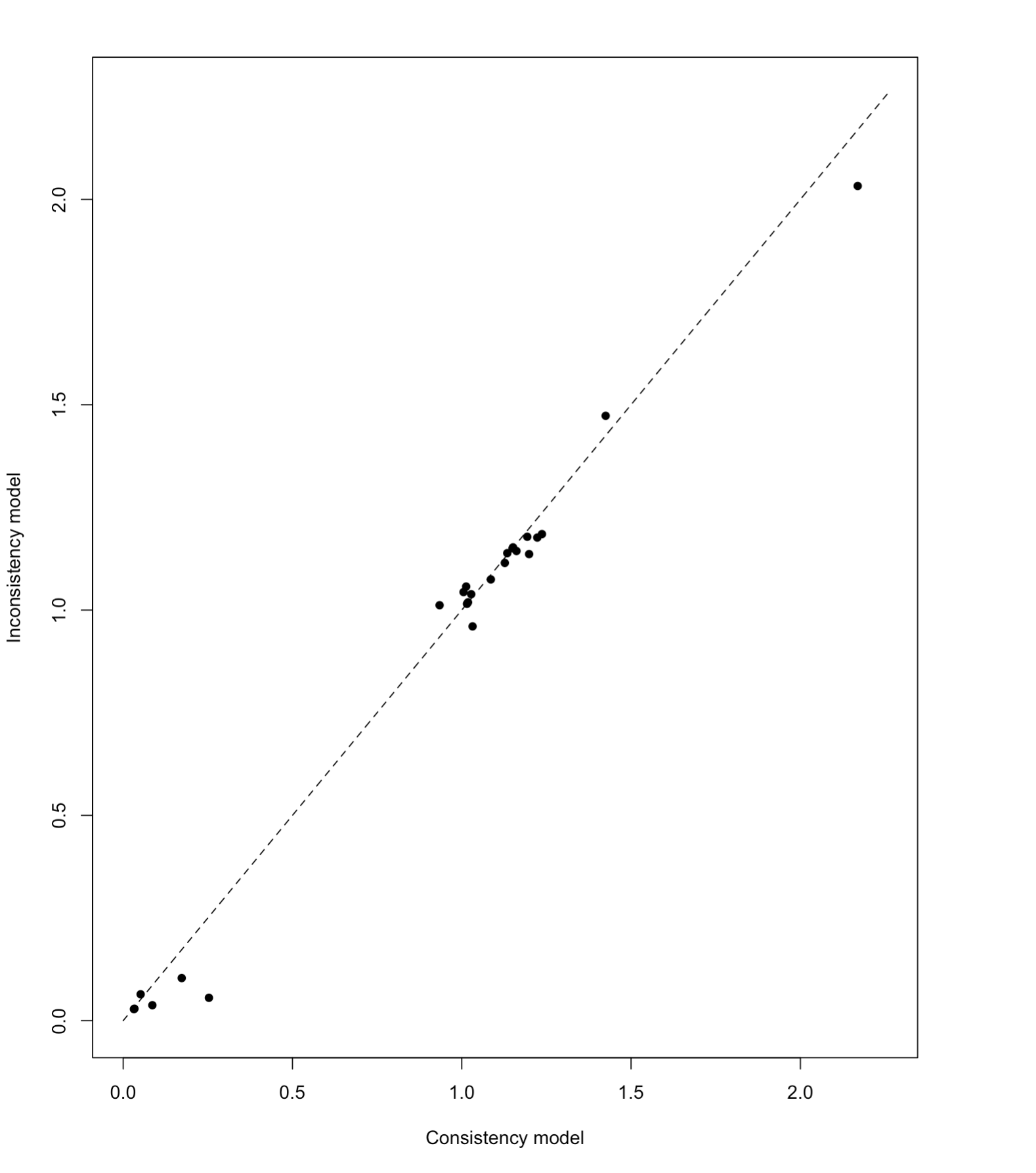


**Figure 8.** Consistency vs. Inconsistency Models and Individual data points’ posterior mean deviance contributions for the Random Effect consistency model vs the Random Effect inconsistency model: (A) For TLR; (B) For TLR (RCTs only); (C) For MACE; (D) For MACE (RCTs only); (E) For TLF; (F) For TVR; (G) For MI; (H) For all-cause death; (I) For Cardiac death; (J) For stent thrombosis

(A) DES vs DCB


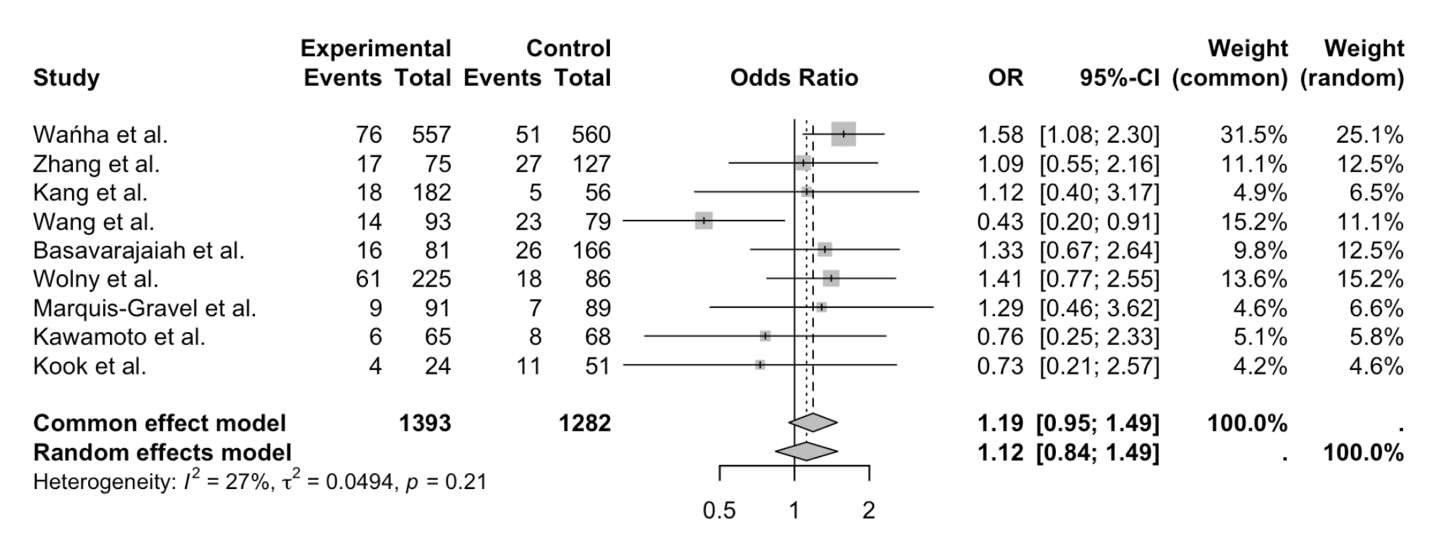


(B) DES vs POBA


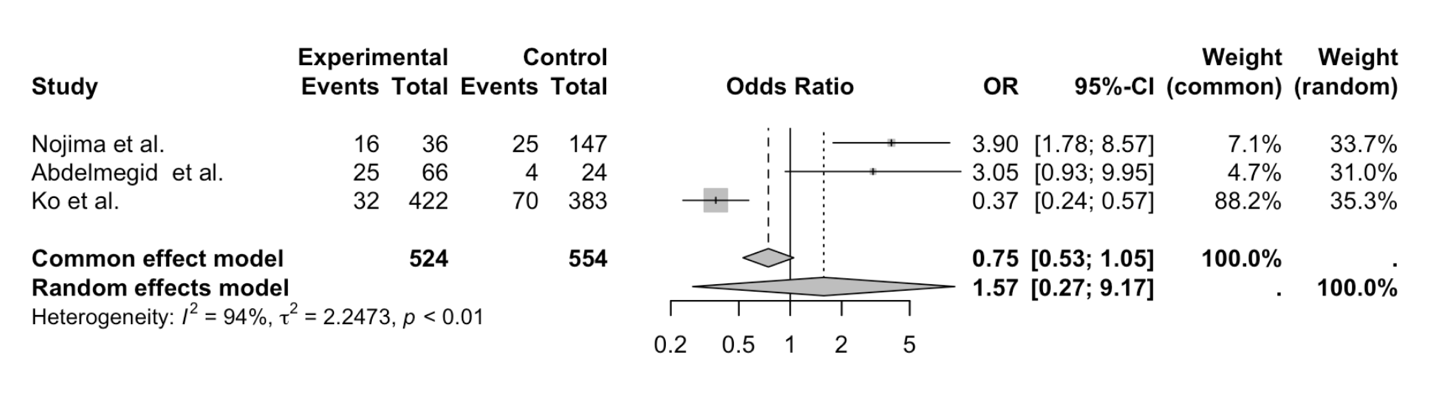

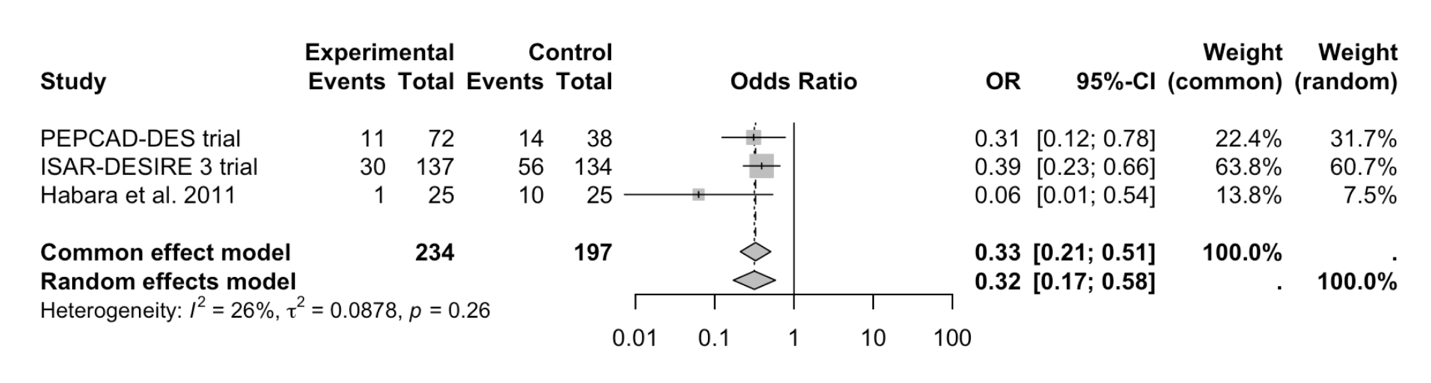


(C) PCB vs POBA

(D) EES vs PCB


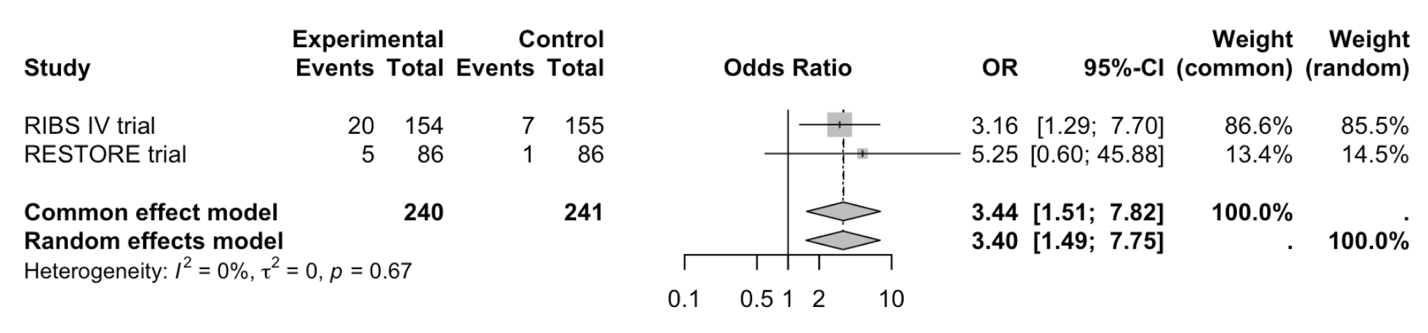

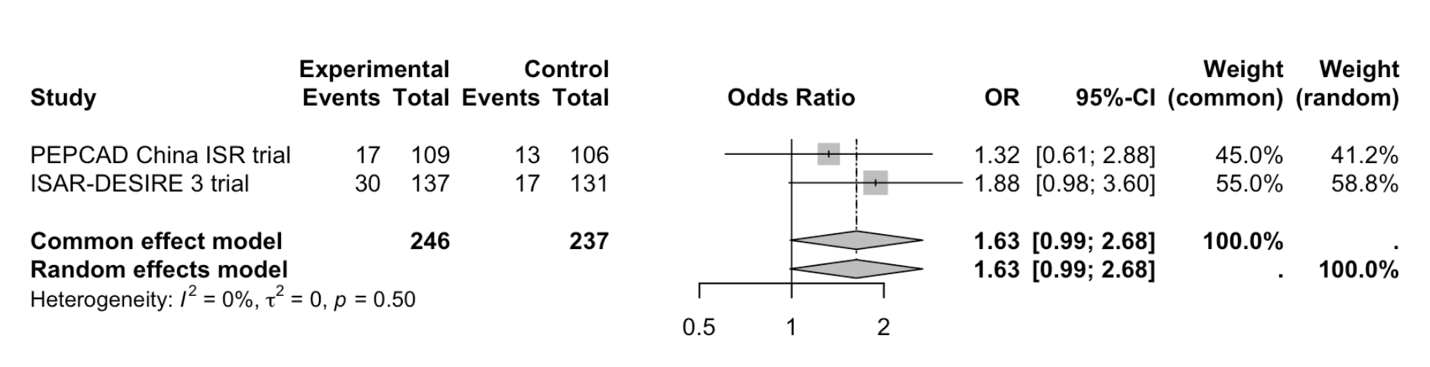


(E) PES vs PCB

(F) SCB vs PCB


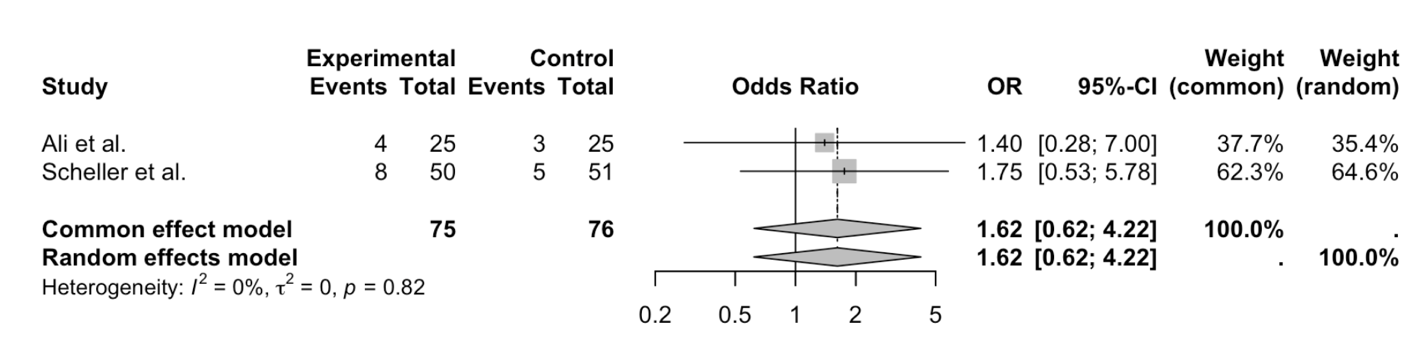


**Figure 9.** Pairwise Comparison Meta-analysis with I^2^ statistic on TLR: DCB as comparator for (A); POBA as comparator for (B) and (C); PCB as comparator for (D), (E) and (F)


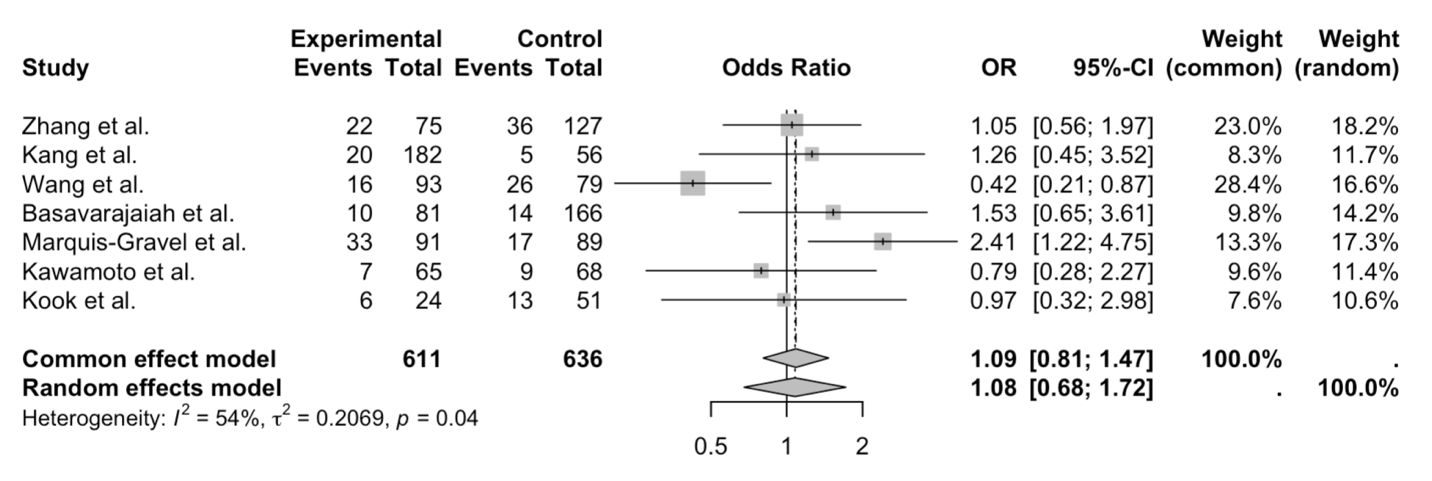


(A) DES vs DCB


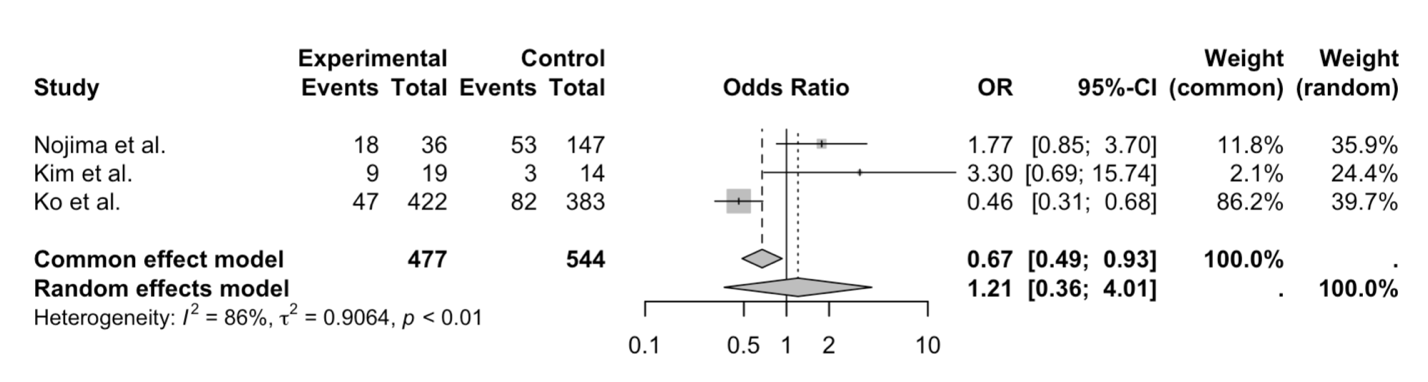


(B) DES vs POBA

(C) PCB vs POBA

(D) EES vs PCB

(E) PES vs PCB

(F) SCB vs PCB

**Figure 10.** Pairwise Comparison Meta-analysis with I^2^ statistic on MACE: DCB as comparator for (A); POBA as comparator for (B) and (C); PCB as comparator for (D), (E) and (F)

DES vs DCB

**Figure 11.** Pairwise Comparison Meta-analysis with I^2^ statistic on TLF: DCB as comparator

DES vs DCB

**Figure 12**. Pairwise Comparison Meta-analysis with I^2^ statistic on TVR: DCB as comparator

(A) DES vs DCB

(B) EES vs PCB

**Figure 13.** Pairwise Comparison Meta-analysis with I^2^ statistic on MI: DCB as comparator for (A); PCB as comparator for (B)

DES vs DCB

**Figure 14.** Pairwise Comparison Meta-analysis with I^2^ statistic on all-cause death: DCB as comparator

DES vs DCB

F**igure 15.** Pairwise Comparison Meta-analysis with I^2^ statistic on cardiac death: DCB as comparator

(A) DES vs DCB

(B) DES vs POBA

**Figure 16.** Pairwise Comparison Meta-analysis with I^2^ statistic on stent thrombosis: DCB as comparator for (A); POBA as comparator for (B)

(A) (B)

**Figure 17.** Network plot depicting the studies included for sub-analysis involving the RCTs only: (A) TLR; (B) MACE
