# Supplementary material for: Comparison of Different PCI Strategies for Coronary DES In-stent Restenosis: A Bayesian Network Meta-analysis
Source: J Soc Cardiovasc Angiogr Interv. 2025 Jan 31;4(3Part A):102428. doi: 10.1016/j.jscai.2024.102428 (PMC11993875; doi:10.1016/j.jscai.2024.102428)
Supplement: Supplementary File 2 [file mmc2.docx]

Supplementary file 2. Electronic search details

(("Coronary In-stent Restenosis" ) AND ("Balloon Angioplasty" OR "Drug coated Balloon" OR "Drug Eluting stent" OR "Rotablation" OR "Rotational atherectomy" OR "Orbital Atherectomy" OR "Intravascular Lithotripsy" OR "Coronary IVL" OR "IVL" OR "Excimer Laser Coronary Atherectomy" OR "ECLA"))

PubMed

142

<https://pubmed.ncbi.nlm.nih.gov/?term=%28%28%22Coronary+In-stent+Restenosis%22+%29+AND+%28%22Balloon+Angioplasty%22+OR+%22Drug+coated+Balloon%22+OR+%22Drug+Eluting+stent%22+OR+%22Rotablation%22+OR+%22Rotational+atherectomy%22+OR+%22Orbital+Atherectomy%22+OR+%22Intravascular+Lithotripsy%22+OR+%22Coronary+IVL%22+OR+%22IVL%22+OR+%22Excimer+Laser+Coronary+Atherectomy%22+OR+%22ECLA%22%29%29&sort=pubdate>

PubMed central

616

<https://www.ncbi.nlm.nih.gov/pmc/?term=%28%28%22Coronary+In-stent+Restenosis%22+%29+AND+%28%22Balloon+Angioplasty%22+OR+%22Drug+coated+Balloon%22+OR+%22Drug+Eluting+stent%22+OR+%22Rotablation%22+OR+%22Rotational+atherectomy%22+OR+%22Orbital+Atherectomy%22+OR+%22Intravascular+Lithotripsy%22+OR+%22Coronary+IVL%22+OR+%22IVL%22+OR+%22Excimer+Laser+Coronary+Atherectomy%22+OR+%22ECLA%22%29%29>

Scopus

168

<https://www.scopus.com/results/results.uri?sort=plf-f&src=s&st1=%28%28%22Coronary+In-stent+Restenosis%22+%29+AND+%28%22Balloon+Angioplasty%22+OR+%22Drug+coated+Balloon%22+OR+%22Drug+Eluting+stent%22+OR+%22Rotablation%22+OR+%22Rotational+atherectomy%22+OR+%22Orbital+Atherectomy%22+OR+%22Intravascular+Lithotripsy%22+OR+%22Coronary+IVL%22+OR+%22IVL%22+OR+%22Excimer+Laser+Coronary+Atherectomy%22+OR+%22ECLA%22%29%29&sid=21cea207c3d03a68dfd1b6d798b65348&sot=b&sdt=b&sl=305&s=TITLE-ABS-KEY%28%28%28%22Coronary+In-stent+Restenosis%22+%29+AND+%28%22Balloon+Angioplasty%22+OR+%22Drug+coated+Balloon%22+OR+%22Drug+Eluting+stent%22+OR+%22Rotablation%22+OR+%22Rotational+atherectomy%22+OR+%22Orbital+Atherectomy%22+OR+%22Intravascular+Lithotripsy%22+OR+%22Coronary+IVL%22+OR+%22IVL%22+OR+%22Excimer+Laser+Coronary+Atherectomy%22+OR+%22ECLA%22%29%29%29&origin=searchbasic&editSaveSearch=&yearFrom=Before+1960&yearTo=Present&sessionSearchId=21cea207c3d03a68dfd1b6d798b65348&limit=10>

Embase

217

<https://www.embase.com/#advancedSearch/resultspage/history.2/page.1/25.items/orderby.date/source>.

Cochrane library for trials

39

<https://www.cochranelibrary.com/search>
